# Vocal changes in a zebra finch model of Parkinson’s disease characterized by alpha-synuclein overexpression in the song-dedicated anterior forebrain pathway

**DOI:** 10.1371/journal.pone.0265604

**Published:** 2022-05-04

**Authors:** Cesar A. Medina, Eddie Vargas, Stephanie J. Munger, Julie E. Miller

**Affiliations:** 1 Graduate Interdisciplinary Program in Neuroscience, University of Arizona, Tucson, Arizona, United State of America; 2 Department of Neuroscience, University of Arizona, Tucson, Arizona, United States of America; 3 Department of Speech, Language, and Hearing Sciences, University of Arizona, Tucson, Arizona, United States of America; 4 Department of Neurology, University of Arizona, Tucson, Arizona, United States of America; 5 BIO5 Institute, University of Arizona, Tucson, Arizona, United States of America; Claremont Colleges, UNITED STATES

## Abstract

Deterioration in the quality of a person’s voice and speech is an early marker of Parkinson’s disease (PD). In humans, the neural circuit that supports vocal motor control consists of a cortico-basal ganglia-thalamo-cortico loop. The basal ganglia regions, striatum and globus pallidus, in this loop play a role in modulating the acoustic features of vocal behavior such as loudness, pitch, and articulatory rate. In PD, this area is implicated in pathogenesis. In animal models of PD, the accumulation of toxic aggregates containing the neuronal protein alpha-synuclein (αsyn) in the midbrain and striatum result in limb and vocal motor impairments. It has been challenging to study vocal impairments given the lack of well-defined cortico-basal ganglia circuitry for vocalization in rodent models. Furthermore, whether deterioration of voice quality early in PD is a direct result of αsyn-induced neuropathology is not yet known. Here, we take advantage of the well-characterized vocal circuits of the adult male zebra finch songbird to experimentally target a song-dedicated pathway, the anterior forebrain pathway, using an adeno-associated virus expressing the human wild-type αsyn gene, *SNCA*. We found that overexpression of αsyn in this pathway coincides with higher levels of insoluble, monomeric αsyn compared to control finches. Impairments in song production were also detected along with shorter and poorer quality syllables, which are the most basic unit of song. These vocal changes are similar to the vocal abnormalities observed in individuals with PD.

## Introduction

Parkinson’s disease (PD) is the second most common progressive neurodegenerative disease resulting in motor deficits such as hypokinesia, muscle rigidity, postural instability, and resting tremor [1,2]. One major cause of PD is alpha-synuclein (αsyn) related neuropathology. Specifically, studies link mutations, polymorphisms, and du/tri-plications in the αsyn gene, *SNCA*, to accumulation of proteinaceous aggregates. These aggregates contain insoluble αsyn and precede the formation of Lewy bodies, resulting in abnormal peripheral limb motor control [3–5]. However, given that impairments in peripheral limb movement occur late in the disease pathogenesis, the early pathological events leading to extensive αsyn induced neuropathology remain unclear.

Unlike peripheral limb motor loss, select vocal motor impairments manifest early on during the disease pathogenesis and prior to the onset of the limb motor signs in up to ninety percent of patients [6–8]. Voice deficits typically associated with PD include reduced loudness, reduced prosodic pitch (i.e., monotonous), and a hoarse voice [6,8,9–14]. Speech rate and length of mean utterance are also affected [13–17]. Therefore, understanding the underlying neurobiology of vocal-related PD symptoms is becoming increasingly critical since early detection of PD is crucial for therapeutic intervention. One strategy is to model the Parkinsonian vocal symptoms in laboratory animals.

Studies in rodents have identified links between impaired vocalizations and PD neuropathology [18–25]. For instance, vocal deficits were reported in the virally-mediated rat model overexpressing human wild-type αsyn, in a transgenic mouse model of PD overexpressing human wild-type αsyn under the broad neuronal promoter Thy-1, and in a rat model where preformed αsyn fibrils were injected directly into the striatum [26–29]. Rats injected with an adeno-associated virus (AAV) to target overexpression of human wild-type αsyn in substantia nigra, which provides input onto basal ganglia, exhibited impaired call rate and reduced call intensity [26]. The vocalizations from the Thy-1 αsyn mouse model were shorter and quieter, similar to what is seen in individuals with PD. These vocalizations deficits in the Thy-1 αsyn mouse model coincided with elevated levels of αsyn protein throughout the brain including in the basal ganglia [27]. In the preformed αsyn fibril mediated rat model, formation of end stage αsyn aggregates coincided with the vocalization deficits. These vocal deficits scaled with the extent of αsyn-related neuropathology [28,29]. However, due to a limited understanding of the neural circuitry for rodent vocalizations, especially the cortico-basal ganglia-thalamo-cortico loop, studies employing model systems with well-characterized vocal circuits that can be directly targeted are necessary for understanding diseases affecting voice and speech, including PD and Huntington’s disease [7,30,31].

**Fig 1:**
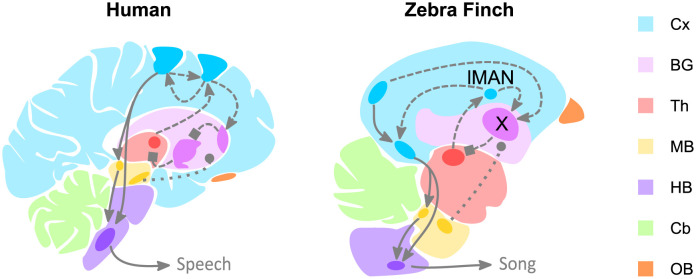
Comparison of simplified male zebra finch and human vocal motor neuroanatomy. Human and male zebra finch vocal motor pathways contain both cortico-brainstem circuits (i.e., posterior pathway) involved in vocal production (solid lines) and cortico-basal ganglia-thalamo-cortico circuits (i.e., anterior forebrain pathway, AFP) involved in vocal modulation (dashed lines). Glutamatergic (arrowhead), GABAergic (square), and dopaminergic (circle) input play critical roles within these vocal-dedicated motor pathways. In male zebra finches, however, the vocal-dedicated basal ganglia (Area X, violet) contains multiple neuronal cell types in a single nucleus, whereas in the human basal ganglia these neurons are segregated across distinct regions (light violet). Moreover, similar to humans, the finch basal ganglia receive input from cortical nuclei within the AFP. The cortical output nucleus of this pathway, lateral magnocellular nucleus of the anterior nidopallium (lMAN), receives input from a thalamic region (red, DLM) and outputs onto another cortical region (RA, bottom blue nuclei). The darker colors correspond to the remaining vocally-dedicated areas within each species’ brain. These areas have been extensively reviewed [32–35,43]. Cx—cortex. BG—basal ganglia. Th—thalamus. MB—midbrain. HB—hindbrain. Cb—cerebellum. OB—olfactory bulb.

Songbirds such as the zebra finch provide a strong model system for these studies due to the high degree of molecular, anatomical, and physiological homology to the human vocal circuit [32–35] (Fig 1). Previous studies examining impaired speech associated with mutations in genes such as *FoxP2* and *Cntnp2* have used the songbird model system to understand the role of these genes in a song-dedicated circuit important for vocal learning and on-going maintenance in adulthood known as the anterior forebrain pathway (AFP) [36–42]. Here, we utilize the zebra finch to study the vocal changes that result from expressing the human *SNCA* gene in the AFP.

The AFP in the male zebra finch is composed of a cortico-basal ganglia-thalamo-cortico-loop that is critical for modulating features of the finch’s song. We focus on a song-dedicated region homologous to the mammalian basal ganglia called Area X and one of its vocally dedicated cortical inputs—lateral magnocellular nucleus of the anterior nidopallium (lMAN) [44–47]. In this pathway, Area X receives excitatory glutamatergic input from higher order cortical regions HVC (abbreviation used as proper name) and lMAN, dopaminergic input from the substantia nigra pars compacta (SNc) and ventral tegmental areas (VTA) areas, and sends GABAergic output to the dorsolateral portion of the medial thalamus (DLM) (Fig 1) [45–47].

Previously, developmental regulation of αsyn protein was observed within the song circuit during the vocal learning period in juvenile male zebra finches, although its functional role in behavior was not explored [48,49]. Hilliard et al. [50] found that αsyn and its interacting protein, synphilin-1, were expressed within Area X. Recently, we found that levels of αsyn protein in Area X scale with vocal practice [51]. Interestingly, vocal practice (i.e., undirected singing, the amount that males sing alone without directing song to another individual) is associated with increased rendition-to-rendition variability in select acoustic features such as pitch [52–54].

**Fig 2:**
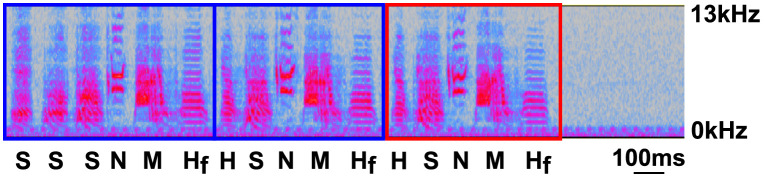
Representative spectrogram of zebra finch song. Song from an adult, male zebra finch is shown. Finches sing a characteristic song that is comprised of multiple motifs. Each blue box outlines a unique motif in that bird’s song. A red box outlines a repeated motif. Each motif is composed of a unique sequence of syllables. Four broad types of syllables include Harmonic (H), Noisy (N), Mixed (M), and Slide (S). Additionally, Flat Harmonic (H_f_) syllables were separately analyzed from general harmonic syllables. Individual acoustic features can be measured from all of these syllables (Table 1). The y axis represents frequency (Hz) and the x axis represents time (ms). Two seconds of song is shown for presentation. The frequency window is between 0 and 13 kHz. The scale bar represents 100ms.

**Fig 3:**
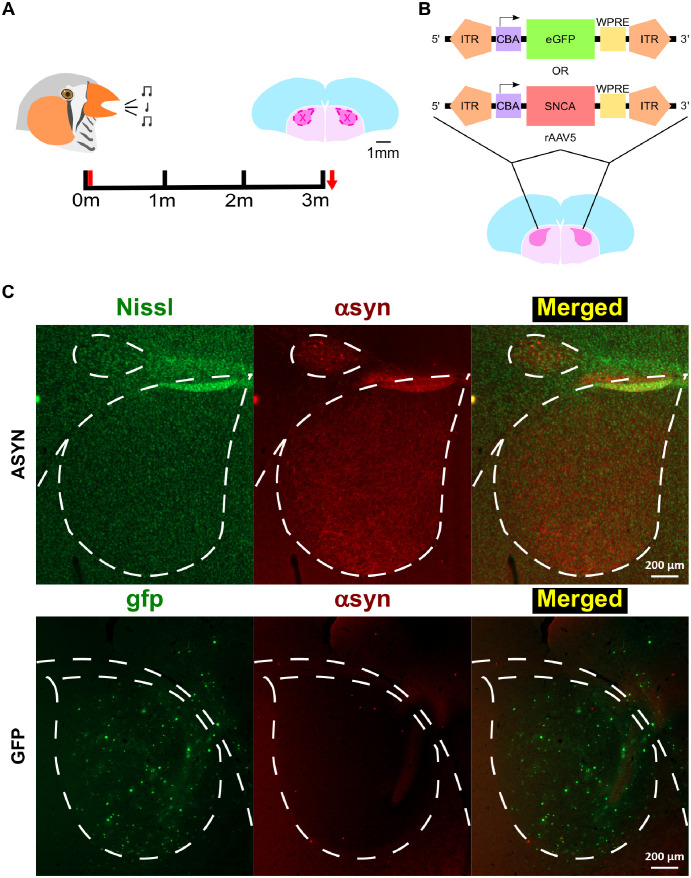
Experimental design for Parkinsonian model. **A)** Experimental timeline for characterizing song changes. Two hours of undirected (male singing alone, 2HRUD) song was recorded for at least three days during each time point over a four-month window (0, 1, 2, and 3 months, black ticks). 0 month was treated as baseline (pre-injection time point). 1, 2, and 3-month time points are post-injection. Viral injection (red tick) occurred immediately after the last morning of baseline song collection (i.e., 0 month). The brain was collected immediately following the last day of the 3-month song collection period for further tissue processing (red arrow). Area X (outlined by hot pink dashes) was biopsied for molecular validation. **B)** Schematic of recombinant adeno-associated viral construct bilaterally injected into Area X. *SNCA* virus did not contain a GFP reporter. Both viruses were under the control of the broad promoter, chicken beta-actin (CBA), driving transfection of various cell types. Either rAAV5-CBA-*SNCA* (bottom) or rAAV5-CBA-eGFP (top) were therefore bilaterally injected into Area X to monitor effects on song. **C)** Representative confocal images of αsyn levels in the zebra finch basal ganglia and cortex relative to control. A single label of αsyn protein (red) with counterstaining using a fluorescent nissl stain (green) to outline cytoarchitecture was conducted on a 2HRUD singing ASYN bird (top). A single label of αsyn protein (red) was similarly conducted on a 2HRUD singing GFP bird (bottom). Representative images demonstrate elevated αsyn protein in the ASYN group relative to control GFP finches (top relative to bottom) and highlight successful viral-mediated expression of gfp (green) in Area X but not within lMAN (bottom). Area X is demarcated by a dashed line forming the characteristic teardrop structure, while lMAN is demarcated by dashed lines forming an ellipse structure dorsal and lateral to Area X (top panel). White, dashed lines extending from or around Area X outline striatal-nidopallidal border. Top part of the ASYN and GFP images is dorsal and bottom is ventral. ASYN images are of right hemisphere, in which the left-side of the image is lateral and the right-side is medial. In contrast, GFP images are of the left hemisphere, where the left-side is medial and the right-side is lateral. Scale bar represents 200 μm. Images were taken on a Zeiss 880 Inverted Confocal Microscope.

In the current study, we developed a finch model of human wild-type αsyn overexpression using an AAV targeted within Area X to express the human *SNCA* gene in the AFP and monitored changes in the bird’s song (Figs 2 and 3A) at multiple post-injection time-points. We selected the basal ganglia region Area X over more commonly studied structures in PD such as the substantia nigra, in order to directly manipulate vocal variability. In humans, a direct link of αsyn pathology between specific cortical and basal ganglia brain regions and vocal dysfunction has not been established, therefore motivating our work in finch. We hypothesized that overexpression of αsyn in Area X would decrease the quality of the bird’s song and that the severity of this deterioration would coincide with the extent of αsyn neuropathology in the AFP. We found that increases in αsyn protein in Area X leads to impairment of select acoustic features associated with song production and quality depending on syllable type. This coincides with an increase of insoluble, monomeric αsyn in Area X and also detection of αsyn within lMAN relative to control.

## Materials and methods

### Subjects, behavior, and experimental timeline

All animal use was approved by the Institutional Care and Use Committee at the University of Arizona. Adult male zebra finches (120–400 days post-hatch) were moved into individual sound attenuation chambers (Eckel Noise Control Technologies) and acclimated under a 14:10 h light-dark cycle for at least two days prior to behavioral experiments. Behavioral experiments were conducted in the morning from lights-on until euthanasia by overdose with the inhalation anesthetic isoflurane at specified time-points to collect brain tissue. Finches were recorded singing alone (undirected, UD) for at least three consecutive mornings using Sound Analysis Pro 2011 (SAP 2011) [55,56] prior to and at one, two and three months after virus injection (Fig 3A and 3B). Song data collected and used for this study can be found at the University of Arizona ReData repository (https://doi.org/10.25422/azu.data.16619782). To characterize the effects of human wild-type αsyn overexpression on the zebra finch song system, we bilaterally injected an AAV encoding the human *SNCA* gene (AAV5-CBA-*SNCA*) into Area X. A control AAV encoding for green fluorescent protein (AAV5-CBA-eGFP) was also injected into Area X of a separate control group. A cohort of birds from both the experimental (ASYN) and control (GFP) groups were followed over the course of three months after virus injection to monitor song changes and perform immunohistochemical or Western blot analysis following tissue collection (Fig 3A and 3B).

Brain tissue for Western blotting and immunohistochemistry was obtained from birds following two hours of UD singing or two hours of no singing in the morning (2 hours non-singing, NS) at one, two or three-month post-injection time points. Previously, we showed that while overall endogenous αsyn protein levels are lower in Area X relative to surrounding striatum, two hours of UD singing increases endogenous 15kD αsyn levels in Area X the more the bird sings [51]. Therefore, this behavioral state enables validation of virally-driven αsyn overexpression in Area X (Fig 3C).

## Song recording and analysis

To determine if viral-mediated overexpression of human αsyn resulted in changes to song, birds were recorded singing alone using free-field omnidirectional microphones (Shure 93 lavalier). Song was recorded over three mornings to obtain a repertoire of the bird’s song that is later averaged. This was done to account for day-to-day variability in acoustic features when the male sings alone [54,57]. On each day of recording, song was collected for two hours immediately following lights on (beginning at 7:30 AM MST) to minimize effects of circadian rhythms on gene or protein within Area X [56]. Thus, song recorded later than 12PM MST was not analyzed. Additionally, if the bird failed to sing at least 90 motifs, which are defined as a repeated series of syllables that make up the bird’s song, or if the bird stopped singing for 20 minutes prior to the end of the collection period, then tissue collection was moved to a subsequent day (see Fig 2 for description of song). Song and tissue collection were terminated if these criteria were not met because our prior work showed that two hours of continuous UD singing is sufficient for measuring changes in levels of gene and protein within Area X [50,51].

For motif level analysis (Fig 2), the number of motifs each bird sang was manually counted by a researcher blind to the experimental condition using the open source program Audacity (https://www.audacityteam.org/). The amount of singing during each time point was calculated by averaging the total number of motifs counted across two hours of singing (Total.Motif.count), during the first or last 30 minutes (Motif.first30 and Motif.last30), and during the middle 60 minutes (Motif.mid60) on three consecutive days within a time point (pre-injection 0 time point, and post-injection 1, 2, and 3 time points). Motifs for each bird were defined from song collected during the baseline time point (pre-surgery: 0M). Motifs at the post-surgery time points were identified and counted only if they had the full complement of syllables present in the baseline motif. A normalized value for the total amount of song sung over the total two hour song session (Total.Motif.count), over the first or last 30 (Motif.first30 and Motif.last30), and during the middle 60 minutes (Motif.mid60) was generated by dividing the post month values by its respective pre-injection value.

To analyze the individual acoustic feature data (see Table 1), song was segmented using the ‘Explore and Score’ feature in SAP 2011 to generate syllable tables consisting of acoustic feature scores. Song was then clustered using the Vocal Inventory Clustering Engine (VoICE) available in Matlab using the acoustic feature scores to identify unique syllables [58]. After unique syllables were identified for each bird, 25 renditions of each syllable were randomly selected within the song collection period by a researcher blind to the experimental conditions. A sample size of 25 renditions per syllable was previously confirmed to provide sufficient power to identify experimental changes in song [53]. Three days of song were collected for a total of 75 renditions per syllable within each time point. This allowed for blind comparisons of acoustic features pre- versus post-virus injection. Syllables not reliably clustered into proper modules and syllable renditions that were improperly segmented by VoICE were excluded from further analysis. Introductory notes, unlearned calls, and other noises were also excluded.

**Table 1:** Acoustic features of birdsong extracted using Sound Analysis Pro 2011. FM is defined as angle in direction derivatives. AM is measured across all frequencies. Wiener entropy is a unit-less value. Pitch goodness units can be converted to dB after baseline subtraction and transformed to a log scale. Definitions of song features are further summarized in http://soundanalysispro.com/manual-1 and in ‘Song recording and analysis’ section of methods [55].

Acoustic Features (units)	Interpretation
**Syllable Duration (ms)**	Length of the syllable
**Amplitude (dB)**	Syllable loudness
**Pitch (kHz)**	Fundamental frequency
**Frequency Modulation**	Changes in frequency of sound
**Amplitude Modulation (kHz)**	Changes in amplitude of sound
**Wiener Entropy**	Syllable structure
**Pitch Goodness**	Changes in pitch of sound
**Mean Frequency (kHz)**	Mean frequency

For syllable level analysis of the mean acoustic features, all syllables were analyzed regardless of syllable type (‘all syllables’) or by syllable type (harmonic, noisy, mixed, or slide syllables). Syllables were further categorized based on whether they were a flat harmonic or not (i.e., all other syllables that are not a Flat Harmonic were placed into a non-flat harmonic category, Fig 2). The acoustic features measured from these syllables using the “Explore and Score” function in SAP 2011 include syllable duration (i.e., length), amplitude (i.e., loudness), pitch (i.e., fundamental frequency), frequency (i.e., mean frequency), Wiener entropy, frequency modulation (FM), amplitude modulation (AM), and pitch goodness (http://soundanalysispro.com/manual-1, Table 1) [55]. As reported on previously, amplitude scores were calibrated due to differences that could arise from fluctuations in the bird’s position relative to the microphone, which was centered above the cage. The calibrated score assumed that the bird was at the far regions of the cage. A maximum 1dB difference was noted in these far regions. Therefore, changes in sound intensity detected by the microphone that are greater than 1 dB are considered meaningful [59]. A mean and variance (var) score is measured for each of these acoustic features by SAP 2011. Mean, within-rendition variability (var), and across-rendition variability (coefficient of variation, CV) scores for select acoustic features are reported. Here, var is calculated by squaring the standard deviation in a single rendition measured from the syllable feature, whereas the CV is calculated by dividing the standard deviation of 75 renditions of a syllable by the mean score of those same renditions. Therefore, both var and CV scores reflect variability in the acoustic feature. Var represents within-rendition variability, and CV represents across-rendition variability in an acoustic feature (see Table 1 for interpretations of each acoustic features’ scores). Importantly, pitch is the fundamental frequency and frequency is the mean frequency of a syllable. FM is the change in peak frequency, where harmonics have low FM (i.e., little change) and slide syllables have high FM (i.e., change a lot). AM is a measure in the change of intensity profile of a syllable, where it is positive near the beginning and negative towards the end of a syllable. Wiener entropy (WE) is a measure of the uniformity in the power spectra (i.e., structure of the syllable), where noisy syllables have high values and harmonic syllables have low values. Lastly, pitch goodness is defined as a change in fundamental frequency and is used to identify harmonic stacks (high values). Finally, the mean, var (within-rendition), and CV (across-rendition) values were adjusted by the pre-surgery time point (0 mpi) to account for inherent inter-syllable differences, which allows us to compare different syllable types on similar scales (see **Statistics** section). For our statistical analyses, we compared scores across ASYN and GFP conditions.

For syllable level analysis of self-similarity, all syllables were analyzed regardless of syllable type (all syllables) or by syllable type (harmonic, noisy, mixed, and slide). Using the “Similarity Batch” feature in SAP 2011, 25 segmented syllables were pooled across each day of song collection from each time point to generate percent similarity (%similarity) and accuracy scores. Generally, these scores describe how a syllable varies from rendition-to-rendition by comparing the spatiotemporal properties of each syllable within a small window of time: 50ms and single millisecond time windows for %similarity and accuracy respectively, to every other rendition. More specifically, the Wiener entropy, pitch, spectral continuity, and frequency modulation of two renditions are independently measured over a user defined sliding time window. Here, we use the default SAP 2011 settings for analysis of zebra finch song. Importantly, the spectral continuity of a syllable is based on its frequency contour, where the continuity of the frequency contour is greatest for harmonic syllables and lowest for noisier syllables. It is comparable to Wiener entropy. These four features are then compared across copies of a syllable (i.e., renditions) and composite %similarity and accuracy scores are generated [55]. Comparisons of the same rendition (i.e., comparing wav 1 of syllable A to itself in one bird) were removed to ensure data was not skewed. An average %similarity and accuracy score was generated for each syllable by averaging across the three days within each time point. The %similarity and accuracy scores of syllables from ASYN and GFP conditions were compared for statistical analysis.

### Surgical procedure and virus injections

Surgery was conducted on isoflurane-anesthetized birds who received bilateral injections of either control adeno-associated virus driving expression of enhanced Green Fluorescent Protein (GFP, N = 7) or human wild-type αsyn (ASYN, N = 9) into Area X using stereotaxic coordinates from the bifurcation of the mid-sagittal sinus: 3 mm rostral, 1.62 mm medio-lateral, a depth of 3.1 mm, and head angle of 40 degrees. These coordinates avoid indirect targeting of lMAN with virus as previously described [56]. Viruses (AAV5) were made and obtained from the University of North Carolina Viral Vector Core through the support of the Michael J. Fox Foundation for Parkinson’s Research Tools: https://www.michaeljfox.org/research-tools-catalog.

A glass pipette was fitted into a Nanoject II pressure-injector and back-filled with mineral oil, then loaded with either the control or the αsyn virus. 500 nL of virus was injected at a rate of 27.6 nL/injection every 15 seconds for a total of 18 injections. After five minutes, the pipette was slowly retracted and the tip visually inspected for clogging. Birds were then returned to their sound chambers following post-operative monitoring and resumed singing within a few hours after surgery. At the one, two, and three-months post-injection (mpi), song was individually recorded in a sound attenuation chamber.

#### Tissue preparation

Virus-injected finch brains were collected immediately following two hours of UD singing unless otherwise noted and perfused prior to fixing the brain for immunohistochemistry or flash frozen in liquid nitrogen for storage at -80°C prior to Western blotting. Additional non-song control regions in finch such as the ventral striatal pallidum adjacent to Area X, as well as mouse basal ganglia and forebrain were also biopsied to obtain total or fractionated protein (see Urea Solubility Assay and Western blotting section below). For brains used in Western blotting experiments, the tissue was sectioned on a cryostat at -20°C to collect bilateral micropunches of Area X using a 20” luer adaptor (Becton-Dickinson, #22–044086) as previously described [60]. Micropunch accuracy was confirmed by inspecting thionin stained 30 μm thick coronal sections taken throughout sectioning of the punched area. Samples were excluded from the study if punches were not accurate (Fig 5b).

#### Immunohistochemistry

Perfused tissue sections were processed from adult male zebra finches bilaterally injected with either AAV-*SNCA* (N = 5 out of 9) or AAV-eGFP (N = 3 out of 7) into Area X. This approach enabled across bird comparisons. Following anesthesia, birds were transcardially perfused with warmed saline followed by chilled 4% paraformaldehyde in Dulbecco’s Phosphate Buffer Saline. Fixed brains were cryoprotected in 20% sucrose overnight then coronally sectioned between 14–30μm on a microtome cryostat through Area X. Tissue was processed, following our previously published methods [56,60]: Hydrophobic borders were drawn on the slides, using a pap pen (ImmEdge, Vector Labs) followed by 3 X 5 minute washes in TBS with 0.3% Triton X (Tx). To block non-specific antibody binding, the tissue was then incubated for one hour at room temperature with 5% goat serum (Sigma #G-9023) in TBS/0.3% Tx then 3 x 5-minute washes in 1% goat serum in TBS/0.3% Tx were performed. Primary antibodies were used against αsyn (1:250, rabbit anti-αsyn, Proteintech 10842-1-AP, RRID: AB_2192672; 1:125, mouse anti-αsyn, Thermo Fisher Scientific AHB0261, RRID: AB_2536241), a pan-neuronal marker (1:125, rabbit anti-pan-neuronal, Millipore ABN2300, RRID: AB_10953966), and GFP (1:250, mouse anti-gfp, ThermoFisher, A11120, RRID:AB_221568, S1 Fig).

Primary antibodies were incubated in a solution of 1% goat serum in TBS/0.3% Tx overnight at 4°C. A no primary antibody control was performed during initial testing. The next day, sections were washed 5 x 5-minute in TBS/0.3% Tx and incubated for three hrs at room temperature in fluorescently conjugated secondary antibodies (1:1000: goat anti-rabbit 488 Molecular Probes-ThermoFisher A11034, RRID:AB_2576217; goat anti-mouse 568 A11031, RRID: RRID:AB_144696; goat anti-rabbit 568 A11036, RRID:AB_10563566; goat anti-rabbit 647 A-21245, RRID:AB_2535813) and/or accompanied by a green fluorescent Nissl Stain (Life Technologies, N21480). After secondary incubation, sections were washed 3 x 10 minutes in TBS followed by 2 x 5 washes in filtered TBS. Slides were then cover-slipped in Pro-Long Anti-Fade Gold mounting medium (Molecular Probes, P36930) and imaged on a Zeiss 880 inverted confocal microscope or a Leica DMI 6000B fluorescence microscope with a DFC 450 color camera. The Leica DMI6000B microscope is part of the Imaging Cores—Life Sciences North, which is overseen by the University of Arizona’s Arizona Research Labs (purchase of this instrument was supported by the Arizona Technology and Research Initiative Fund, A.R.S.§15–1648). Cell counts for determining αsyn positive cells in lMAN were performed on AAV-SNCA birds (N = 3 out of 5 birds) using freeware software ImageJ (https://imagej.nih.gov/ij/) on images taken with the Leica DMI6000B.

#### Urea solubility assay

Tissue (N_ASYN_ = 4 out of 9, N_GFP_ = 4 out of 7, N_NS (non-surgical)_ = 4 out of 4) used for detecting soluble and insoluble αsyn via the urea solubility assay was immersed in 40 μL of ice-cold low salt (LS) buffer containing protease inhibitor (PI, Sigma #P8340) immediately following tissue dissection. LS buffer with PI at pH 7.5 was made containing 10mM Tris at pH 7.5, 5mM EDTA at pH 8.0, 10% sucrose, and 0.5mM phenylmethylsulfonyl fluoride (PMSF) protease inhibitor. Tissue was manually homogenized using a hand-held homogenizer. Samples were left at room temperature to incubate for 15 minutes. Following incubation, samples were centrifuged at 21,000 xg for 30 minutes. Approximately 35-40uL of supernatant was collected for the LS fraction and equal parts Laemli 2X dye (BioRad 161–0737) was added. The remaining pellet was suspended and vortexed in 20uL of urea buffer for solubilization. This is the insoluble fraction (i.e., urea soluble). The urea buffer with PI at pH 8.5 was made containing 30mM Tris, 7 M urea, 2 M Thiourea, 4% CHAPS, and 0.5mM PMSF. Equal parts Laemli 2X dye was added to the urea fraction. LS fractions were then boiled at 100°C for five minutes prior to long-term storage at -20°C. Urea fractions were not boiled prior to long-term storage in -20°C.

#### Western blotting

Prior to loading, the LS fractions were boiled again at 100°C for five minutes and the urea fractions were thawed on ice for 20 minutes. Only 40uL of fractionated samples (LS or urea w/ Laemli buffer) were loaded onto a 10% polyacrylamide gel with pre-stained molecular weight markers (BioRad 161–0394). Additional mouse or finch samples were processed via urea solubility assay or with RIPA Lysis buffer (i.e., total protein) and loaded for the control blots (see tissue preparation, S2 and S3 Figs). The gel was run at 100 volts for ~1.5 hours. Gels were then transferred onto a 0.2 μm PVDF membrane (BioRad 1620177) using a mini-trans blot cell apparatus at 400mA for 2 hours. Following transfer, Ponceau S solution (SigmaAldrich P7170-1L) was used to stain the PVDF membrane to ensure proper separation of protein that would enable incubation with multiple primary antibodies. The membrane was then blocked in 5% milk/TBS-0.3% Tween for 1 hr at room temperature with shaking. Primary antibodies were added to a 2.5% milk/TBS/Tween solution in sealed pouches shaken overnight at 4°C (1:500, rabbit anti-αsyn, Proteintech 10842-1-AP; 1:12,500, rabbit anti-GAPDH, Proteintech 10494-1-AP, RRID: AB_2263076). On the following day, blots were washed 3 x 10 minutes in TBS/Tween to remove excess primary antibody before being incubated in secondary antibodies (ECL anti-rabbit IgG HRP, 1:1000 for αsyn, 1:10,000 for GAPDH, GE Healthcare, NA934, RRID: AB_772206) for 2 hrs at room temperature followed by 5 x 10 minute TBS/Tween washes. Blots were then imaged using a chemilumescence system (Clarity Western ECL Substrate kit, BioRad 170–5060) on a BioRad ChemiDoc MP Imaging System (BioRad 17001402). Densitometric quantification of bands was done in Image Lab (BioRad) by an experimenter blind to the treatment condition. Briefly, each band was manually identified in Quantity One and a lane background subtraction was conducted within the software to yield an adjusted value. Adjusted values were obtained for αsyn, then divided by a corrected GAPDH signal within the LS lane to control for protein loading. GAPDH was selected as the loading control because it is not regulated by song [60], and we have previously validated it for detecting changes in αsyn within Area X [51]. Prior to quantification, we also confirmed that the GAPDH and other protein signals were not saturated in the raw blot images using Image Lab software by BioRad. Protein values reported in figures represent these normalized values.

### Statistics

A cohort of birds from both the ASYN and GFP control groups were followed over the course of three months after virus injection to monitor changes in the individual acoustic features of various syllable types. Mean and variance (Var) scores, which reflect within-rendition variability in the individual acoustic features (see **Song recording and Analysis section**, Table 1), were generated by SAP 2011. Each score was adjusted by dividing each of the four time points (0, 1, 2, and 3 mpi) with the pre-injection score (0 mpi). This allowed for between group comparisons (ASYN vs. GFP control) of the syllable level data at each time point because the scores are scaled. A Wilcoxon Rank Sum test was used to compare this data. The non-parametric Wilcoxon Rank Sum test was used because of the heterogeneity in data across groups. Importantly, the number of data points from each bird varies depending on the total number of syllables in their song, which can skew the data. Therefore, the nonparametric Wilcoxon Rank Sum test is critical to remain agnostic to possible skew in the individual acoustic feature comparisons. Additionally, a CV score for each feature was computed by dividing the standard deviation and its corresponding mean (i.e., CV = std. dev. / mean) from 75 renditions of the same syllable. Therefore, the CV of each individual acoustic feature reflects the across-rendition variability. Significance for all between-group comparisons of individual acoustic feature data was determined at p < 0.05. To determine whether an individual syllable’s similarity and accuracy scores changed over time (from pre-surgical time point 0, to 1, 2, 3 mpi), we ran a within group Welch ANOVA by syllable type followed by a simple linear model. A Bonferroni correction on the alpha level was not applied on any of the syllable data to avoid type-2 (false negatives) errors that could arise given that we evaluated 24 different acoustic features, but the possibility of type-1 errors is acknowledged. We also report on trends for select song data based on a p-value between 0.05 < p < 0.1.

In addition to analyzing individual acoustic features, the amount sung over a two hour song session (i.e., number of motifs sung, Total.Motif.count) and during the first/last 30 (Motif.first30 and Motif.last30, respectively) or middle 60 minutes of that song session were also counted from the first cohort. This represents the motif level analysis. Initially, a Friedman test was used to reveal differences at the motif level. Afterward, a within-group t-test that assumes unequal variance in the data was used to test for significance across time points (i.e., Motif.first30 at pre-injection time point was compared to all post-injection time points for ASYN and GFP groups separately) and within scores (e.g., Motif.first30 and Motif.last30 are compared at pre-injection and post-injection time points for both groups). Importantly, a paired t-test that assumed unequal variance (i.e., Welch’s test), was used over the nonparametric Wilcoxon Rank Sum test due to the small sample size that nonparametric tests cannot account for well. Additionally, we used a simple linear model in R to examine the relationship between how much the bird sang within a two hour or first and last 30 minute period across time points. Significance for all within-group comparisons of motif level data was determined at p < 0.05. A Bonferroni correction on the alpha level was not applied to avoid type-2 errors that could arise from small sample size constraints. We also report on trends for select song data based on a p-value between 0.05 < p < 0.1.

For a different cohort of ASYN, GFP, and NS groups used in the Western blot experiments, normalized αsyn levels were computed by dividing αsyn signal within each lane by its corresponding GAPDH signal from the LS lane (i.e., the urea signal is divided by the GAPDH signal from the same bird’s LS fraction). The normalized protein levels were compared initially using the one-way Kruskal-Wallis test because of the heterogeneity in data. Afterwards, an unpaired t-test that assumed unequal variance, the Welch’s test, was used for post-hoc comparisons amongst groups. A Bonferonni correction was not used to avoid type-2 error. Additionally, the motif level data from this Western blot cohort was run against a simple linear regression to relate levels of αsyn and features of song such as the number of motifs to characterize the relationship between behavior and protein. Significance for all across-group comparisons of protein data and simple linear regressions was determined at p < 0.05. We also report on trends based on a p-value between 0.05 < p < 0.01.

## Results

### Asyn overexpressed within the zebra finch AFP

**Fig 4:**
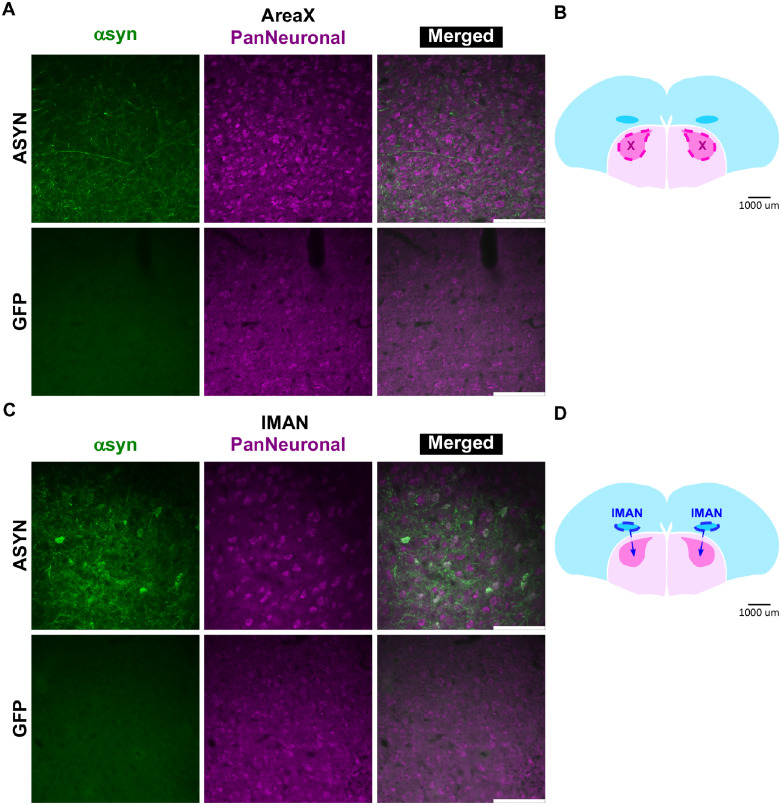
Asyn overexpression in zebra finch AFP. **A)** Representative images of an αsyn (green signal) and PanNeuronal (purple signal) double label taken from an ASYN (top) or GFP (bottom) bird following 2HRUD singing highlight overexpression of αsyn within neuronal processes in Area X. **B)** Schematic representation highlighting Area X in a coronal slice of zebra finch brain. **C)** Representative images of an αsyn and PanNeuronal double label taken from an ASYN (top) or GFP (bottom) bird highlight overexpression of αsyn within neuronal cell bodies and processes in lMAN (white signal). **D)** Schematic representation highlighting lMAN in a coronal slice of zebra finch brain. Tissue was collected from a cohort of both ASYN or GFP birds collected at 3 mpi. Images were taken near center of target region on a Leica DMI 6000B wide field fluorescence microscope with a DFC 450 color camera at 40x magnification. Scale bar represents100μm.

Previous research indicates that the basal ganglia and cortex of Parkinson’s patients are highly disrupted by αsyn neuropathology [3–5,61,62]. These areas are crucial for vocal motor control. It is unclear, however, to what extent αsyn neuropathology relates to disruption of speech and voice. We predicted that αsyn neuropathology is present throughout the basal ganglia song center Area X in human αsyn overexpressing birds. To examine αsyn expression patterns within the zebra finch song system following bilateral injection of the AAV5-CBA-eGFP/*SNCA* into Area X, we imaged coronal slices via confocal or wide field microscopy. A fluorescent Nissl stain was used in select experiments to outline the border between the nidopallium and striatum. Following the three mpi endpoint for song collection, we found that αsyn was qualitatively highly expressed within Area X processes compared to GFP controls that showed low endogenous αsyn expression (Figs 3 and 4). Levels of αsyn at one and two mpi were not assessed. We found that αsyn did not associate with neuronal cell bodies, but it is expressed in Area X processes (Fig 4A). Unexpectedly, we detected αsyn signal in lMAN neurons and processes (mean number of neurons = 10.33, sd = 3.09, N = 3 AAV5-*SNCA* finches), which is a cortical song nucleus providing input to Area X, of the ASYN group compared to control (Fig 4C). Importantly, the GFP control group showed viral-mediated gfp expression in Area X cells, but not in lMAN (S1 Fig).

#### Insoluble, monomeric αsyn is overexpressed in Area X

Previous αsyn overexpression models have shown that an increase in soluble and insoluble αsyn protein precede synuclein based neuropathology in PD [63–66]. To determine whether viral-mediated overexpression of human αsyn promotes an increase in these forms of αsyn, we conducted urea solubilization experiments that separate soluble from insoluble protein. For these experiments, levels of monomeric (~15kD) to higher molecular weight αsyn species (~250kD) were quantified across both low salt (LS) and urea soluble fractions. In our prior work, we validated that the αsyn antibody detects monomeric αsyn protein between 15-20kD [51]. Here, we show selectivity of this antibody for higher molecular weight αsyn protein between 50-250kD via a preabsorption control (S2 Fig). Furthermore, we detected αsyn protein of similar molecular weights across zebra finch Area X, mouse forebrain, and mouse basal ganglia processed through conventional assays that detect total protein (i.e., RIPA) or in soluble/insoluble fractions (S2 and S3 Figs). Higher levels of αsyn protein were detected in the basal ganglia and forebrain of Thy-1-*hSNCA* mice compared to wild type (S3 Fig).

**Fig 5:**
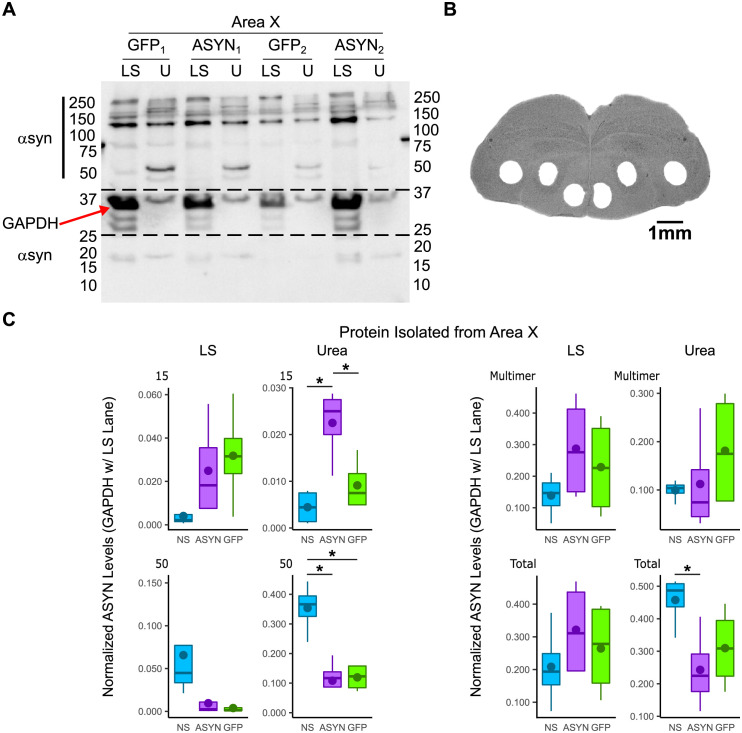
Asyn expression levels by molecular weight in Area X. **A)** A representative Western blot loaded with low salt (LS) or urea (U) soluble fractions obtained from Area X of birds injected with either AAV5-CBA-eGFP or AAV5-CBA-ASYN following 2HRUD singing is shown on the top. A blot with non-surgical (NS) birds is a supplementary figure (S4 Fig). Western blots were labelled with an αsyn antibody for quantification of protein levels in Area X relative to GAPDH from the LS lane of the same sample. **B)** A representative coronal slice of zebra finch brain highlighting punched Area X, the ventral striato-pallidum (VSP) ventral and medial to Area X, and nidopallium (NP). Tissue biopsies were taken of song-dedicated nucleus Area X and neighboring non-song related motor area VSP prior to thionin staining to confirm punch accuracy. Image from Miller et al. [56] **C)** Quantification of blots. For quantification, αsyn expression levels for each fraction are grouped by molecular weight within each group. Western blots indicate that higher levels of monomeric (15kD) urea-soluble αsyn are expressed in the ASYN group compared to the NS group and for the GFP group. Levels of trimeric (~45-50kD) αsyn protein are lower in ASYN and GFP groups compared to NS. Interestingly, total levels of urea soluble αsyn are also lower in ASYN compared to NS. Summary statistics provided in Table 2. Subscripts under experimental condition on representative blot denote different birds (i.e., sample). The representative blot contains raw data from birds 1 and 2 of both ASYN and GFP control groups (all raw blot images can be found in Supporting Information). Horizontal dashed lines indicate where blot was cut for incubating blot with either ASYN (~15kD, 40–250+kD) or GAPDH (~35kD) antibody. Names on left side of ladder indicate primary antibody used for immuno-labeling that section of the blot. For boxplots, vertical lines are 95% confidence intervals. Bottom end of the box is first quartile (25 percentile), middle horizontal line is second quartile (50 percentile), and top end of the box is the third quartile (75 percentile). The circle points represent the mean value for each group. Statistical comparisons were made using a Welch’s test. * indicate p < 0.05. # indicates 0.05 < p < 0.1.

Following validation of the αsyn antibody, levels of soluble (low salt) and insoluble (urea) αsyn protein in Area X and the adjacent non-song basal ganglia region, ventral striatal-pallidum (VSP), were quantified and compared between non-surgical control finches (S4 Fig), ASYN, and GFP groups (Fig 5, Table 2). All samples were normalized against levels of glyceraldehyde 3-phosphase dehydrogenase (GAPDH), a common loading control protein, to compare across conditions [51,60]. A Kruskal-Wallis test revealed a statistically significant difference in 15kD urea soluble αsyn (p = 0.0264, f = 7.269, df = 2, Kruskal-Wallis) in Area X. Afterwards, post-hoc Welch’s tests revealed that levels of 15kD urea soluble αsyn in this region were significantly higher in the ASYN group compared to the NS group (p = 0.013, Welch’s test) and to the GFP group (p = 0.037, Welch’s test) (Fig 5C). Intriguingly, the Kruskal-Wallis also revealed a statistically significant difference in 50kD urea soluble αsyn (p = 0.0249, f = 7.385, df = 2, Kruskal-Wallis) in Area X. A post-hoc Welch’s test revealed that levels of 50kD urea soluble αsyn were significantly lower in this region for ASYN (p = 0.005, Welch’s test) and GFP (p-value = 0.006, Welch’s test) groups compared to the NS group (Fig 5C). Lastly, the Kruskal-Wallis revealed a trend in the total amount of urea soluble αsyn (p = 0.0775, f = 5.115, df = 2, Kruskal-Wallis) in Area X. A Welch’s t-test revealed that the total amount of urea soluble αsyn protein (15kD and 45-250kD) was statistically lower in the ASYN group compared to the NS group (p = 0.031, Welch’s test, Fig 5C). Samples from VSP were also collected from each bird to ensure αsyn overexpression did not spread to this neighboring area, affecting non-song behavior (S5 Fig). No significant differences across the groups for LS and urea soluble αsyn in the VSP were found at 15kD (S5 Fig). Interestingly, the Kruskal-Wallis identified statistically significant differences in the amount of 50kD (p = 0.0264, f = 7.269, df = 2, Kruskal-Wallis) urea soluble αsyn with a trend for total (p = 0.0627, f = 5.538, df = 2, Kruskal-Wallis) in this region. Additionally, it uncovered a statistically significant effect for total (p = 0.04978, f = 6.0, df = 2, Kruskal-Wallis) and multimeric (p = 0.04978, f = 6.0, df = 2, Kruskal-Wallis) LS soluble αsyn with a trend for 50kD (p = 0.0627, f = 5.538, df = 2, Kruskal-Wallis). However, later post-hoc Welch’s tests revealed that only levels of higher molecular weight urea soluble 50kD (p-value = 0.013, Welch’s test), multimeric urea (75kD to 250kD, p = 0.03, Welch’s test) and LS (p = 0.023, Welch’s test) soluble αsyn, and total LS (p-value = 0.012, Welch’s Test) and urea (p-value = 0.013, Welch’s test) soluble αsyn were different across NS and GFP groups (S5 Fig). No differences were detected in VSP of the ASYN group to either GFP or NS groups with a post-hoc Welch’s test. This supports that our viral mediated manipulation of αsyn was constrained to the AFP.

**Table 2:** Summary statistics of normalized insoluble (Urea) αsyn levels in Area X grouped by condition and molecular weight (Mol. Wt.). Conditions and molecular weights correspond to values referenced in Fig 5.

Summary Table of Insoluble (Urea) αsyn Levels in Area X					
Mol. Wt. (KDa)	Condition	N	mean	median	sd
**15**	ASYN	4	2.25E-02	2.50E-02	7.91E-03
	GFP	4	9.12E-03	7.48E-03	5.57E-03
	NS	4	4.45E-03	4.42E-03	3.71E-03
**50**	ASYN	4	1.08E-01	1.17E-01	7.75E-02
	GFP	4	1.19E-01	1.23E-01	4.51E-02
	NS	4	3.54E-01	3.66E-01	8.52E-02
**Multimer**	ASYN	4	1.12E-01	7.43E-02	1.09E-01
	GFP	4	1.81E-01	1.75E-01	1.21E-01
	NS	4	9.93E-02	1.04E-01	2.09E-02
**Total**	ASYN	4	2.43E-01	2.25E-01	1.22E-01
	GFP	4	3.10E-01	3.09E-01	1.24E-01
	NS	4	4.58E-01	4.87E-01	7.97E-02

### Asyn overexpression affects song production

**Fig 6:**
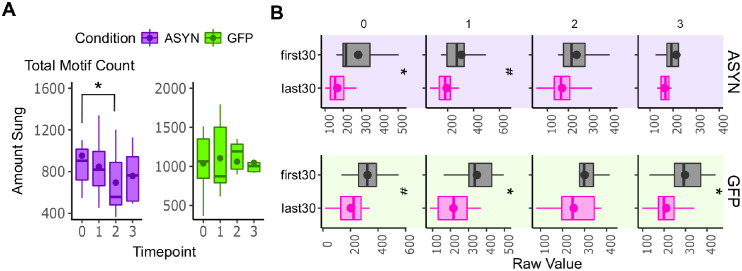
Song production affected by αsyn overexpression in the AFP. **A)** Total amount sung affected by αsyn overexpression. ASYN birds sing less at 2 mpi compared to pre-injection. No within-group differences were detected across time points for GFP birds (N = 6). For boxplots, vertical lines are 95% confidence intervals. Bottom end of the box is first quartile (25 percentile), middle horizontal line is second quartile (50 percentile), and top end of the box is the third quartile (75 percentile). The circle points represent the mean value for each group. **B)** Singing during the first 30 minutes of a two-hour singing session is affected by αsyn overexpression. Amount sung during the first 30 and last 30 minutes of the two hour singing period during 0, 1, 2, and 3 months-post injection plotted for both ASYN and GFP groups. GFP birds tend to sing less during the last 30 minutes (Motif.last30) compared to the first 30 minutes (Motif.first30) at the pre-injection time point (0 mpi). The effect persists in the GFP birds during one and three mpi time point while they sang less during the last 30 minutes at 2 mpi compared to first 30, but the effect is not significant. ASYN birds similarly sing significantly less during the last 30 minutes compared to first 30 minutes at the pre-injection time point (0 mpi) but this ends at one mpi. Summary statistics provided in Tables 3 and 4. The raw values are plotted. For boxplots, horizontal lines are 95% confidence intervals. Left end of the box is first quartile (25 percentile), middle vertical line is second quartile (50 percentile, median), and right end of the box is the third quartile (75 percentile). The circle points represent the mean value for each group. Statistical comparisons were made using a Welch’s test. * indicates p < 0.05. # indicates 0.05 < p < 0.1.

Parkinson’s patients can present with problems in producing speech, such as reduced length of mean utterance and a decline in speech rate [13,17]. We predicted that birds overexpressing αsyn in Area X would sing less and have issues in timing of their song. To assess the effects of αsyn overexpression on song production (i.e., how much the bird sang), we counted the number of motifs that ASYN and GFP groups sang during the total two-hour window (Total Amount Sung, Fig 6A) plus the first and last 30 (first30 and last30 respectively, Fig 6B) minutes. Initially, a Friedman test revealed a statistical trend for total amount sung over the entire two-hour song session across time points (p = 0.0503, f = 7.8, df = 3, N = 7, Friedman test, Fig 6A, Table 3) in the ASYN group. A post-hoc Welch Test revealed that the ASYN group sang less during the total two-hour session at two mpi compared to 0 mpi (Total.Motif.count, p = 0.02, Welch’s test, Fig 6A, Table 3). No significant difference was observed in the GFP control group for amount sung over the total two-hour period when comparing the post-injection time points to pre-injection (Fig 6A, Table 3). Previous studies have also explored whether Parkinson’s patients present with vocal fatigue (i.e., speaking less or with worse quality over a specified time window) [67,68]. Typically, adult finches sing less UD song over the course of a day with singing peaking at the beginning of the morning then decreasing [69,70]. Importantly, our lab previously showed that αsyn levels are positively correlated with how much finches sing [51]. Taken together, we predicted that this naturally occurring effect would be impaired in birds producing higher levels of insoluble αsyn within the AFP and that this would be related to αsyn levels. Initially, a Friedman test revealed a statistically significant difference in the amount sung during the first 30 minutes across all time points within the ASYN group (p = 0.0133, f = 10.7143, df = 3, N = 7, Friedman Test, Fig 6B, Table 4). We found that the ASYN group sang less during the first 30 minutes of the song session at three mpi compared to its pre-injection time point (first30 at 0 mpi compared to the first30 at 3 mpi, p = 0.03, Welch test). This finding is consistent with our simple linear model results showing that the amount sung during the first 30 minutes was trending downward in the ASYN (p = 0.0512, statistics = -2.04434, estimate = -1.8556, N = 7, df = 3) but not in the GFP group.

**Table 3:** Summary statistics for total amount sung over a two-hour song session. Month values correspond to time point values in Fig 6.

Summary Table of Total Amount Sung								
Month	ASYN				GFP Control			
	N	mean	median	sd	N	mean	median	sd
**0**	7	953.38	905.00	395.45	6	1038.85	1064.67	423.35
**1**	7	847.17	819.67	295.18	6	1105.22	873.17	504.13
**2**	7	695.07	558.33	310.23	6	1061.50	1192.67	348.24
**3**	7	758.19	764.33	255.15	6	1047.64	1003.92	460.10

**Table 4:** Summary statistics for amount sung during first and last 30 minutes of two-hour song session. Month values correspond to time point values in Fig 6.

Summary Table of Amount Sung During First and Last 30 Minutes								
Condition	Month	N	First 30			Last 30		
			mean	median	sd	mean	median	sd
**ASYN**	0	7	281.21	214.67	121.21	181.52	189.33	58.25
	1	7	262.90	208.00	121.84	178.31	180.50	70.87
	2	7	216.81	187.33	65.77	168.38	146.33	84.91
	3	7	213.48	193.00	83.52	162.52	166.67	55.52
**GFP Control**	0	6	333.83	322.33	139.10	195.21	223.17	119.52
	1	6	344.31	335.00	112.15	216.47	216.83	121.69
	2	6	300.44	298.50	83.36	250.61	245.17	112.88
	3	6	297.61	291.83	111.56	210.44	199.00	83.12

We also compared how much birds sang across the first and last 30 minutes of a two-hour song session at pre-injection, one, two, and three mpi within both the ASYN and GFP control groups (Fig 6B, Table 4). When comparing the amount sung during the first to the last 30 minutes of a singing period within each group, we found that the GFP control group sang more during the first 30 minutes of a two-hour song session than during the last 30 minutes across the one (p = 0.0153, t-test) and three (p = 0.0218, t-test) mpi time points (Fig 6B) with a trend observed for the GFP group during the pre-injection time point (0 month, p = 0.0517, t-test). In contrast, the ASYN group only sang more during the first 30 minutes of the pre-injection (0 month, p = 0.0129, t-test) time point with a trend at one mpi (p = 0.0692, t-test). By the two and three mpi time point, the ASYN group did not sing significantly more during the first 30 minutes than in the last 30 minutes (Fig 6B).

#### Soluble αsyn expression is positively correlated with song production

Previously, we found that total levels of endogenous monomeric 15kD αsyn protein are positively correlated with increased singing [51]. To examine the relationship between levels of αsyn protein to song production, we correlated levels of each αsyn species (i.e., 15kD, 50kD, 75kD, 150kD, and 250kD) plus aggregate scores for multimers and total protein across both the soluble and insoluble fractions to total amount sung over two hours (Total.Motif.count), first 30 minutes (Motif.first30), middle 60 minutes (Motif.mid60), and last 30 minutes (Motif.last30) of the two hour period using a simple linear model. Extending beyond our previous findings, we found that the amount birds sing during the first 30 minutes (p = 4.44 x 10^−2^, statistic = 2.30, estimate = 8.23 x 10^−5^, adj. R^2^ = 2.81 x 10^−1^, sigma = 1.79 x 10^−2^, residual = 10) and middle 60 minutes (p = 2.72 x 10^−2^, statistic = 2.59, estimate = 5.02 x 10^−5^, adj. R^2^ = 3.41 x 10^−1^, sigma = 1.72 x 10^−2^, residual = 10) are positively related with levels of LS soluble, monomeric (15kD) αsyn with a trend observed for the total two-hour session (p = 5.23 x 10^−2^, statistic = 2.20, estimate = 2.63 x 10^−5^, adj. R^2^ = 2.59 x 10^−1^, sigma = 1.82 x 10^−2^, residual = 10). This corroborates our song production results, detecting differences in amount sung during the first 30 minutes of the song session. No other significant relationships were detected.

#### Asyn overexpression changes the acoustic structure of song depending on syllable type

Previous studies have shown that PD is associated with a decrease in vocal quality (e.g., changes in pitch, loudness, etc) [reviewed in: 7]. To assess the effects of αsyn overexpression on vocal quality, we compared the adjusted value representing the ratio between post-injection and pre-injection values of acoustic features from all individual syllables across both the ASYN and GFP control conditions. To account for day-to-day variability in song, 25 renditions of each syllable were taken beginning with the first complete motif utterance following lights-on in the morning and averaged across three mornings at each time point [53,54]. Individual syllables were also categorized by their structure as noisy, mixed, harmonic, or slide (Fig 2) based on previous reports, to assess if there were syllable type dependent changes on song [59]. Mean and coefficient of variation (CV) scores are calculated for each feature. The CV of the mean acoustic features denotes between syllable variability and is calculated by dividing the standard deviation with the mean. Separate SAP-generated variance measures refer to within-syllable variability (e.g., var pitch is the amount that pitch varies in a single utterance or rendition of the syllable). All three types of scoring are important for understanding the types of changes that can result from experimental manipulation.

**Fig 7:**
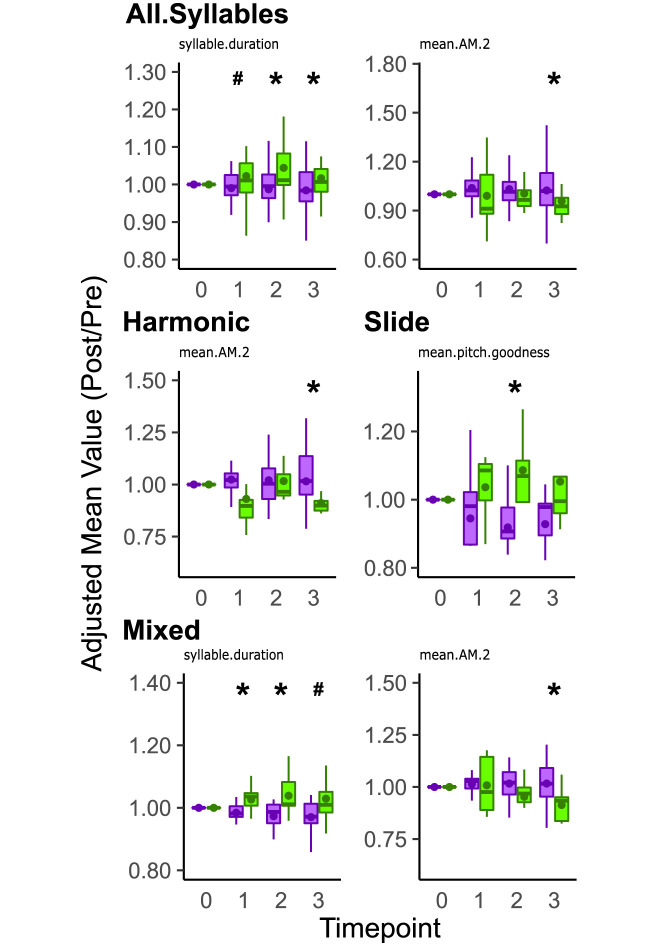
Asyn overexpression differentially affects select acoustic features depending on syllable type. The adjusted mean value of select individual acoustic features is plotted for all syllables (top) sung by αsyn and gfp expressing groups (top). Overall, the ASYN group’s syllables were shorter (syllable.duration) at two and three mpi compared to the GFP control group (top left). A trend was detected at one mpi between the ASYN group and GFP control group. Mean amplitude modulation (mean.AM.2) was higher in the ASYN group compared to GFP control group at 3 mpi (top right). The adjusted mean value of select individual acoustic features is plotted for Harmonic (middle left), Slide (middle right), and Mixed (bottom) syllables sung by asyn and gfp expressing groups. The amplitude modulation (mean.AM.2) of harmonic syllables sung by the ASYN group was higher than the GFP control group at 3 mpi. The pitch goodness of slide syllables was lower in the ASYN group compared to GFP control at 2 mpi. The duration of mixed syllables was shorter in the ASYN group compared to GFP control at 1 and 2 mpi with a statistical trend at 3 mpi (bottom left). Additionally, the amplitude modulation of mixed syllables was higher for the ASYN group compared to GFP control at 3 mpi (bottom right). Lastly, for noisy syllables, no differences were found in the ASYN group (N = 7) compared to GFP control group (N = 7, S7 Fig). Remaining acoustic features that were not affected by αsyn overexpression are reported in S6 and S7. The adjusted values are plotted for between group comparisons. Summary statistics provided in Table 5. For boxplots, vertical lines are 95% confidence intervals. Bottom end of the box is first quartile (25 percentile), middle horizontal line is second quartile (50 percentile), and top end of the box is the third quartile (75 percentile). The circle points represent the mean value for each group. Purple boxplots represent data from ASYN birds, whereas green boxplots represent data from GFP birds. Statistical comparisons were made using a Wilcoxon Rank Sum Test. * indicates p < 0.05. # indicates 0.05 < p < 0.1.

When evaluating the individual acoustic features of syllables, the mean syllable duration of all syllables was lower at two (p = 0.0039, Wilcoxon Rank Sum Test) and three (p = 0.0484, Wilcoxon Rank Sum Test) mpi in the ASYN group compared to the GFP control with a trend observed at one mpi (p = 0.08, Wilcoxon Rank Sum Test, Fig 7, Table 5). Mean amplitude modulation (mean.AM.2) was greater for the ASYN group compared to GFP controls at three mpi (p = 0.0479, Wilcoxon Signed Rank Test, Fig 7). No other differences were found for all syllables (S6 Fig). Given our prior research finding specific age-dependent differences in song by syllable type, we compared acoustic features of harmonic, noisy, mixed, and slide syllables. We found that the amplitude modulation of harmonic syllables was significantly greater in the ASYN than GFP control group at three mpi (p = 0.038, Wilcoxon Rank Sum Test, Fig 7). No effects were found for noisy syllables (S7 Fig). For mixed syllables, the duration of mixed syllables was significantly shorter (i.e., lower) in ASYN compared to GFP controls at one (p-value = 0.0255, Wilcoxon Rank Sum Test) and two (p = 0.0284, Wilcoxon Rank Sum Test) mpi with a trend observed for the three (p = 0.0645, Wilcoxon Rank Sum Test) mpi (Fig 7). The amplitude modulation of the mixed syllables was also significantly greater in the ASYN than GFP control at 3 mpi (p = 0.0099, Wilcoxon Rank Sum Test, Fig 7). For slide syllables, the pitch goodness was significantly lower in the ASYN group compared to GFP control at two mpi (p = 0.0265, Wilcoxon Rank Sum Test, Fig 7). Interestingly, the mean duration of all non-flat harmonic syllables (i.e., NotFlatHarmonics) was shorter (i.e., lower) in the ASYN group compared to GFP control at one (p = 0.0252, Wilcoxon Rank Sum Test), two (p = 0.0043, Wilcoxon Rank Sum Test), and three (p = 0.0380, Wilcoxon Rank Sum Test) mpi (S8 Fig). No other differences were detected.

**Table 5:** Summary statistics for select acoustic features grouped by syllable type. Variable column corresponds to acoustic features. Duration corresponds to syllable duration. Amp Mod corresponds to amplitude modulation. Goodness correspond to pitch goodness. Reference Table 1 for descriptions of individual variables.

Summary Table of Acoustic Features											
	Variables	Month	ASYN				GFP Control				p
			N	mean	median	sd	N	mean	median	sd	
**All**	Duration	1	55	9.90E-01	9.95E-01	6.01E-02	29	1.02E+00	1.01E+00	8.59E-02	#
		2	55	9.87E-01	9.96E-01	6.81E-02	29	1.04E+00	1.01E+00	8.84E-02	*
		3	55	9.84E-01	9.84E-01	7.28E-02	29	1.02E+00	1.01E+00	6.84E-02	*
	Amp Mod	1	55	1.04E+00	1.02E+00	1.07E-01	29	9.91E-01	9.12E-01	1.69E-01	
		2	55	1.03E+00	1.02E+00	1.24E-01	29	1.00E+00	9.65E-01	1.27E-01	
		3	55	1.02E+00	1.02E+00	1.54E-01	29	9.59E-01	9.26E-01	1.32E-01	*
**Harmonic**	Amp Mod	1	17	1.02E+00	1.02E+00	9.44E-02	8	9.31E-01	8.97E-01	1.71E-01	
		2	17	1.02E+00	1.00E+00	1.26E-01	8	1.02E+00	9.65E-01	1.09E-01	
		3	17	1.02E+00	1.02E+00	1.80E-01	8	9.11E-01	8.99E-01	5.15E-02	*
**Mixed**	Amp Mod	1	18	1.01E+00	1.03E+00	5.94E-02	9	1.01E+00	9.76E-01	1.36E-01	
		2	18	1.02E+00	1.02E+00	7.42E-02	9	9.53E-01	9.69E-01	9.53E-02	
		3	18	1.02E+00	1.02E+00	1.13E-01	9	9.14E-01	9.35E-01	7.54E-02	*
	Duration	1	18	9.84E-01	9.82E-01	3.77E-02	9	1.03E+00	1.04E+00	4.32E-02	*
		2	18	9.73E-01	9.87E-01	4.77E-02	9	1.04E+00	1.01E+00	7.11E-02	*
		3	18	9.70E-01	9.72E-01	5.57E-02	9	1.03E+00	1.01E+00	7.77E-02	#
**Slide**	Goodness	1	13	9.45E-01	9.81E-01	1.56E-01	5	1.04E+00	1.09E+00	1.05E-01	
		2	13	9.19E-01	9.07E-01	1.37E-01	5	1.09E+00	1.07E+00	1.13E-01	*
		3	13	9.28E-01	9.78E-01	1.42E-01	5	1.05E+00	9.96E-01	1.64E-01	

* indicates p < 0.05.

^#^ indicates 0.05 < p < 0.1.

**Fig 8:**
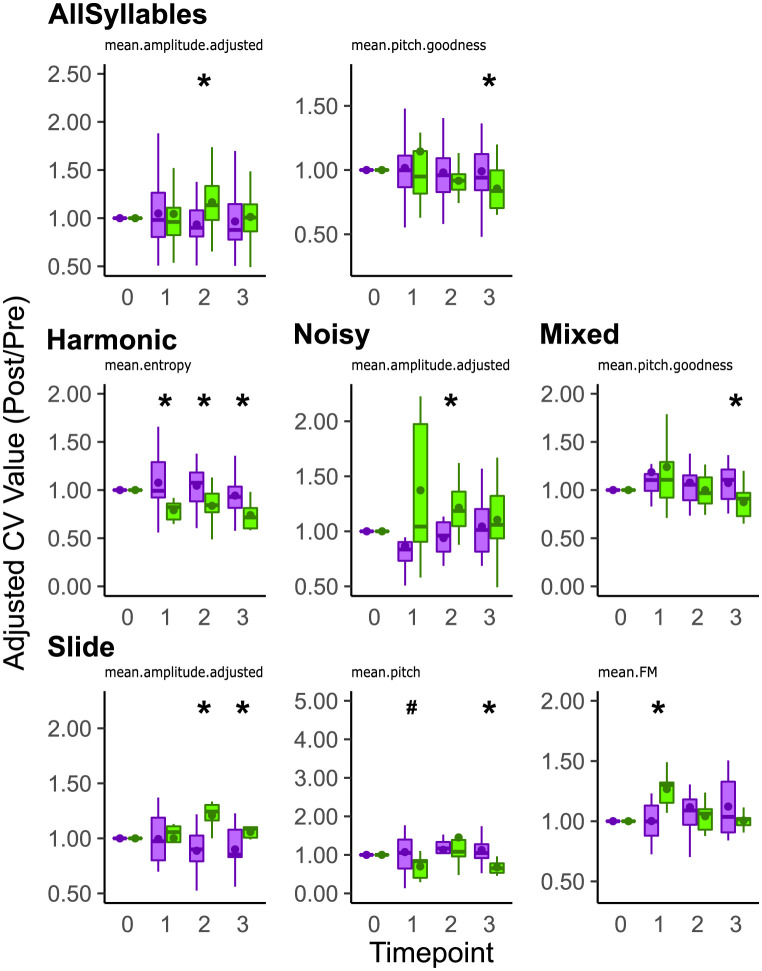
Asyn overexpression differentially affects across-rendition variability in select acoustic features depending on syllable type. The adjusted value of across rendition variation (i.e., CV, rendition-to-rendition variability) in select acoustic features is plotted for all individual syllables sung by ASYN (purple) and GFP (green) groups. Overall, the CV of amplitude (mean.amplitude.adjusted) for syllables sung by the ASYN group was lower than the GFP control group at 2 mpi (top left). Additionally, the CV of pitch goodness (mean.pitch.goodness) was higher in the ASYN group than in the GFP group at 3 mpi (top middle). The adjusted value of across rendition variation in select acoustic features is also plotted for Harmonic (middle left), Noisy (middle center), Mixed (middle right), and Slide (bottom) syllables. For harmonic syllables, the CV of entropy (mean.entropy) was significantly higher in the ASYN group compared to GFP control at 1, 2, and 3 mpi. For noisy syllables, the CV of amplitude (mean.amplitude.adjusted) was lower in the ASYN group than in the GFP control at 2 mpi. For mixed syllables, the CV of pitch goodness (mean.pitch.goodness) was higher in the ASYN group compared to the GFP control at 3 mpi For slide syllables, the CV of amplitude (mean.amplitude.adjusted) was lower in the ASYN group compared to GFP control at 2 and 3 mpi (bottom left). The CV of pitch (mean.pitch) was higher for the slide syllables in the ASYN group compared to GFP control at 3 mpi with a trend observed at 1 mpi (bottom middle). The CV of frequency modulation (mean.FM) for slide syllables was lower in the ASYN group compared to GFP control at 1 mpi (bottom right). Remaining acoustic features whose CV scores were not affected by αsyn overexpression are reported in S9-11. Summary statistics provided in Table 6. Reference Fig 7‘s legend for explanation of boxplots. Statistical comparisons were made using a Wilcoxon Rank Sum Test. * indicates p < 0.05. # indicates 0.05 < p < 0.1.

When evaluating the variation of these acoustic features across multiple song renditions using the CV score (i.e., across rendition variation), we found that the CV of amplitude (mean.amplitude.adjusted) was lower in ASYN compared to GFP controls at two mpi for all syllables (p = 0.0009, Wilcoxon Rank Sum Test, Fig 8, Table 6). Additionally, the CV of pitch goodness was significantly higher in the ASYN group compared to GFP controls at three mpi (p = 0.0103, Wilcoxon Rank Sum Test, Fig 8). No other differences were found (S9 Fig). After grouping this across-variability score by syllable level, the CV of entropy (mean.entropy) for harmonic syllables was greater in the ASYN group compared to GFP at one (p = 0.0013, Wilcoxon Rank Sum Test), two (p = 0.0429, Wilcoxon Rank Sum Test), and three (p = 0.0197, Wilcoxon Rank Sum Test) mpi (Fig 8). For noisy syllables, the CV of amplitude was significantly lower in the ASYN group compared to GFP control at two mpi (p = 0.0389, Wilcoxon Rank Sum Test, Fig 8). For mixed syllables, the CV of pitch goodness was statistically higher at three (p = 0.0152, Wilcoxon Rank Sum Test) mpi in ASYN compared to GFP control (Fig 8). For slide syllables, the CV of amplitude was lower at two (p = 0.0022, Wilcoxon Rank Sum Test) and three (p = 0.0225, Wilcoxon Rank Sum Test) mpi (Fig 8). Slide syllables also had significantly higher CV of pitch in the ASYN group compared to GFP control at three mpi (p = 0.0261, Wilcoxon Rank Sum Test) with a trend observed at one mpi (p = 0.097, Wilcoxon Rank Sum Test, Fig 8). Additionally, the CV of frequency modulation (mean.FM) for slide syllables was lower at one mpi (p = 0.0162, Wilcoxon Rank Sum Test) in the ASYN birds compared to GFP control (Fig 8). The CV of mean entropy for flat harmonic syllables was greater for the ASYN group compared to GFP control at one (p = 0.0030, Wilcoxon Rank Sum Test), two (p = 0.0409, Wilcoxon Rank Sum Test), and three (p = 0.0172, Wilcoxon Rank Sum Test) mpi but not for every other syllable type (S10 and S11 Figs). For non-flat harmonic (NotFlatHarmonic), the CV of amplitude was significantly lower at two mpi (p = 0.0015, Wilcoxon Rank Sum Test) with a trend observed at three mpi (p = 0.0979, Wilcoxon Rank Sum Test; S11 Fig). The CV of pitch goodness was also significantly higher at three mpi for the non-flat harmonic syllables from the ASYN group (p = 0.0414, Wilcoxon Rank Sum Test, S11 Fig). No other significant differences for the CV of mean individual acoustic features were detected (S10 and S11 Figs).

**Table 6:** Summary statistics of across-rendition variability within select acoustic features grouped by syllable type. Entropy corresponds to Wiener entropy. Freq Mod corresponds to frequency modulation. Reference Table 5‘s legend for additional variable names.

Summary Table of Acoustic Feature Across-rendition Variability											
	Variables	Month	ASYN				GFP Control				p
			N	mean	median	sd	N	mean	median	sd	
**All**	Amplitude	1	55	1.05E+00	9.82E-01	3.44E-01	29	1.04E+00	9.61E-01	4.21E-01	
		2	55	9.35E-01	9.00E-01	2.23E-01	29	1.17E+00	1.13E+00	3.12E-01	*
		3	55	9.67E-01	8.78E-01	2.89E-01	29	1.01E+00	1.01E+00	2.58E-01	
	Goodness	1	55	1.02E+00	9.98E-01	3.13E-01	29	1.14E+00	9.49E-01	6.69E-01	
		2	55	9.81E-01	9.59E-01	2.25E-01	29	9.16E-01	9.18E-01	2.28E-01	
		3	55	9.91E-01	9.41E-01	2.52E-01	29	8.56E-01	8.37E-01	2.17E-01	*
**Harmonic**	Entropy	1	17	1.08E+00	9.92E-01	2.81E-01	8	7.91E-01	8.22E-01	1.07E-01	*
		2	17	1.05E+00	1.08E+00	2.20E-01	8	8.37E-01	8.43E-01	2.18E-01	*
		3	17	9.43E-01	9.28E-01	2.38E-01	8	7.40E-01	7.16E-01	1.57E-01	*
**Noisy**	Amplitude	1	7	8.67E-01	8.35E-01	3.00E-01	7	1.37E+00	1.04E+00	6.65E-01	
		2	7	9.39E-01	9.61E-01	1.71E-01	7	1.21E+00	1.19E+00	2.57E-01	*
		3	7	1.04E+00	1.01E+00	3.06E-01	7	1.10E+00	1.06E+00	3.79E-01	
**Mixed**	Goodness	1	18	1.19E+00	1.10E+00	4.31E-01	9	1.24E+00	1.11E+00	4.98E-01	
		2	18	1.08E+00	1.06E+00	2.59E-01	9	9.99E-01	9.68E-01	1.68E-01	
		3	18	1.07E+00	1.11E+00	1.93E-01	9	8.75E-01	9.10E-01	1.75E-01	*
**Slide**	Amplitude	1	13	9.94E-01	9.74E-01	2.29E-01	5	1.00E+00	1.06E+00	1.57E-01	
		2	13	8.87E-01	9.00E-01	1.94E-01	5	1.21E+00	1.24E+00	1.33E-01	*
		3	13	9.00E-01	8.53E-01	2.07E-01	5	1.06E+00	1.09E+00	5.62E-02	*
	Pitch	1	13	1.07E+00	1.05E+00	4.98E-01	5	7.00E-01	8.34E-01	3.39E-01	#
		2	13	1.13E+00	1.17E+00	5.49E-01	5	1.45E+00	1.08E+00	1.11E+00	
		3	13	1.12E+00	1.05E+00	5.73E-01	5	6.77E-01	6.51E-01	2.00E-01	*
	Freq Mod	1	13	1.00E+00	9.97E-01	1.62E-01	5	1.27E+00	1.30E+00	1.62E-01	*
		2	13	1.12E+00	1.09E+00	2.45E-01	5	1.04E+00	1.06E+00	1.43E-01	
		3	13	1.12E+00	1.04E+00	2.27E-01	5	1.01E+00	1.02E+00	7.68E-02	

* indicates p < 0.05.

^#^ indicates 0.05 < p < 0.1.

**Fig 9:**
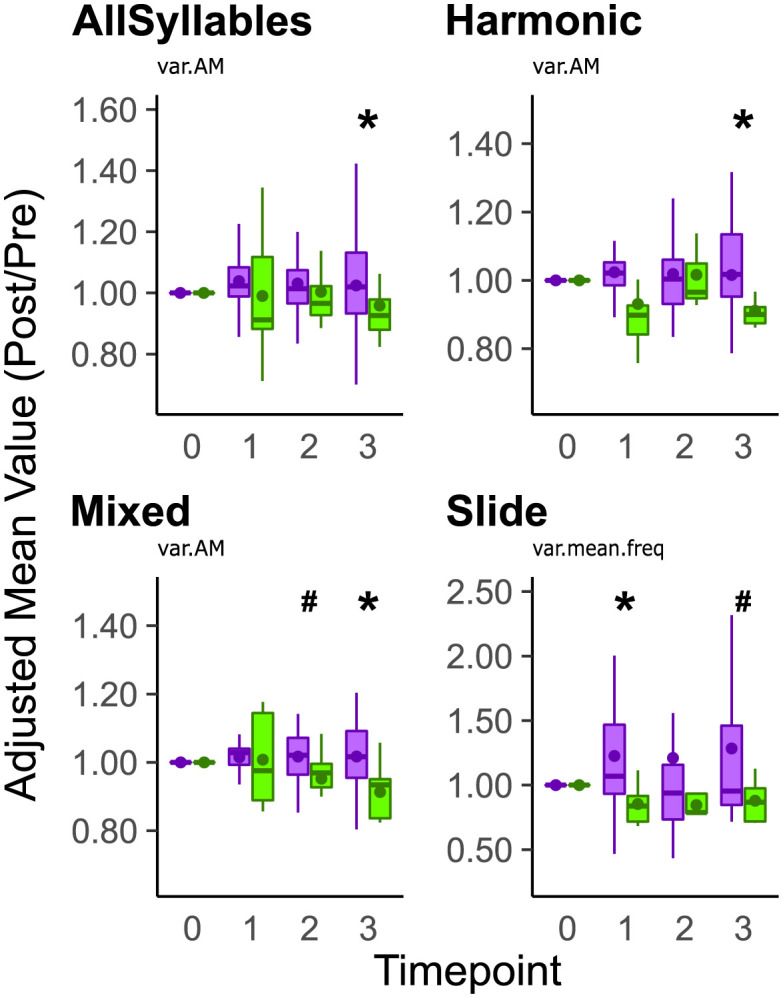
Asyn overexpression differentially affects within-rendition variability in select acoustic features depending on syllable type. The adjusted value of within rendition variation (i.e., var) in acoustic features is plotted for all individual syllables sung by ASYN (purple) and GFP (green) expressing groups (top left). Only the variance in amplitude modulation (var.AM) was statistically greater in the ASYN group compared to GFP control at 3 mpi. The adjusted value of within rendition variation (i.e., var) in acoustic features is plotted for Harmonic (top right), Mixed (bottom left), and Slide (bottom right) syllables. For harmonic syllables, the variance of amplitude modulation (var.AM) was greater in the ASYN group compared to GFP control at 3 mpi. For mixed syllables, the variance of amplitude modulation was also greater in the ASYN group compared to GFP control at 3 mpi with a trend observed at 2 mpi. For slide syllables, the variance of frequency (var.mean.freq) was greater in the ASYN group than in the GFP control at 1 mpi with a trend detected at 3 mpi. Lastly, for noisy syllables, no differences were found in the ASYN group (N = 7) compared to GFP control group (N = 7, **S12**). Remaining acoustic features whose variance scores were not affected by αsyn overexpression are reported in S12. Summary statistics provided in Table 7. Reference Fig 7‘s legend for explanation of boxplots. Statistical comparisons were made using a Wilcoxon Rank Sum Test. * indicates p < 0.05. # indicates 0.05 < p < 0.1.

When comparing within-rendition changes in acoustic features of syllables using the variance scores (var), the variance in amplitude modulation (var.AM) was greater for all syllables in the ASYN group compared to GFP control at three mpi (p = 0.0439, Wilcoxon Rank Sum Test, Fig 9, Table 7; S12 Fig). When grouping by syllable type, the variance in amplitude modulation of harmonic syllables was significantly higher at three mpi (p = 0.0374, Wilcoxon Rank Sum Test, Fig 9). While no effects were found for noisy syllables (S12 Fig), for mixed syllables, the variance in amplitude modulation was significantly higher at three mpi (p = 0.0093, Wilcoxon Rank Sum Test) with a trend observed at two mpi (p = 0.0985, Wilcoxon Rank Sum Test) in the ASYN group compared to GFP control (Fig 9). For slide syllables, the variance in frequency was significantly higher at one mpi (p = 0.0254, Wilcoxon Rank Sum Test) when comparing the ASYN group to GFP control with a trend observed at three mpi (p = 0.0641, Wilcoxon Rank Sum Test, Fig 9). The variance in frequency modulation of non-flat harmonic syllables was also significantly different at two mpi (p = 0.029, Wilcoxon Rank Sum Test) in ASYN compared to GFP controls with a trend at three mpi (p = 0.0611, Wilcoxon Rank Sum Test, S13 Fig). No other differences for variance of the individual acoustic features were detected (S12 Fig).

**Table 7:** Summary statistics of within-rendition variability within select acoustic features grouped by syllable type. Frequency corresponds to mean frequency. Reference Table 5‘s legend for additional variable names.

Summary Table of Acoustic Feature Within-rendition Variability											
	Variables	Month	ASYN				GFP Control				p
			N	mean	median	sd	N	mean	median	sd	
**All**	Amp Mod	1	55	1.04E+00	1.02E+00	1.07E-01	29	9.90E-01	9.12E-01	1.68E-01	
		2	55	1.03E+00	1.01E+00	1.24E-01	29	1.00E+00	9.66E-01	1.26E-01	
		3	55	1.02E+00	1.02E+00	1.54E-01	29	9.59E-01	9.26E-01	1.31E-01	*
**Harmonic**	Amp Mod	1	17	1.02E+00	1.02E+00	9.43E-02	8	9.31E-01	8.98E-01	1.70E-01	
		2	17	1.02E+00	1.00E+00	1.25E-01	8	1.02E+00	9.65E-01	1.08E-01	
		3	17	1.02E+00	1.02E+00	1.79E-01	8	9.11E-01	9.00E-01	5.13E-02	*
**Mixed**	Amp Mod	1	18	1.01E+00	1.03E+00	5.93E-02	9	1.01E+00	9.76E-01	1.36E-01	
		2	18	1.02E+00	1.02E+00	7.42E-02	9	9.52E-01	9.69E-01	9.54E-02	#
		3	18	1.02E+00	1.02E+00	1.13E-01	9	9.13E-01	9.34E-01	7.52E-02	*
**Slide**	Frequency	1	13	1.23E+00	1.07E+00	4.67E-01	5	8.54E-01	8.38E-01	1.73E-01	*
		2	13	1.21E+00	9.39E-01	9.09E-01	5	8.46E-01	7.88E-01	3.16E-01	
		3	13	1.28E+00	9.54E-01	6.73E-01	5	8.79E-01	8.68E-01	1.77E-01	#

* indicates p < 0.05.

^#^ indicates 0.05 < p < 0.1.

#### Asyn overexpression leads to changes in self-similarity of syllables depending on syllable type

To assess the effects of αsyn overexpression on the overall structure of syllables, two composite scores were calculated, percent similarity (%similarity) and accuracy. Percent similarity represents a 50ms sampling of an individual syllable, whereas accuracy represents a single millisecond sampling of the same syllable [55]. Both scores provide a gross (%similarity) or fine grain (Accuracy) resolution of how well that syllable matches its own structure over multiple renditions. We found that the mean accuracy of mixed syllables was significantly lower at two (p = 0.0277, Wilcoxon Rank Sum Test) and three (p = 0.0148, Wilcoxon Rank Sum Test) mpi for the ASYN group compared to GFP controls (S14 Fig). No other significant differences were found for these self-similarity measures (S15 Fig). Additionally, a Welch ANOVA and simple linear model were conducted to compare similarity scores across time points by syllable type, but no differences were observed.

## Discussion

Asyn neuropathology is the primary hallmark for the family of neurodegenerative diseases categorized as synucleinopathies of which PD is the most common [71–74]. Unfortunately, very little is known about the etiopathogenesis of these diseases. In the case of PD, disruptions in axonal trafficking, ER to Golgi transport, synaptic vesicle recycling, neurotransmitter release, Ca^2+^ and mitochondrial homeostasis are implicated during the early stages of this disease [5,71–74]. However, it has been difficult interpreting these results since we lack a reliable behavioral phenotype that coincides with early changes in these biological processes. To address this critical need, we developed a novel genetic model of αsyn overexpression using the highly translational zebra finch songbird. In contrast to mammalian PD models that use neurotoxin or genetic approaches to target dopaminergic neurons in the midbrain, here, we sought to virally target a finch basal ganglia region, Area X, that is specifically dedicated to vocal modulation, an approach not possible in current mammalian models. We hypothesized that overexpression of human wild type αsyn in Area X would decrease the quality of the bird’s song. We predicted that the severity of this deterioration would coincide with αsyn neuropathology in Area X. Our results show that overexpressing human wild-type αsyn within Area X impairs song production and vocal quality of select syllable types. Interestingly, these changes (i.e., less vocalizing, shorter call length, and deteriorating syllable structure) parallel what is seen in some individuals with PD [6–17]. Furthermore, while we targeted Area X with virus, we unexpectedly detected αsyn signal in lMAN.

### Detection of αsyn in the cortical song center lMAN highlights possible role of this region in Parkinsonian vocal deterioration

Following overexpression of αsyn in Area X processes, we also qualitatively detected αsyn signal in a small population of neurons within the cortical song nucleus lMAN from select birds raising the question about the role of this region in contributing to Parkinsonian-like vocal changes in our model. Area X receives extensive glutamatergic input from lMAN in the AFP (Fig 1). This pathway’s role, therefore, is considered critical for modulation of song in adult, male zebra finches (Fig 1) [reviewed in: 75]. It is unclear, however, what mechanism led to an increase in αsyn signal within lMAN neurons. Because lMAN innervates Area X, one possible explanation would be that AAV5-SNCA is endocytosed by lMAN presynaptic terminals in Area X and retrogradely trafficked to cell bodies in lMAN for transcription. Previous reports in rats suggest that retrograde transport of AAVs, including AAV5, from processes to cell body can occur [76], however, it is unclear whether this happens in our PD finch model. One possible experiment to determine whether the virus is being trafficked to lMAN cell bodies would be to conduct *in situ* hybridization or q-rtPCR experiments in future studies. Elevated levels of *SNCA* gene would indicate that the virus is being retrogradely trafficked in our finch PD model. Alternatively, human and rodent PD studies show that abnormal spread of αsyn pathology from mid and hindbrain regions to cortex can also occur [63,71,77]. It is unclear, however, whether this prion-like spread of αsyn pathology is occurring in our Parkinsonian finch model. Future studies should explore whether this process can occur in finches as well by targeting Area X with an AAV encoding for SNCA with a tag such as myc [78]. This would allow for targeting of virally-encoded protein with a myc antibody, thereby allowing tracking of αsyn pathology in distant song-dedicated regions by immunohistochemically targeting the myc tag. Once this is established, one could assess whether this PD pathology scales with vocal deterioration over a longer time course (beyond three months in our current study) as is predicted in PD (i.e., progressive effects of αsyn pathology on vocal quality and production).

The cell-type specific mechanisms whereby αsyn overexpression disrupts lMAN input onto Area X and birdsong are not known. Previous reports in rodent models suggest that loss of glutamatergic input augments medium spiny neuron activity [79,80]. Medium spiny neurons are the most abundant cell type in the basal ganglia of finches, including in Area X [75,81]. Alternatively, vocal changes could be related to altered signaling between lMAN and the robust nucleus of the arcopallium (RA). Importantly, lMAN is the main output center of this pathway, connecting to RA via an excitatory synapse. The RA exerts control over the tracheosyringeal cranial nerve in the brainstem: lesions of the lMAN to RA synapse result in impaired song modulation [82; also reviewed in: 47]. Therefore, impairment of this lMAN to RA connection could explain why αsyn overexpressing birds produce less song relative to control finches at precise time points. Future experiments will explore this critical connection using electrophysiological approaches to record from lMAN and Area X and relate deterioration in activity from specific cell-types within both regions to impaired song features. This would take advantage of the finch song system, wherein multiple cell types are clustered into one area.

### Presence of insoluble αsyn monomers is a hallmark of early stage Parkinson’s disease

A previous report using the highly characterized Thy-1 transgenic mouse model of PD characterized by human αsyn overexpression suggested that vocal deficits coincide with αsyn aggregation in the brain of mice including the basal ganglia at two to three months of age [27]. Gombash et al [26] reported reduced call intensity and rate in rats eight weeks following targeted injection of their AAV construct overexpressing human wild-type αsyn into substantia nigra with aggregates reported in cell bodies and neurites in striatum. Whereas these studies used transgenic or viral-mediated methods to elevate levels of αsyn in the brain, Purmier et al caused rapid onset αsyn neuropathology by seeding the striatum of their rats with preformed fibrils [28]. One advantage of this rapid onset approach is that it can lead to similarly rapid behavioral changes (i.e., deterioration of vocal quality and production). In rats, this translated to reduced calling, impaired call duration/rate, and lower peak frequency. Here, we take advantage of the AAV system to target overexpression of human wild-type αsyn within a specific song nucleus and evaluate the direct relationship between pathology in the brain and behavior. Interestingly, our finch model of αsyn overexpression shows similar vocal findings to these various rodent models despite differences in neural circuits for control of vocalization, timescales, and targeting of virus injection. Our findings in Area X indicate that elevated levels of monomeric insoluble αsyn protein coincide with changes in song production and quality. Additionally, at the time points examined, we did not detect significant elevation in higher molecular weight species (e.g. 50kD and multimer) of αsyn in Area X from our Parkinsonian finches despite these robust and subtle song changes. These high molecular species have previously been linked to formation of aggregates that mark the later stages of the disease [3,4,28,61–66,71]. Here, the increase in insoluble αsyn monomer within Area X underscores these initial early vocal changes. Furthermore, changes in monomeric protein as a result of AAV-SNCA injection were restricted within the basal ganglia to Area X because no differences were detected in VSP. Levels of monomeric protein were not measured in lMAN. Whether the neuropathology in basal ganglia Area X and cortical lMAN song centers extend to later time points, beyond three months, requires further investigation. Future studies should therefore monitor pathological changes at later time points or increase viral titer to seed more severe αsyn neuropathology. Interestingly, leveraging the rapid onset, pre-formed fibril approach in zebra finch could provide additional insight into the more severe, later stage vocal changes observed in Parkinson’s patients. Additionally, future experiments should measure soluble and insoluble levels of αsyn protein in other song centers to understand the extent of αsyn neuropathology in our zebra finch model and its relationship to vocal changes.

While the results comparing soluble and insoluble monomeric αsyn protein in Area X were straightforward across the ASYN, GFP, and Non-Surgical (NS) groups, findings from the higher molecular weight species were not. The GFP control and ASYN groups showed reduced levels of urea soluble αsyn species at 50kD compared to the NS group, suggesting that viral-mediated transfection might lead to changes in protein homeostasis. This could happen because AAVs including GFP constructs becomes toxic at high levels in other animal models [83–86]. Surprisingly, we also observed changes in soluble and insoluble αsyn within VSP of GFP control, but not ASYN groups. Interestingly, previous studies have shown that these higher molecular weight species exist in a non-pathological form and play a crucial role in regulating neurotransmitter release at the presynaptic terminal [87–90]. Therefore, one possibility is that non-surgically injected finches endogenously produce higher levels of high molecular weight (e.g., 50kD and multimers) αsyn but that viral-driven expression leads to disruption in formation of these species thus driving these down in both Area X and VSP. It is unclear why these changes were not observed in the VSP of the ASYN group, but the variability in the results (i.e., size of box and whisker plots) from the ASYN birds suggests that additional factors are contributing to their response. Additional controls using scrambled vector designs and sham injected animals should be implemented in future investigations alongside the tagged human *SNCA* gene to uncover these mechanisms.

### Overexpression of αsyn in Area X causes distinct vocalization deficits depending on syllable type

Individuals with early to late-stage PD present with vocal symptoms such as reduced length of mean utterance and quieter, more monotonous, more monopitch, and hoarser voice [6–17]. Here, we report that αsyn overexpressing birds sing less two months after virus injection and sing less at the start of a song session three months after virus injection compared to pre-injection. These findings suggest that vocal fatigue corresponds to decreased motivation or effort to start singing rather than fatigue caused from excessive vocal exercise since decreased singing in the ASYN finches is observed at the beginning of the song session (e.g. first 30 minutes). Our results showing that there is a decrease in how much our Parkinsonian birds sing during the first 30 minutes of the morning song session and total two-hour song session suggest that Parkinsonian birds have difficulty singing at the start of the day. This finding contrasts with what is observed typically in birds, where singing peaks at the start of the day and decreases as the day progresses [69,70]. Taken together, these findings reflect PD patients’ difficulties with initiating speech and the development of vocal fatigue [17,67,68]. Future experiments should explore whether biomarkers related to motivation such as dopamine are dampened during the early stages in our model resulting in decreased vocal output [91]. In addition to changes in vocal production, we measured the individual acoustic features of syllables within the bird’s song to understand the effects of αsyn overexpression on vocal quality (see ‘Song recording and analysis’ section, Table 1). Importantly, we interpret var scores in these acoustic features as within-rendition variability and CV scores as the across-rendition variability. Given this, we propose that the CV of amplitude is a proxy for monoloudness (i.e., variability in the loudness of the syllable across renditions), the CV of pitch is a proxy for monopitch (i.e., variability in pitch across renditions), var plus CV of FM are proxies for monopitch at peak frequencies, var plus CV of AM are proxies for monoloudness, and, lastly, var plus CV of Wiener entropy suggest that a syllable is becoming noisier or purer (i.e., change to structure of syllable). Therefore, in line with the human literature and our interpretation of the acoustic features scores, our αsyn overexpression finch model exhibits shorter vocalizations and a quieter song depending on type of syllable. Harmonic syllables, which are similar to human vowels [59] had pronounced changes in amplitude modulation and their entropy scores were more variable relative to controls, suggesting that the structure of these syllables is harder to maintain following αsyn overexpression. Mixed syllables, that have a noisier component and can be considered consonant-like, were shorter in duration, showed changes in amplitude modulation, and impaired rendition-to-rendition accuracy relative to controls. Reports of consonant and vowel specific changes have previously been reported in human PD studies [92–95], however, it has been difficult to study the neurobiology of this phenomena in animal models. Table 8 summarizes comparisons between our finch PD model and human PD. Our findings suggest that the cortico-basal ganglia-thalamo-cortico circuit for vocal modulation differentially regulate syllables depending on type. Interestingly, our analyses did not reveal that these syllable-dependent changes in acoustic features and similarity scores worsen over time. This finding contrasts with what is observed in human PD patients and other animal models of PD, where vocal changes progressively deteriorate as the disease spreads throughout the brain [13,17]. One explanation is that we only examined vocal changes over three months and that this time course was too short to detect progressive changes. Future experiments should be conducted over a longer time course, so vocal changes can be followed and then related to extent of αsyn pathology.

**Table 8:** Relationship between changes in zebra finch song and human speech/voice deficits in PD. This table compares significant findings from our finch PD model to the perceptual analogs in humans with PD. In humans, studies generally focus on changes in pitch, loudness, voice quality, and rate of speech with individual differences reported across subjects (see references on specific acoustic characteristics and measurements) [6–17,30,67,68,92–98]. Meanwhile, studies in zebra finches focus on the individual acoustic features of syllables in their birdsong, similarity of syllables within and across song syllable renditions, and the amount of song produced [38,39,42,44,47,50–56,58–60,69,70,82].

Fig	Zebra finch PD song changes	Syllable Type	Human speech and voice deficits in PD
6	↓ Singing	Overall	Decreased speaking
7	↓ Syllable Duration	All, Mixed	Short rushes of speech
	↑ Mean Amp Mod	All, Harmonic, Mixed	Variable loudness while speaking
	↓ Pitch Goodness	Slide	Weaker voice
8	↓ CV of Amplitude	All, Noisy, Slide	Monoloudness
	↑ CV in Pitch Goodness	All, Mixed	Variable voice quality
	↑ CV in Wiener entropy	Harmonic	Variable voice quality
	↑ CV in Pitch	Slide	Variable pitch control
	↓ CV of Freq Mod	Slide	Monopitch
9	↑ Var in Amp Mod	All, Harmonic, Mixed	Variable loudness while speaking
	↑ Var in Frequency	Slide	Variable pitch

Taken together, our results suggest that αsyn neuropathology in synucleinopathies such as PD could act through the cortico-basal ganglia-thalamo-cortico circuit to impair vocal behavior. However, a potential limitation of our model is that we do not globally overexpress αsyn throughout this circuit; in mid to late stages of human PD, the protein pathology is widespread in the brain [61]. In contrast to rodent Parkinsonian models that rely on viral-mediated expression of *SNCA* in regions such as the substantia nigra and ventral tegmental areas (SN/VTA) [26], which is highly disrupted in PD, we target expression of SNCA to the song-dedicated basal ganglia region Area X. Area X receives input from the SN/VTA, but is easier to target and specifically affects singing, whereas SN/VTA also modulates non-vocal motor behavior (Fig 1). Therefore, our targeted approach is meant to isolate the contribution of αsyn neuropathology within a specific vocal nucleus within the cortico-basal ganglia-thalamo-cortical loop during the onset of impaired vocal production. Specifically, we were interested in the contribution of αsyn neuropathology within the basal ganglia because it is a highly heterogeneous structure that functions as an important relay center for coordination of advanced motor behaviors such as vocal modulation [99–101]. Nonetheless, given that αsyn is highly expressed throughout the brain in PD patients, our model is predicted to have the same vocal outcome as injecting into SN/VTA without the secondary effects of injecting into this region. Future studies should, therefore, elaborate on the contribution of other vocal and non-vocal dedicated areas to worsening vocal quality in PD, including differentiating the effects of αsyn overexpression in Area X versus cortical song nucleus lMAN or the midbrain SN/VTA. To this end, characterizing the electrophysiological properties of these critical song centers in our PD model would elaborate on the underlying neural substrates contributing to impaired vocal motor behavior.

## Conclusions

Our experiments here sought to determine if viral-mediated expression of human wild-type αsyn in the adult song system changes song output. We found that αsyn overexpression within the zebra finch AFP leads to syllable-dependent changes in song and that this coincides with an increase in insoluble, monomeric αsyn protein in a basal ganglia nucleus for vocal control, but not in neighboring areas. These results have implications for neurodegenerative disorders that impair vocal behavior, especially the family of synucleinopathies.

## Supporting information

S1 Fig GFP expression in the anterior forebrain pathway restricted to Area X. **A)** Representative images of gfp (green signal) and PanNeuronal (purple signal) double label taken from a representative GFP bird highlight transfection of neuronal cell bodies within Area X. **B)** Schematic representation highlighting Area X in a coronal slice of zebra finch brain. **C)** Representative images of a gfp (green signal) and PanNeuronal (purple signal) double label taken from a representative GFP bird highlight a lack of AAV transfection in the cortical song center lMAN. **D)** Schematic representation highlighting lMAN in a coronal slice of zebra finch brain. Tissue was collected from a cohort of GFP birds collected at 3 mpi. Images were taken near center of target region on a Leica DMI 6000B wide field fluorescence microscope with a DFC 450 color camera at 40x magnification. Scale bar (bottom right) is 100μm.
(DOCX)

S2 Fig Pre-absorption control of αsyn isolated from zebra finch Area X and mouse basal ganglia (BG). **A)** A Western blot labelled with primary αsyn antibody preabsorbed using αsyn fusion protein (ag1285, Proteintech). **B)** A Western blot labelled with non-preabsorbed primary αsyn antibody. Asyn protein signal is strongly diminished between 15-20kD and 40–250+ kDs. GAPDH protein signal demonstrates proper loading of Western blot. Samples loaded into each blot were obtained from zebra finch Area X and wild-type mouse basal ganglia. Zebra finch samples were collected following two hours of undirected singing. Mouse samples were collected under an unknown vocal state. Samples were then processed either in RIPA lysis buffer (R), low salt buffer (LS), or Urea buffer (U) prior to loading onto SDS-PAGE gel and subsequently transferred to PVDF membrane. Reference Fig 5‘s legend for additional Western Blot details.
(DOCX)

S3 Fig Western Blot comparing αsyn expression levels between wild type (WT) and Thy1-*SNCA* forebrains indicate that the primary αsyn antibody detects overexpression of human αsyn. Asyn protein signal between 15-20kD and 40–250+ kDs is lower in WT than in Thy1-SNCA. Mouse samples from forebrain were collected under an unknown vocal state. Reference Fig 5 and S2’s legend for additional Western Blot details.
(DOCX)

S4 Fig A representative Western blot loaded with low salt (LS) or urea (U) soluble fractions obtained from Area X of non-surgical birds (NS). High and low molecular weight species (50kD+) detected for LS and U soluble αsyn protein in Area X. Low levels of monomeric (15kD) αsyn protein were detected across the NS group, whereas levels of higher molecular weight αsyn protein (50, 100, and 150 kD) are qualitatively higher in Area X. Quantification of relevant αsyn levels for these birds is provided in Table 2 and additional Western Blot details are included in Fig 5‘s legend. VSP blot data from NS group is not shown.
(DOCX)

S5 Fig Asyn expression levels by molecular weight in VSP. **A)** A representative Western blot loaded with low salt (LS) or urea (U) soluble fractions obtained from VSP of birds that received either AAV5-CBA-eGFP or AAV5-CBA-ASYN into Area X. **B)** Western blot loaded with low salt (LS) or urea (U) soluble fractions obtained from VSP of nonsurgical (NS) birds. Western blots were labelled with an αsyn antibody for quantification of this protein’s levels in Area X relative to GAPDH from LS lane of the same sample. **C)** Quantification of blots. Levels of trimeric (~45-50kD) αsyn protein in U fractions are lower in the GFP than in the NS group. Additionally, total levels of αsyn were also lower across LS and U fractions in GFP compared to NS. Levels of multimeric αsyn (75-250kD) in LS and U fractions were also lower in GFP compared to NS. Summary statistics provided in S1 Table. Importantly, for all molecular weights, αsyn expression is not statistically higher within either LS or U fractions in VSP of ASYN group compared to GFP group. The representative blot contains raw data from birds 1 and 2 of both ASYN and GFP control groups. Reference Fig 5‘s legend for additional Western Blot details. Statistical comparisons were made using a Welch test. * indicate p < 0.05.
(DOCX)

S6 Fig Mean acoustic feature scores for all syllables that are not affected by αsyn overexpression. Remaining acoustic features whose scores are not affected significantly by αsyn overexpression. Reference Fig 7‘s legend for explanation of boxplots. Statistical comparisons were made using a Wilcoxon Rank Sum Test.
(DOCX)

S7 Fig Mean acoustic feature scores for Harmonic, Noisy, Mixed, and Slide syllables that are not affected by αsyn overexpression. Remaining acoustic features whose scores arenot affected significantly within syllable types by αsyn overexpression. Reference Fig 7‘s legend for explanation of boxplots. Statistical comparisons were made using a Wilcoxon Rank Sum Test.
(DOCX)

S8 Fig Asyn overexpression shortens duration of not-flat harmonic syllables. The adjusted value of individual acoustic features is plotted for flat harmonic (FlatHarmonic) and non-flat harmonic (NotFlatHarmonics) syllables sung by ASYN and GFP expressing groups. The individual acoustic features of flat harmonic syllables did not statistically differ in the ASYN group (N = 9) compared to GFP control (N = 7). The duration of non-flat harmonic syllables (NotFlatHarmonic) was shorter in the ASYN group (N = 46) compared to GFP control (N = 22) at 1, 2, and 3 mpi. Summary statistics provided in S2 Table. Reference Fig 7‘s legend for explanation of boxplots. Statistical comparisons were made using a Wilcoxon Rank Sum Test. * indicates p < 0.05.
(DOCX)

S9 Fig Across rendition variability of acoustic features for all syllables that are not affected by αsyn overexpression. Remaining acoustic features whose across rendition variability score (CV) are not affected significantly by αsyn overexpression. Reference Fig 7‘s legend for explanation of boxplots. Statistical comparisons were made using a Wilcoxon Rank Sum Test.
(DOCX)

S10 Fig Across rendition variability of acoustic features for Harmonic, Noisy, Mixed, and Slides syllables that are not affected by αsyn overexpression. Remaining acoustic features whose across rendition variability score are not affected significantly within syllable types by αsyn overexpression. Reference Fig 7‘s legend for explanation of boxplots. Statistical comparisons were made using a Wilcoxon Rank Sum Test.
(DOCX)

S11 Fig Asyn overexpression differentially affects across-rendition variability in select individual acoustic features depending on whether it is a flat harmonic. The adjusted value of across rendition variation (i.e., CV) in acoustic features is plotted for flat harmonic (FlatHarmonic) and non-flat harmonic (NotFlatHarmonics) types sung by ASYN and GFP expressing groups. The CV of entropy (mean.entropy) for flat harmonic syllables was higher in the ASYN group (N = 9) compared to GFP control (N = 7) at 1, 2, and 3 mpi. Whereas, the CV of amplitude (mean.amplitude.adjusted) of the non-flat harmonic syllables (NotFlatHarmonics) was lower in the ASYN group (N = 46) compared to GFP control (N = 22) at 2 mpi with a trend detected at 3 mpi. Additionally, the CV of pitch goodness for these non-flat harmonic syllables was also higher in the ASYN group compared to GFP control at 3 mpi. Summary statistics provided in S2 Table. Reference Fig 7‘s legend for explanation of boxplots. Statistical comparisons were made using a Wilcoxon Rank Sum Test. * indicates p < 0.05. # indicates 0.05 < p < 0.1.
(DOCX)

S12 Fig Within rendition variability of acoustic features for all, Harmonic, Noisy, Mixed, and Slide syllables that are not affected by αsyn overexpression. Remaining acoustic features whose within rendition variability score are not affected significantly within syllable types by αsyn overexpression. Reference Fig 7‘s legend for explanation of boxplots. Statistical comparisons were made using a Wilcoxon Rank Sum Test.
(DOCX)

S13 Fig Asyn overexpression leads to monopitch of non-flat harmonic syllables. The adjusted value of within rendition variation (i.e., var) in acoustic features is plotted for flat harmonic (FlatHarmonic) and non-flat harmonic (NotFlatHarmonics) syllables sung by ASYN and GFP expressing groups. No effects were detected for variance of individual acoustic features in the flat harmonic syllables, when we compared the ASYN group (N = 9) to GFP control (N = 7). However, the variance of frequency modulation (var.FM) for non-flat harmonic syllables (NotFlatHarmonics) was lower in the ASYN group compared to GFP control at 2 mpi with a trend detected at 3 mpi. Summary statistics provided in S2 Table. Reference Fig 7‘s legend for explanation of boxplots. Statistical comparisons were made using a Wilcoxon Rank Sum Test. * indicates p < 0.05. # indicates 0.05 < p < 0.1.
(DOCX)

S14 Fig Asyn overexpression affects the self-accuracy score of mixed syllables. The adjusted value of self-similarity scores (%Similarity and Accuracy) is plotted for All Syllables, then grouped by Harmonic, Mixed, Noisy, and Slide syllables sung by ASYN and GFP expressing groups. The accuracy score of mixed syllables was lower in the ASYN group (N = 18) compared to GFP control (N = 9) at 2 and 3 mpi. Summary statistics provided in S3 Table. Reference Fig 7‘s legend for explanation of boxplots. Statistical comparisons were made using a Wilcoxon Rank Sum Test. * indicates p < 0.05.
(DOCX)

S15 Fig Asyn overexpression does not affect similarity scores of flat or non-flat harmonic syllables. The adjusted value of self-similarity scores (%Similarity and Accuracy) is plotted for flat harmonic (FlatHarmonic) and non-flat harmonic (NotFlarHarmonic) syllables sung by ASYN or GFP expressing groups. No effects were found for the %Similary or Accuracy of either the flat harmonics (FlatHarmonics; N_ASYN_ = 9; N_GFP_ = 7) or non-flat harmonics (NotFlatHarmonic; N_ASYN_ = 46; N_GFP_ = 22). Summary statistics provided in S2 Table. Reference Fig 7‘s legend for explanation of boxplots. Statistical comparisons were made using a Wilcoxon Rank Sum Test.
(DOCX)

S1 Table Summary statistics of normalized soluble and insoluble αsyn levels in VSP grouped by condition and molecular weight (Mol. Wt.). Conditions and molecular weights correspond to values referenced in S5 Fig.
(DOCX)

S2 Table Summary statistics of mean, across- and within-rendition variability scores grouped by Flat Harmonic type and experimental condition. Reference Table 5‘s legend for additional variable names. * indicates p < 0.05. # indicates 0.05 < p < 0.1.
(DOCX)

S3 Table Summary statistics of Mixed syllable accuracy scores grouped by experimental condition. * indicates p < 0.05. # indicates 0.05 < p < 0.1.
(DOCX)

S1 Raw images Raw images for all blots used in the manuscript are included. (PDF)
