# Supplementary material for: Vocal changes in a zebra finch model of Parkinson’s disease characterized by alpha-synuclein overexpression in the song-dedicated anterior forebrain pathway
Source: PLoS One. 2022 May 4;17(5):e0265604. doi: 10.1371/journal.pone.0265604 (PMC9067653; doi:10.1371/journal.pone.0265604)
Supplement: S1 Fig — A) Representative images of gfp (green signal) and PanNeuronal (purple signal) double label taken from a representative GFP bird highlight transfection of neuronal cell bodies within Area X. B) Schematic representation highlighting Area X in a coronal slice of zebra finch brain. C) Representative images of a gfp (green signal) and PanNeuronal (purple signal) double label taken from a representative GFP bird highlight a lack of AAV transfection in the cortical song center lMAN. D) Schematic representation highlighting lMAN in a coronal slice of zebra finch brain. Tissue was collected from a cohort of GFP birds collected at 3 mpi. Images were taken near center of target region on a Leica DMI 6000B wide field fluorescence microscope with a DFC 450 color camera at 40x magnification. Scale bar (bottom right) is 100μm. (DOCX) [file pone.0265604.s001.docx]

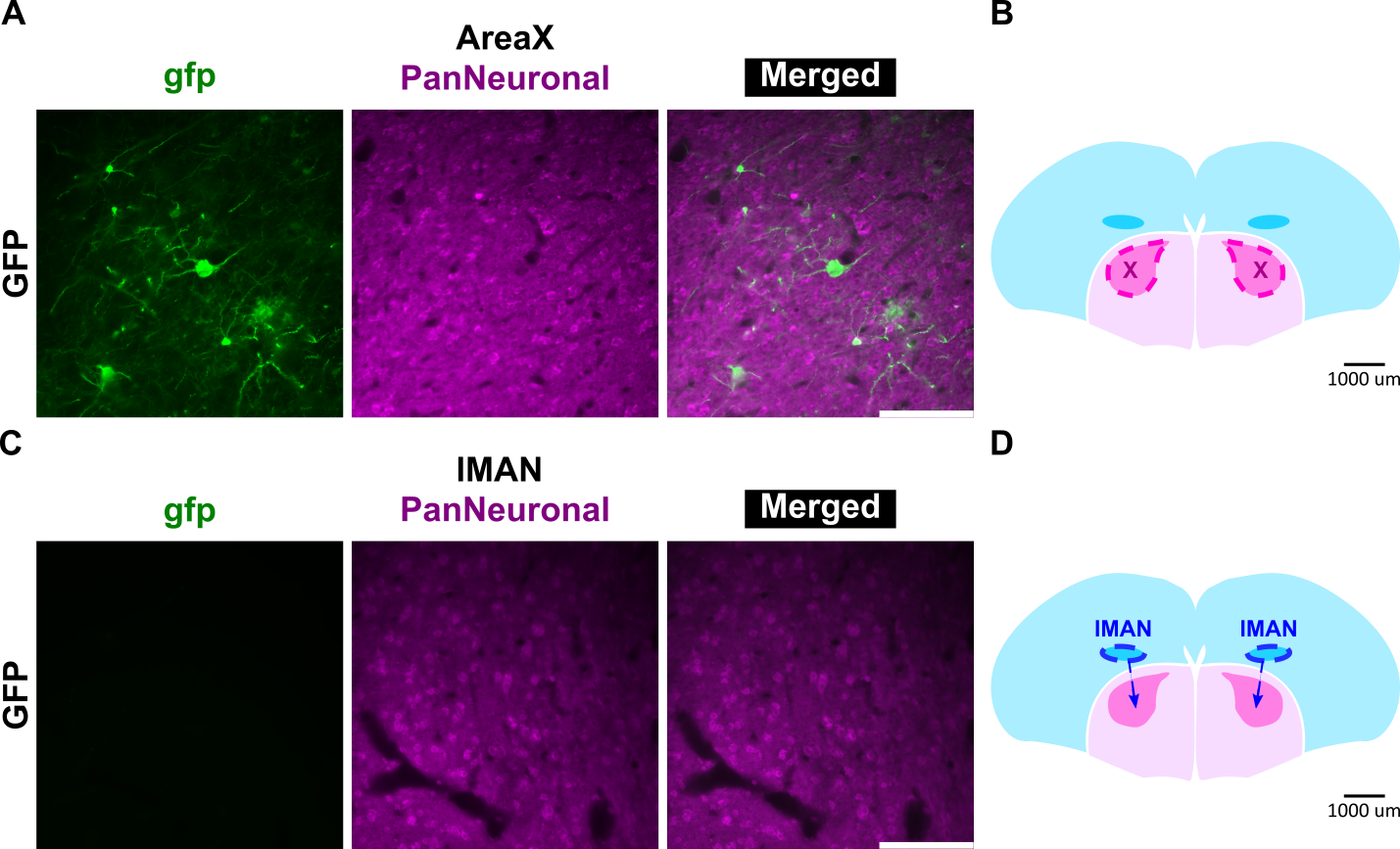


**S1. GFP expression in the anterior forebrain pathway restricted to Area X.** **A)** Representative images of gfp (green signal) and PanNeuronal (purple signal) double label taken from a representative GFP bird highlight transfection of neuronal cell bodies within Area X. **B)** Schematic representation highlighting Area X in a coronal slice of zebra finch brain. **C)** Representative images of a gfp (green signal) and PanNeuronal (purple signal) double label taken from a representative GFP bird highlight a lack of AAV transfection in the cortical song center lMAN. **D)** Schematic representation highlighting lMAN in a coronal slice of zebra finch brain. Tissue was collected from a cohort of GFP birds collected at 3 mpi. Images were taken near center of target region on a Leica DMI 6000B wide field fluorescence microscope with a DFC 450 color camera at 40x magnification. Scale bar (bottom right) is 100µm.
