# Supplementary material for: Vocal changes in a zebra finch model of Parkinson’s disease characterized by alpha-synuclein overexpression in the song-dedicated anterior forebrain pathway
Source: PLoS One. 2022 May 4;17(5):e0265604. doi: 10.1371/journal.pone.0265604 (PMC9067653; doi:10.1371/journal.pone.0265604)
Supplement: S2 Fig — A) A Western blot labelled with primary αsyn antibody preabsorbed using αsyn fusion protein (ag1285, Proteintech). B) A Western blot labelled with non-preabsorbed primary αsyn antibody. Asyn protein signal is strongly diminished between 15-20kD and 40–250+ kDs. GAPDH protein signal demonstrates proper loading of Western blot. Samples loaded into each blot were obtained from zebra finch Area X and wild-type mouse basal ganglia. Zebra finch samples were collected following two hours of undirected singing. Mouse samples were collected under an unknown vocal state. Samples were then processed either in RIPA lysis buffer (R), low salt buffer (LS), or Urea buffer (U) prior to loading onto SDS-PAGE gel and subsequently transferred to PVDF membrane. Reference Fig 5‘s legend for additional Western Blot details. (DOCX) [file pone.0265604.s002.docx]

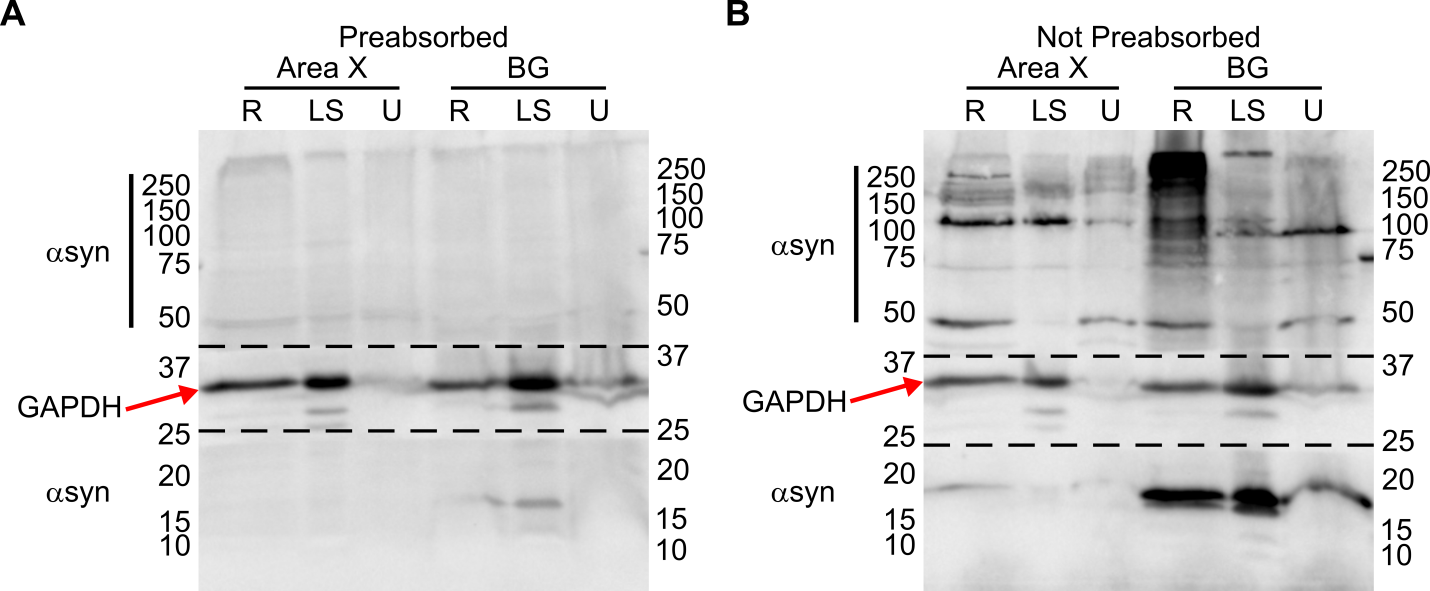


**S2.** **Pre-absorption control of αsyn** **isolated from zebra finch Area X and mouse basal ganglia (BG).** **A)** A Western blot labelled with primary αsyn antibody preabsorbed using αsyn fusion protein (ag1285, Proteintech). **B)** A Western blot labelled with non-preabsorbed primary αsyn antibody. Asyn protein signal is strongly diminished between 15-20kD and 40-250+ kDs. GAPDH protein signal demonstrates proper loading of Western blot. Samples loaded into each blot were obtained from zebra finch Area X and wild-type mouse basal ganglia. Zebra finch samples were collected following two hours of undirected singing. Mouse samples were collected under an unknown vocal state. Samples were then processed either in RIPA lysis buffer (R), low salt buffer (LS), or Urea buffer (U) prior to loading onto SDS-PAGE gel and subsequently transferred to PVDF membrane. Reference Fig 5’s legend for additional Western Blot details.
