# Supplementary material for: Vocal changes in a zebra finch model of Parkinson’s disease characterized by alpha-synuclein overexpression in the song-dedicated anterior forebrain pathway
Source: PLoS One. 2022 May 4;17(5):e0265604. doi: 10.1371/journal.pone.0265604 (PMC9067653; doi:10.1371/journal.pone.0265604)
Supplement: S3 Fig — Asyn protein signal between 15-20kD and 40–250+ kDs is lower in WT than in Thy1-SNCA. Mouse samples from forebrain were collected under an unknown vocal state. Reference Fig 5 and S2’s legend for additional Western Blot details. (DOCX) [file pone.0265604.s003.docx]

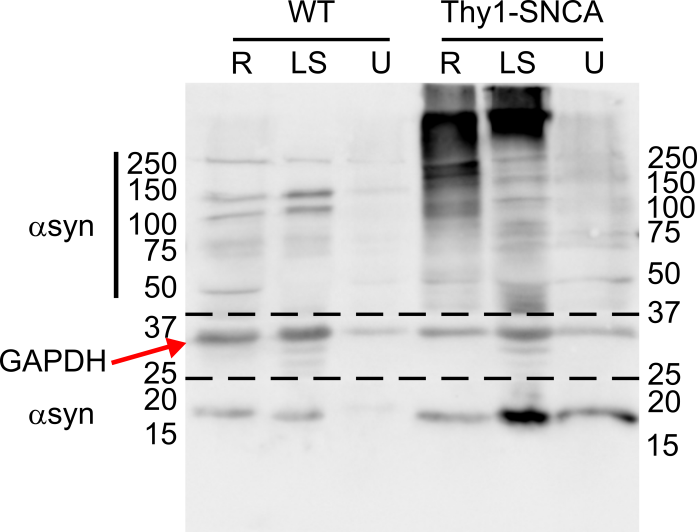


**S3.** **Western Blot comparing αsyn expression levels between wild type (WT) and Thy1-*SNCA* forebrains indicate that the primary αsyn antibody detects overexpression of human αsyn.** Asyn protein signal between 15-20kD and 40-250+ kDs is lower in WT than in Thy1-SNCA. Mouse samples from forebrain were collected under an unknown vocal state. Reference Fig 5 and S2’s legend for additional Western Blot details.
