# Supplementary material for: Vocal changes in a zebra finch model of Parkinson’s disease characterized by alpha-synuclein overexpression in the song-dedicated anterior forebrain pathway
Source: PLoS One. 2022 May 4;17(5):e0265604. doi: 10.1371/journal.pone.0265604 (PMC9067653; doi:10.1371/journal.pone.0265604)
Supplement: S4 Fig — High and low molecular weight species (50kD+) detected for LS and U soluble αsyn protein in Area X. Low levels of monomeric (15kD) αsyn protein were detected across the NS group, whereas levels of higher molecular weight αsyn protein (50, 100, and 150 kD) are qualitatively higher in Area X. Quantification of relevant αsyn levels for these birds is provided in Table 2 and additional Western Blot details are included in Fig 5‘s legend. VSP blot data from NS group is not shown. (DOCX) [file pone.0265604.s004.docx]

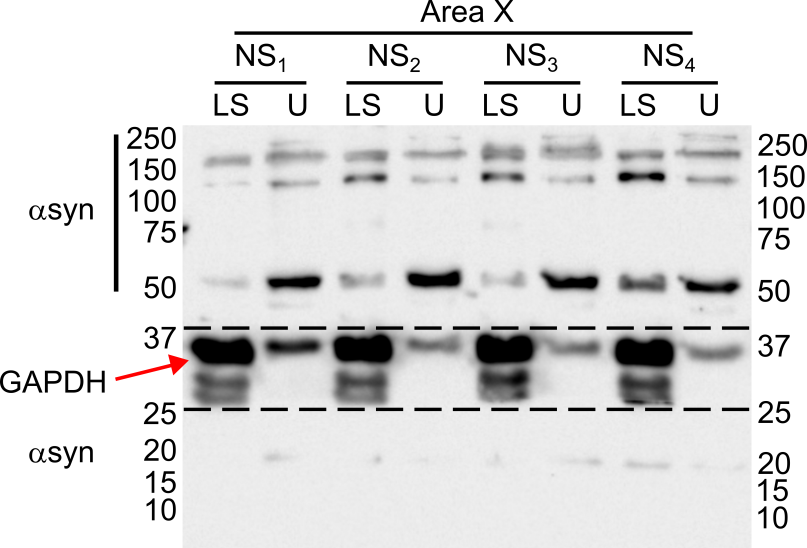


**S4. A representative Western blot loaded with low salt (LS) or urea (U) soluble fractions obtained from Area X of non-surgical birds (NS).** High and low molecular weight species (50kD+) detected for LS and U soluble αsyn protein in Area X. Low levels of monomeric (15kD) αsyn protein were detected across the NS group, whereas levels of higher molecular weight αsyn protein (50, 100, and 150 kD) are qualitatively higher in Area X. Quantification of relevant αsyn levels for these birds is provided in Table 2 and additional Western Blot details are included in Fig 5’s legend. VSP blot data from NS group is not shown.
