# Supplementary material for: Vocal changes in a zebra finch model of Parkinson’s disease characterized by alpha-synuclein overexpression in the song-dedicated anterior forebrain pathway
Source: PLoS One. 2022 May 4;17(5):e0265604. doi: 10.1371/journal.pone.0265604 (PMC9067653; doi:10.1371/journal.pone.0265604)
Supplement: S5 Fig — A) A representative Western blot loaded with low salt (LS) or urea (U) soluble fractions obtained from VSP of birds that received either AAV5-CBA-eGFP or AAV5-CBA-ASYN into Area X. B) Western blot loaded with low salt (LS) or urea (U) soluble fractions obtained from VSP of nonsurgical (NS) birds. Western blots were labelled with an αsyn antibody for quantification of this protein’s levels in Area X relative to GAPDH from LS lane of the same sample. C) Quantification of blots. Levels of trimeric (~45-50kD) αsyn protein in U fractions are lower in the GFP than in the NS group. Additionally, total levels of αsyn were also lower across LS and U fractions in GFP compared to NS. Levels of multimeric αsyn (75-250kD) in LS and U fractions were also lower in GFP compared to NS. Summary statistics provided in S1 Table. Importantly, for all molecular weights, αsyn expression is not statistically higher within either LS or U fractions in VSP of ASYN group compared to GFP group. The representative blot contains raw data from birds 1 and 2 of both ASYN and GFP control groups. Reference Fig 5‘s legend for additional Western Blot details. Statistical comparisons were made using a Welch test. * indicate p < 0.05. (DOCX) [file pone.0265604.s005.docx]

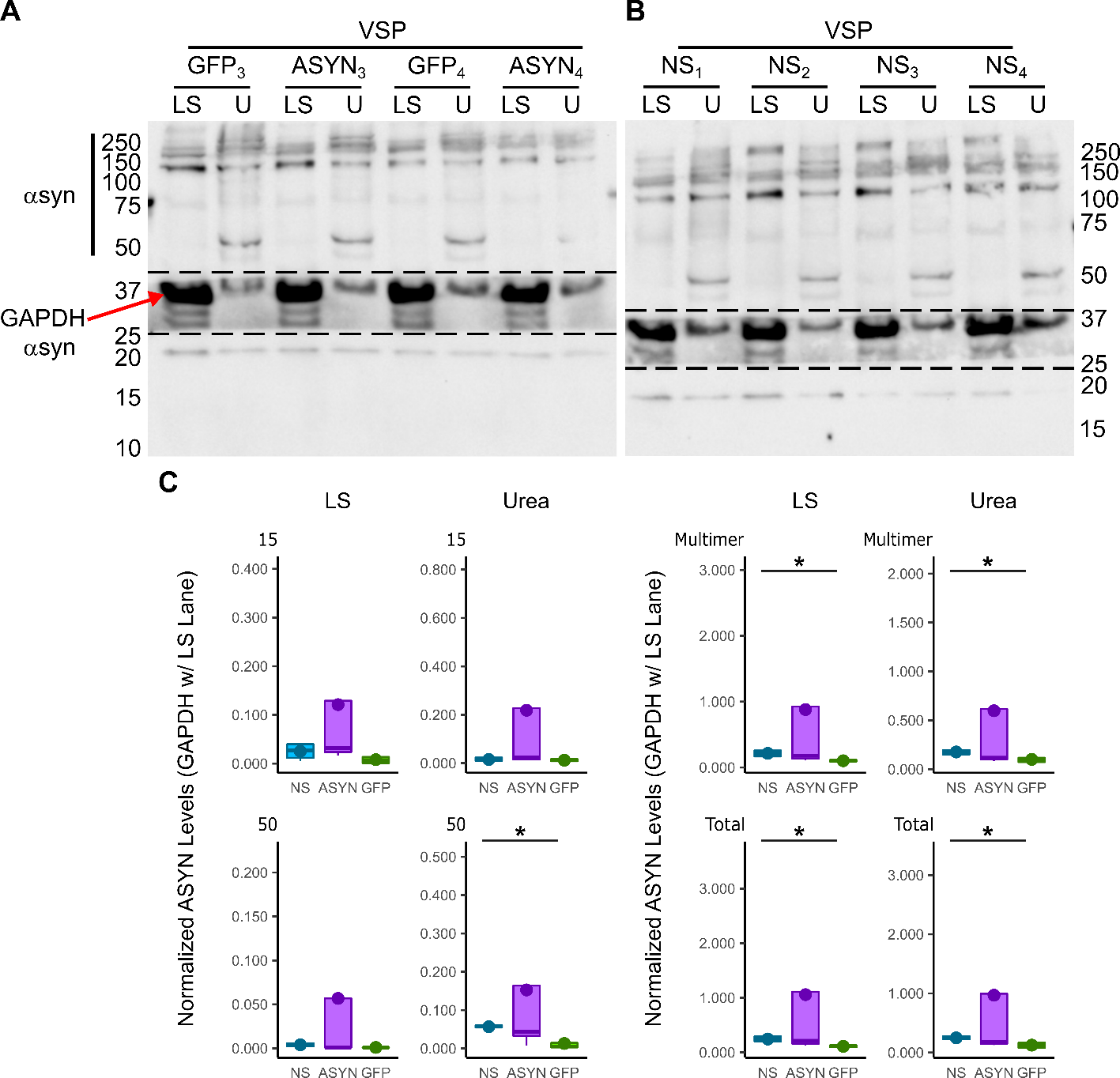


**S5. Asyn expression levels by molecular weight in VSP.** **A)** A representative Western blot loaded with low salt (LS) or urea (U) soluble fractions obtained from VSP of birds that received either AAV5-CBA-eGFP or AAV5-CBA-ASYN into Area X. **B)** Western blot loaded with low salt (LS) or urea (U) soluble fractions obtained from VSP of nonsurgical (NS) birds. Western blots were labelled with an αsyn antibody for quantification of this protein’s levels in Area X relative to GAPDH from LS lane of the same sample. **C)** Quantification of blots. Levels of trimeric (~45-50kD) αsyn protein in U fractions are lower in the GFP than in the NS group. Additionally, total levels of αsyn were also lower across LS and U fractions in GFP compared to NS. Levels of multimeric αsyn (75-250kD) in LS and U fractions were also lower in GFP compared to NS. Summary statistics provided in S1 Table. Importantly, for all molecular weights, αsyn expression is not statistically higher within either LS or U fractions in VSP of ASYN group compared to GFP group. The representative blot contains raw data from birds 1 and 2 of both ASYN and GFP control groups. Reference Fig 5’s legend for additional Western Blot details. Statistical comparisons were made using a Welch test. * indicate p < 0.05.
