# Supplementary material for: Vocal changes in a zebra finch model of Parkinson’s disease characterized by alpha-synuclein overexpression in the song-dedicated anterior forebrain pathway
Source: PLoS One. 2022 May 4;17(5):e0265604. doi: 10.1371/journal.pone.0265604 (PMC9067653; doi:10.1371/journal.pone.0265604)
Supplement: S8 Fig — The adjusted value of individual acoustic features is plotted for flat harmonic (FlatHarmonic) and non-flat harmonic (NotFlatHarmonics) syllables sung by ASYN and GFP expressing groups. The individual acoustic features of flat harmonic syllables did not statistically differ in the ASYN group (N = 9) compared to GFP control (N = 7). The duration of non-flat harmonic syllables (NotFlatHarmonic) was shorter in the ASYN group (N = 46) compared to GFP control (N = 22) at 1, 2, and 3 mpi. Summary statistics provided in S2 Table. Reference Fig 7‘s legend for explanation of boxplots. Statistical comparisons were made using a Wilcoxon Rank Sum Test. * indicates p < 0.05. (DOCX) [file pone.0265604.s008.docx]

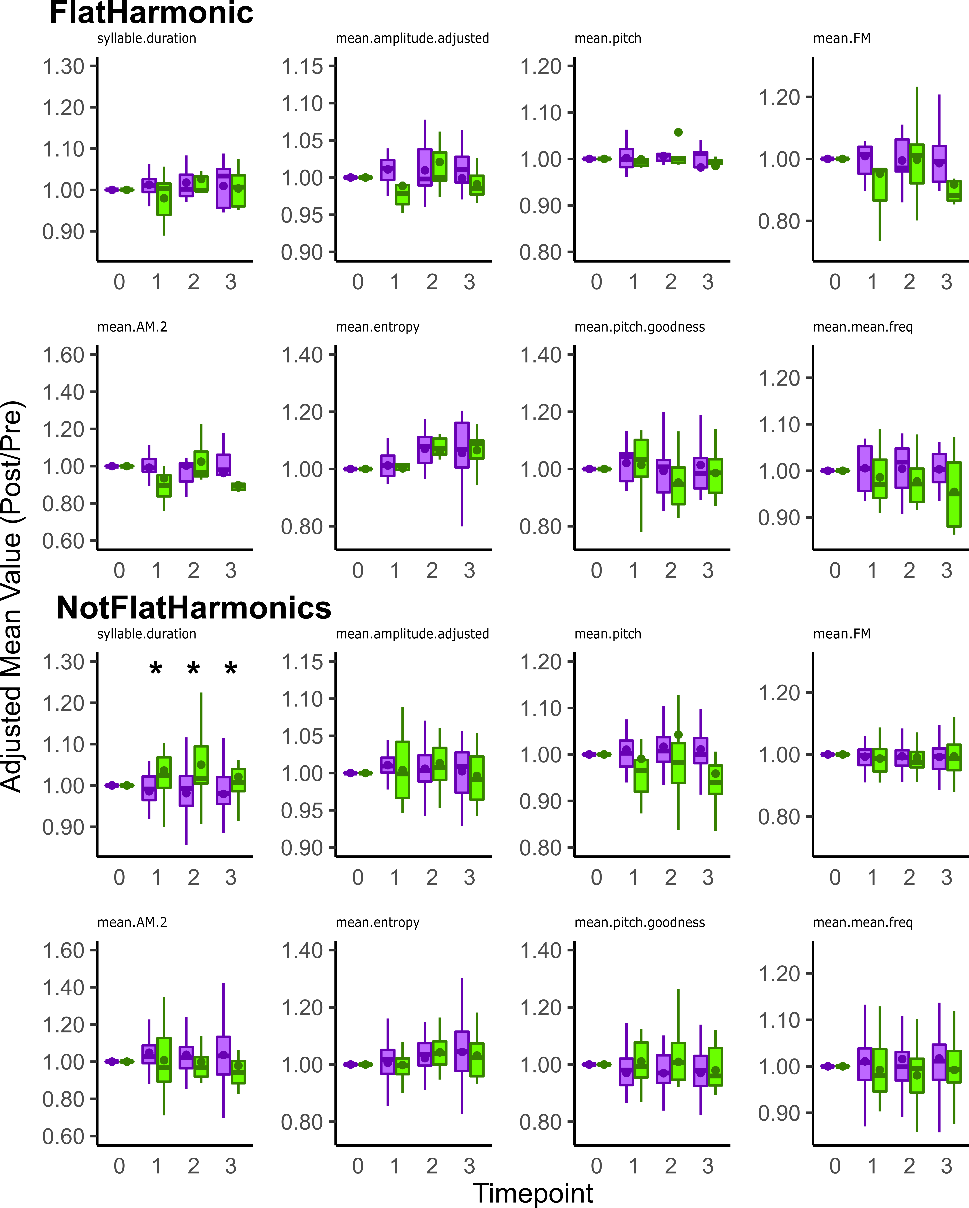


**S8. Asyn overexpression shortens duration of not-flat harmonic syllables.** The adjusted value of individual acoustic features is plotted for flat harmonic (FlatHarmonic) and non-flat harmonic (NotFlatHarmonics) syllables sung by ASYN and GFP expressing groups. The individual acoustic features of flat harmonic syllables did not statistically differ in the ASYN group (N = 9) compared to GFP control (N = 7). The duration of non-flat harmonic syllables (NotFlatHarmonic) was shorter in the ASYN group (N = 46) compared to GFP control (N = 22) at 1, 2, and 3 mpi. Summary statistics provided in S2 Table. Reference Fig 7’s legend for explanation of boxplots. Statistical comparisons were made using a Wilcoxon Rank Sum Test. * indicates p < 0.05.
