# Supplementary material for: Vocal changes in a zebra finch model of Parkinson’s disease characterized by alpha-synuclein overexpression in the song-dedicated anterior forebrain pathway
Source: PLoS One. 2022 May 4;17(5):e0265604. doi: 10.1371/journal.pone.0265604 (PMC9067653; doi:10.1371/journal.pone.0265604)
Supplement: S9 Fig — Remaining acoustic features whose across rendition variability score (CV) are not affected significantly by αsyn overexpression. Reference Fig 7‘s legend for explanation of boxplots. Statistical comparisons were made using a Wilcoxon Rank Sum Test. (DOCX) [file pone.0265604.s009.docx]

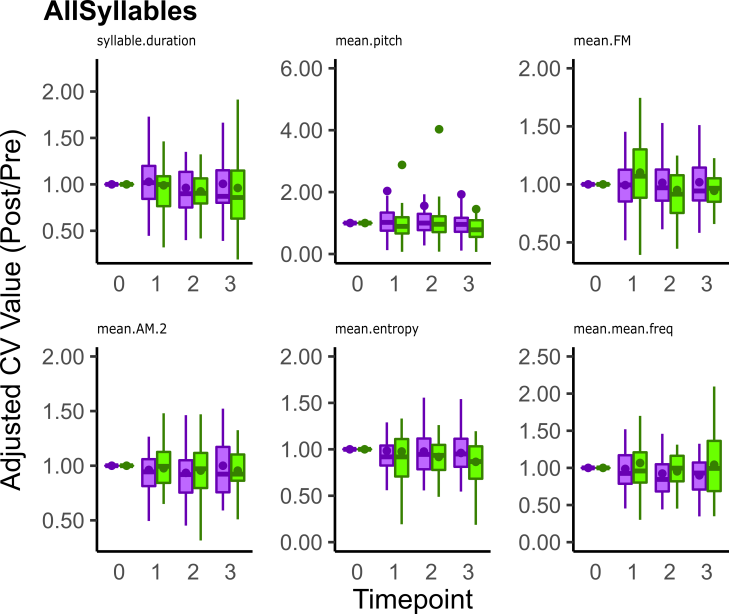


**S9. Across rendition variability of acoustic features for all syllables that are not affected by αsyn overexpression.** Remaining acoustic features whose across rendition variability score (CV) are not affected significantly by αsyn overexpression. Reference Fig 7’s legend for explanation of boxplots. Statistical comparisons were made using a Wilcoxon Rank Sum Test.
