# Supplementary material for: Vocal changes in a zebra finch model of Parkinson’s disease characterized by alpha-synuclein overexpression in the song-dedicated anterior forebrain pathway
Source: PLoS One. 2022 May 4;17(5):e0265604. doi: 10.1371/journal.pone.0265604 (PMC9067653; doi:10.1371/journal.pone.0265604)
Supplement: S10 Fig — Remaining acoustic features whose across rendition variability score are not affected significantly within syllable types by αsyn overexpression. Reference Fig 7‘s legend for explanation of boxplots. Statistical comparisons were made using a Wilcoxon Rank Sum Test. (DOCX) [file pone.0265604.s010.docx]

**
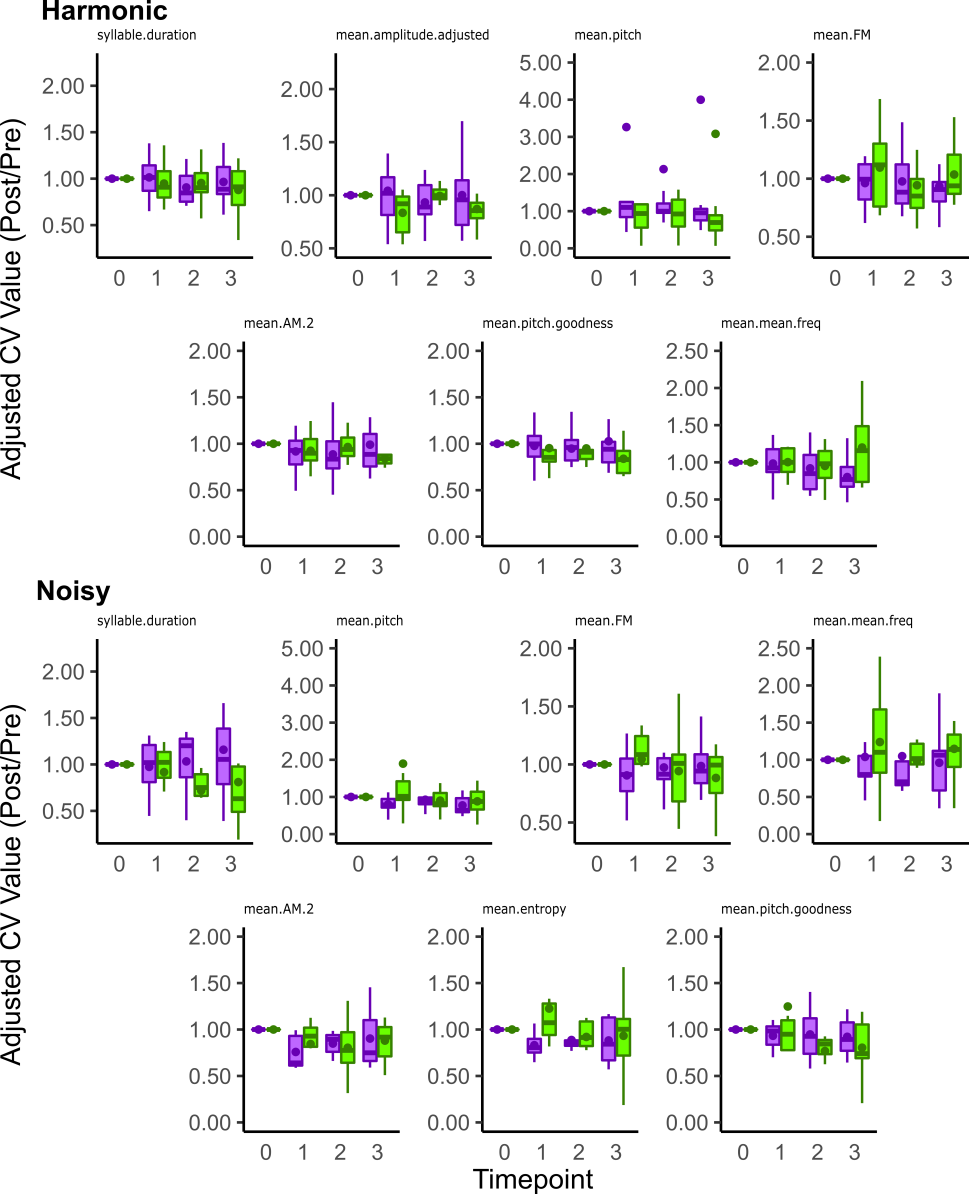
**

**
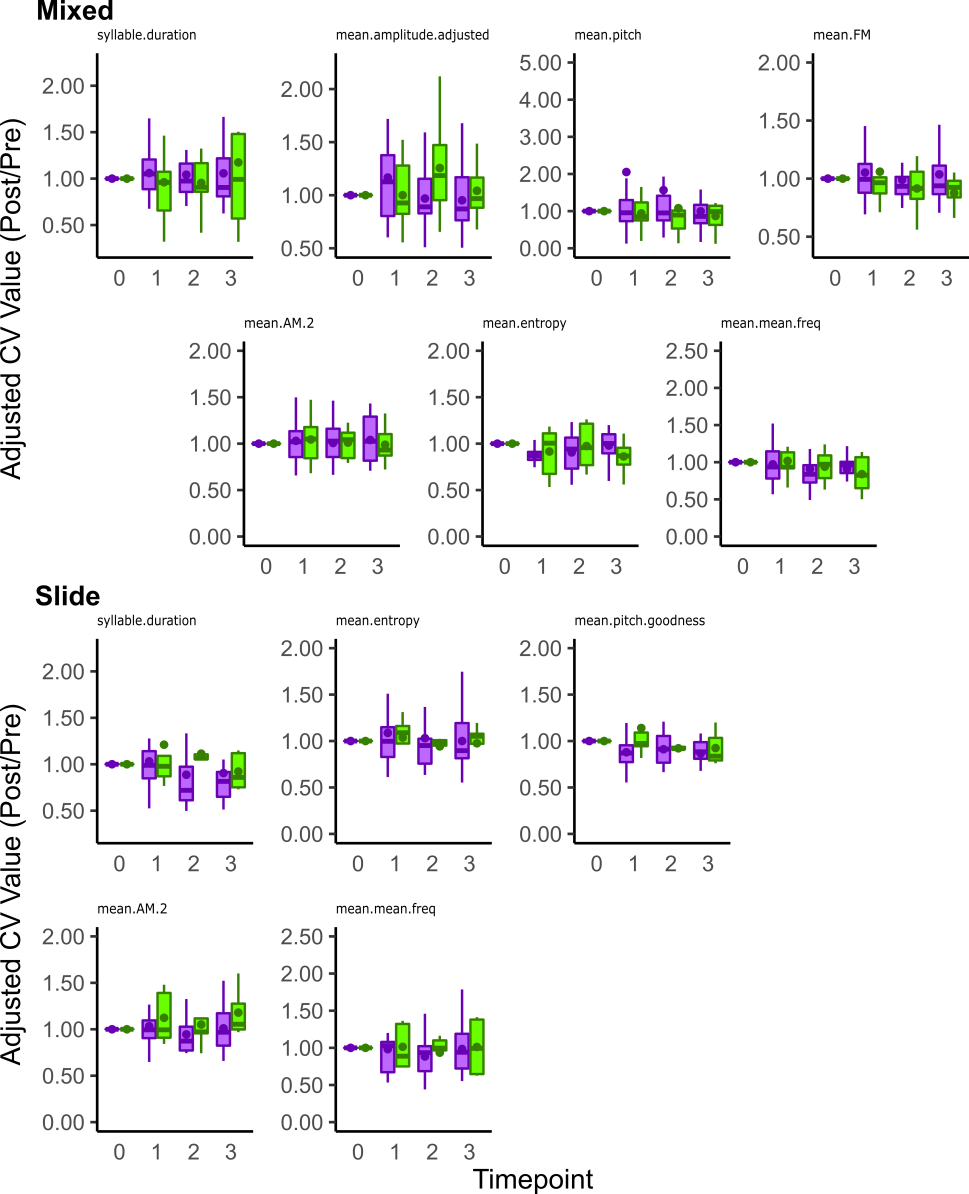
**

**S10. Across rendition variability of acoustic features for Harmonic, Noisy, Mixed, and Slides syllables that are not affected by αsyn overexpression.** Remaining acoustic features whose across rendition variability score are not affected significantly within syllable types by αsyn overexpression. Reference Fig 7’s legend for explanation of boxplots. Statistical comparisons were made using a Wilcoxon Rank Sum Test.
