# Supplementary material for: Vocal changes in a zebra finch model of Parkinson’s disease characterized by alpha-synuclein overexpression in the song-dedicated anterior forebrain pathway
Source: PLoS One. 2022 May 4;17(5):e0265604. doi: 10.1371/journal.pone.0265604 (PMC9067653; doi:10.1371/journal.pone.0265604)
Supplement: S11 Fig — The adjusted value of across rendition variation (i.e., CV) in acoustic features is plotted for flat harmonic (FlatHarmonic) and non-flat harmonic (NotFlatHarmonics) types sung by ASYN and GFP expressing groups. The CV of entropy (mean.entropy) for flat harmonic syllables was higher in the ASYN group (N = 9) compared to GFP control (N = 7) at 1, 2, and 3 mpi. Whereas, the CV of amplitude (mean.amplitude.adjusted) of the non-flat harmonic syllables (NotFlatHarmonics) was lower in the ASYN group (N = 46) compared to GFP control (N = 22) at 2 mpi with a trend detected at 3 mpi. Additionally, the CV of pitch goodness for these non-flat harmonic syllables was also higher in the ASYN group compared to GFP control at 3 mpi. Summary statistics provided in S2 Table. Reference Fig 7‘s legend for explanation of boxplots. Statistical comparisons were made using a Wilcoxon Rank Sum Test. * indicates p < 0.05. # indicates 0.05 < p < 0.1. (DOCX) [file pone.0265604.s011.docx]

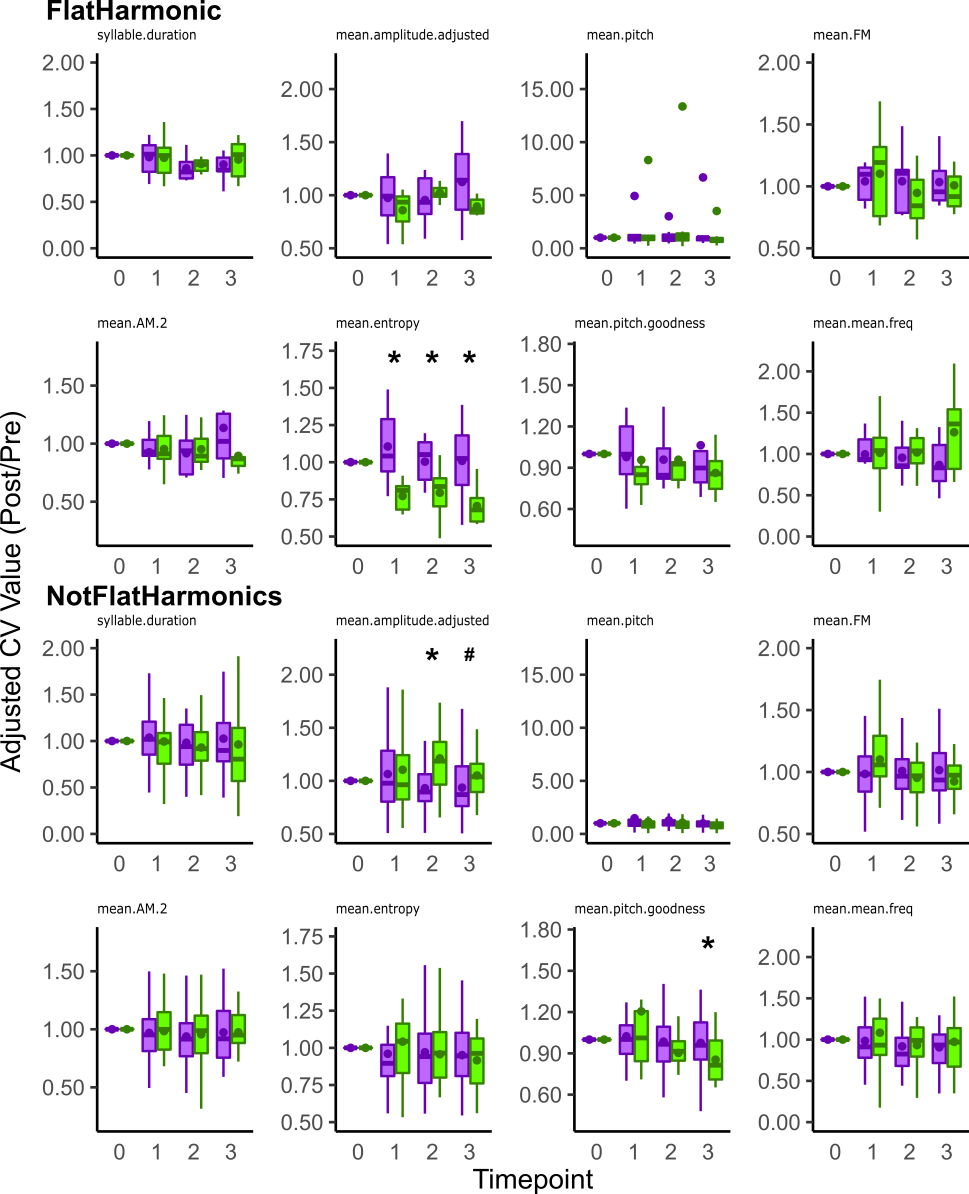


**S11. Asyn overexpression differentially affects across-rendition variability in select individual acoustic features depending on whether it is a flat harmonic**. The adjusted value of across rendition variation (i.e., CV) in acoustic features is plotted for flat harmonic (FlatHarmonic) and non-flat harmonic (NotFlatHarmonics) types sung by ASYN and GFP expressing groups. The CV of entropy (mean.entropy) for flat harmonic syllables was higher in the ASYN group (N = 9) compared to GFP control (N = 7) at 1, 2, and 3 mpi. Whereas, the CV of amplitude (mean.amplitude.adjusted) of the non-flat harmonic syllables (NotFlatHarmonics) was lower in the ASYN group (N = 46) compared to GFP control (N = 22) at 2 mpi with a trend detected at 3 mpi. Additionally, the CV of pitch goodness for these non-flat harmonic syllables was also higher in the ASYN group compared to GFP control at 3 mpi. Summary statistics provided in S2 Table. Reference Fig 7’s legend for explanation of boxplots. Statistical comparisons were made using a Wilcoxon Rank Sum Test. * indicates p < 0.05. # indicates 0.05 < p < 0.1.
