# Supplementary material for: Vocal changes in a zebra finch model of Parkinson’s disease characterized by alpha-synuclein overexpression in the song-dedicated anterior forebrain pathway
Source: PLoS One. 2022 May 4;17(5):e0265604. doi: 10.1371/journal.pone.0265604 (PMC9067653; doi:10.1371/journal.pone.0265604)
Supplement: S12 Fig — Remaining acoustic features whose within rendition variability score are not affected significantly within syllable types by αsyn overexpression. Reference Fig 7‘s legend for explanation of boxplots. Statistical comparisons were made using a Wilcoxon Rank Sum Test. (DOCX) [file pone.0265604.s012.docx]

**
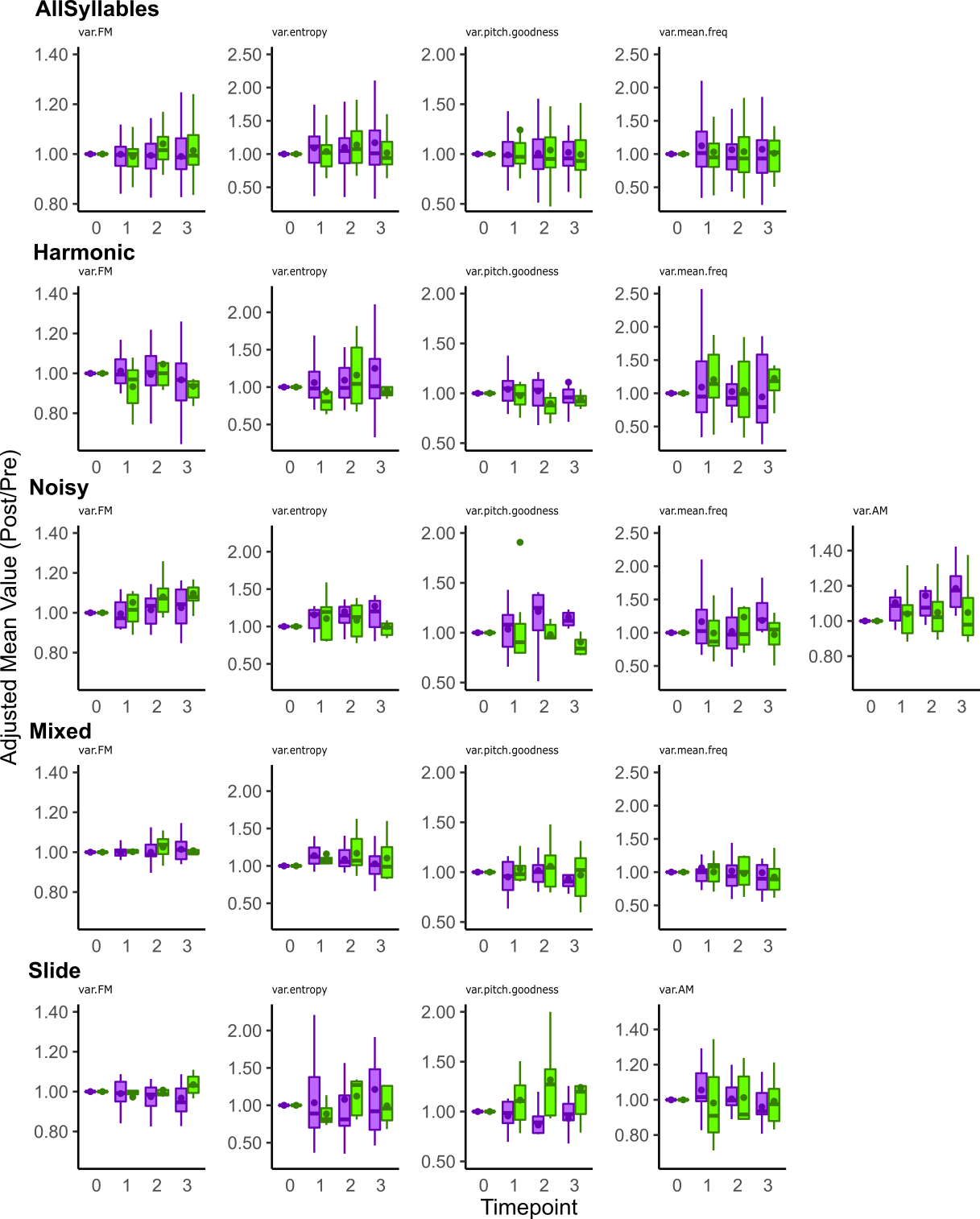
**

**S12. Within rendition variability of acoustic features for all, Harmonic, Noisy, Mixed, and Slide syllables that are not affected by αsyn** **overexpression.** Remaining acoustic features whose within rendition variability score are not affected significantly within syllable types by αsyn overexpression. Reference Fig 7’s legend for explanation of boxplots. Statistical comparisons were made using a Wilcoxon Rank Sum Test.
