# Supplementary material for: Vocal changes in a zebra finch model of Parkinson’s disease characterized by alpha-synuclein overexpression in the song-dedicated anterior forebrain pathway
Source: PLoS One. 2022 May 4;17(5):e0265604. doi: 10.1371/journal.pone.0265604 (PMC9067653; doi:10.1371/journal.pone.0265604)
Supplement: S13 Fig — The adjusted value of within rendition variation (i.e., var) in acoustic features is plotted for flat harmonic (FlatHarmonic) and non-flat harmonic (NotFlatHarmonics) syllables sung by ASYN and GFP expressing groups. No effects were detected for variance of individual acoustic features in the flat harmonic syllables, when we compared the ASYN group (N = 9) to GFP control (N = 7). However, the variance of frequency modulation (var.FM) for non-flat harmonic syllables (NotFlatHarmonics) was lower in the ASYN group compared to GFP control at 2 mpi with a trend detected at 3 mpi. Summary statistics provided in S2 Table. Reference Fig 7‘s legend for explanation of boxplots. Statistical comparisons were made using a Wilcoxon Rank Sum Test. * indicates p < 0.05. # indicates 0.05 < p < 0.1. (DOCX) [file pone.0265604.s013.docx]

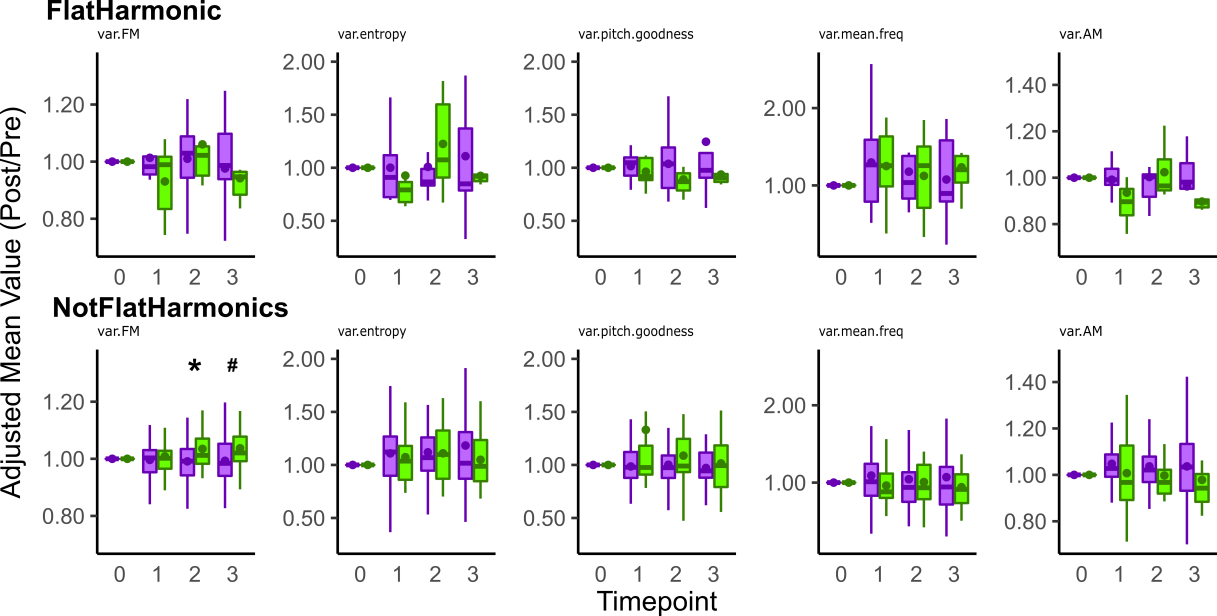


**S13. Asyn overexpression leads to monopitch of non-flat harmonic syllables.** The adjusted value of within rendition variation (i.e., var) in acoustic features is plotted for flat harmonic (FlatHarmonic) and non-flat harmonic (NotFlatHarmonics) syllables sung by ASYN and GFP expressing groups. No effects were detected for variance of individual acoustic features in the flat harmonic syllables, when we compared the ASYN group (N = 9) to GFP control (N = 7). However, the variance of frequency modulation (var.FM) for non-flat harmonic syllables (NotFlatHarmonics) was lower in the ASYN group compared to GFP control at 2 mpi with a trend detected at 3 mpi. Summary statistics provided in S2 Table. Reference Fig 7’s legend for explanation of boxplots. Statistical comparisons were made using a Wilcoxon Rank Sum Test. * indicates p < 0.05. # indicates 0.05 < p < 0.1
