# Supplementary material for: Vocal changes in a zebra finch model of Parkinson’s disease characterized by alpha-synuclein overexpression in the song-dedicated anterior forebrain pathway
Source: PLoS One. 2022 May 4;17(5):e0265604. doi: 10.1371/journal.pone.0265604 (PMC9067653; doi:10.1371/journal.pone.0265604)
Supplement: S14 Fig — The adjusted value of self-similarity scores (%Similarity and Accuracy) is plotted for All Syllables, then grouped by Harmonic, Mixed, Noisy, and Slide syllables sung by ASYN and GFP expressing groups. The accuracy score of mixed syllables was lower in the ASYN group (N = 18) compared to GFP control (N = 9) at 2 and 3 mpi. Summary statistics provided in S3 Table. Reference Fig 7‘s legend for explanation of boxplots. Statistical comparisons were made using a Wilcoxon Rank Sum Test. * indicates p < 0.05. (DOCX) [file pone.0265604.s014.docx]

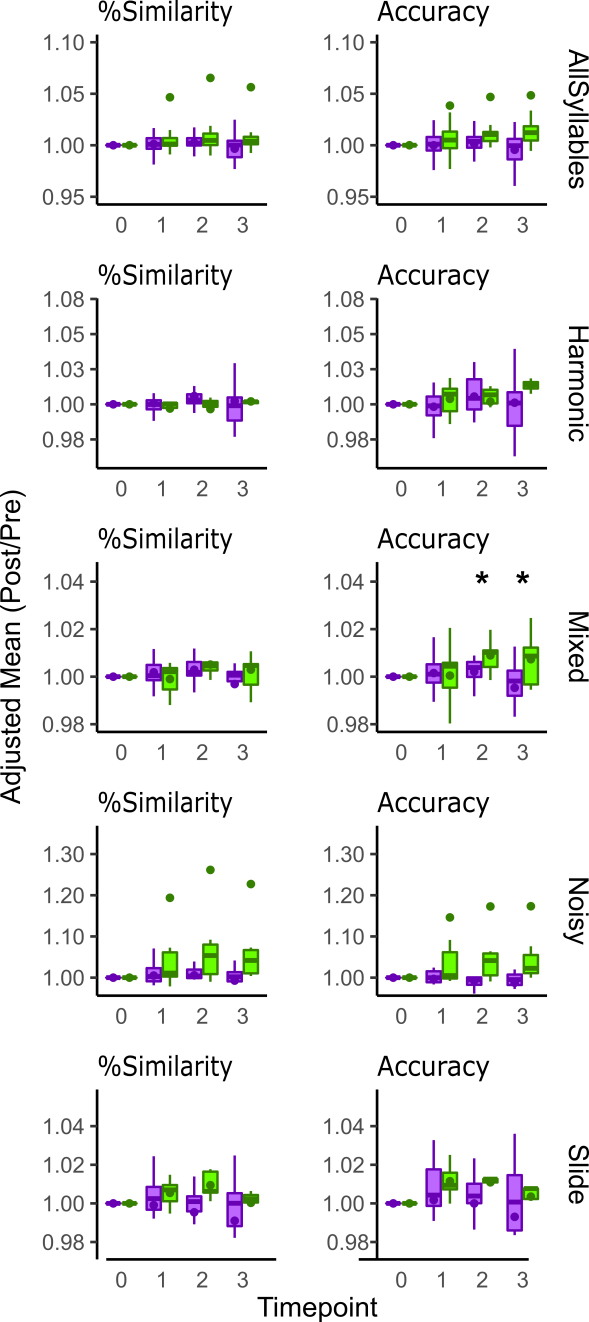


**S14.** **Asyn overexpression affects the self-accuracy score of mixed syllables.** The adjusted value of self-similarity scores (%Similarity and Accuracy) is plotted for All Syllables, then grouped by Harmonic, Mixed, Noisy, and Slide syllables sung by ASYN and GFP expressing groups. The accuracy score of mixed syllables was lower in the ASYN group (N = 18) compared to GFP control (N = 9) at 2 and 3 mpi. Summary statistics provided in S3 Table. Reference Fig 7’s legend for explanation of boxplots. Statistical comparisons were made using a Wilcoxon Rank Sum Test. * indicates p < 0.05.
