# Supplementary material for: Vocal changes in a zebra finch model of Parkinson’s disease characterized by alpha-synuclein overexpression in the song-dedicated anterior forebrain pathway
Source: PLoS One. 2022 May 4;17(5):e0265604. doi: 10.1371/journal.pone.0265604 (PMC9067653; doi:10.1371/journal.pone.0265604)
Supplement: S15 Fig — The adjusted value of self-similarity scores (%Similarity and Accuracy) is plotted for flat harmonic (FlatHarmonic) and non-flat harmonic (NotFlarHarmonic) syllables sung by ASYN or GFP expressing groups. No effects were found for the %Similary or Accuracy of either the flat harmonics (FlatHarmonics; NASYN = 9; NGFP = 7) or non-flat harmonics (NotFlatHarmonic; NASYN = 46; NGFP = 22). Summary statistics provided in S2 Table. Reference Fig 7‘s legend for explanation of boxplots. Statistical comparisons were made using a Wilcoxon Rank Sum Test. (DOCX) [file pone.0265604.s015.docx]

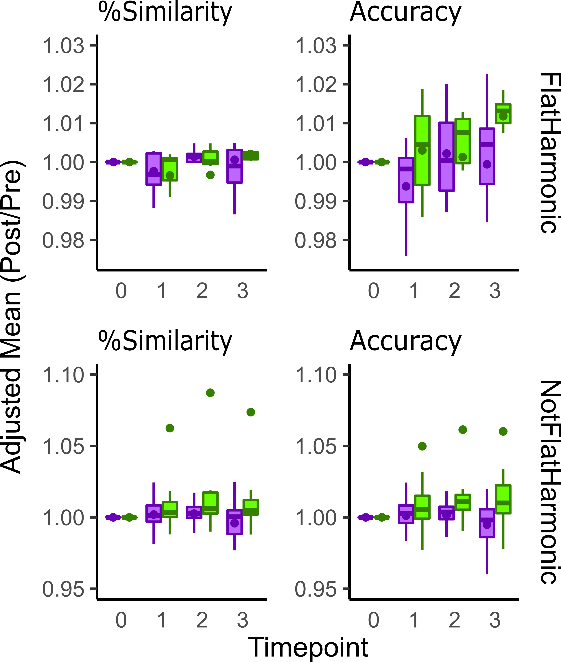


**S15. Asyn overexpression does not affect similarity scores of flat or non-flat harmonic syllables.** The adjusted value of self-similarity scores (%Similarity and Accuracy) is plotted for flat harmonic (FlatHarmonic) and non-flat harmonic (NotFlarHarmonic) syllables sung by ASYN or GFP expressing groups. No effects were found for the %Similary or Accuracy of either the flat harmonics (FlatHarmonics; N_ASYN_ = 9; N_GFP_ = 7) or non-flat harmonics (NotFlatHarmonic; N_ASYN_ = 46; N_GFP_ = 22). Summary statistics provided in S2 Table. Reference Fig 7’s legend for explanation of boxplots. Statistical comparisons were made using a Wilcoxon Rank Sum Test.
