# Supplementary material for: Vocal changes in a zebra finch model of Parkinson’s disease characterized by alpha-synuclein overexpression in the song-dedicated anterior forebrain pathway
Source: PLoS One. 2022 May 4;17(5):e0265604. doi: 10.1371/journal.pone.0265604 (PMC9067653; doi:10.1371/journal.pone.0265604)
Supplement: S1 Table — Conditions and molecular weights correspond to values referenced in S5 Fig. (DOCX) [file pone.0265604.s016.docx]

| **Summary Table of Soluble & Insoluble ASYN Levels in VSP** | | | | | |  |  |  |  |
| --- | --- | --- | --- | --- | --- | --- | --- | --- | --- |
|  |  | **Soluble** | | | | **Insoluble** | | | |
| **Mol. Wt. (KDa)** | **Condition** | **N** | **mean** | **median** | **sd** | **N** | **mean** | **median** | **sd** |
| **50** | ASYN | 4 | 5.68E-02 | 1.04E-03 | 1.12E-01 | 4 | 1.53E-01 | 4.40E-02 | 2.42E-01 |
|  | GFP | 4 | 9.71E-04 | 9.06E-04 | 4.64E-04 | 4 | 1.30E-02 | 5.25E-03 | 1.81E-02 |
|  | NS | 4 | 3.96E-03 | 4.10E-03 | 2.30E-03 | 4 | 5.68E-02 | 5.78E-02 | 5.63E-03 |
| **Multimer** | ASYN | 4 | 8.80E-01 | 1.79E-01 | 1.45E+00 | 4 | 5.98E-01 | 1.16E-01 | 9.85E-01 |
|  | GFP | 4 | 1.02E-01 | 1.04E-01 | 2.69E-02 | 4 | 9.97E-02 | 8.98E-02 | 4.11E-02 |
|  | NS | 4 | 2.15E-01 | 2.10E-01 | 5.86E-02 | 4 | 1.76E-01 | 1.70E-01 | 3.39E-02 |
| **Total** | ASYN | 4 | 1.06E+00 | 2.12E-01 | 1.75E+00 | 4 | 9.69E-01 | 1.75E-01 | 1.62E+00 |
|  | GFP | 4 | 1.11E-01 | 1.14E-01 | 2.47E-02 | 4 | 1.25E-01 | 1.19E-01 | 5.48E-02 |
|  | NS | 4 | 2.44E-01 | 2.47E-01 | 5.70E-02 | 4 | 2.48E-01 | 2.49E-01 | 3.79E-02 |

**S1 Table. Summary statistics of normalized soluble and insoluble αsyn levels in VSP grouped by condition and molecular weight (Mol. Wt.)**. Conditions and molecular weights correspond to values referenced in S5 Fig.
