# Supplementary material for: Vocal changes in a zebra finch model of Parkinson’s disease characterized by alpha-synuclein overexpression in the song-dedicated anterior forebrain pathway
Source: PLoS One. 2022 May 4;17(5):e0265604. doi: 10.1371/journal.pone.0265604 (PMC9067653; doi:10.1371/journal.pone.0265604)
Supplement: S2 Table — Reference Table 5‘s legend for additional variable names. * indicates p < 0.05. # indicates 0.05 < p < 0.1. (DOCX) [file pone.0265604.s017.docx]

| **Summary Table of Mean, Across- and Within-Rendition Variability for Flat and Non-Flat Harmonic Syllables** | | | | | | | | | | |  |  |
| --- | --- | --- | --- | --- | --- | --- | --- | --- | --- | --- | --- | --- |
|  |  |  |  | **ASYN** | | | | **GFP Control** | | | |  |
|  | **Variable** | **Type** | **Month** | **N** | **mean** | **median** | **sd** | **N** | **mean** | **median** | **sd** | **p** |
| **Mean** | Duration | Not Flat | 1 | 46 | 9.86E-01 | 9.91E-01 | 6.36E-02 | 22 | 1.04E+00 | 1.02E+00 | 8.94E-02 | * |
|  |  |  | 2 | 46 | 9.81E-01 | 9.93E-01 | 7.10E-02 | 22 | 1.05E+00 | 1.02E+00 | 9.31E-02 | * |
|  |  |  | 3 | 46 | 9.79E-01 | 9.80E-01 | 7.53E-02 | 22 | 1.02E+00 | 1.01E+00 | 7.42E-02 | * |
| **Across** | Entropy | Flat | 1 | 9 | 1.11E+00 | 1.04E+00 | 2.40E-01 | 7 | 7.73E-01 | 8.11E-01 | 1.02E-01 | * |
|  |  |  | 2 | 9 | 1.00E+00 | 1.05E+00 | 1.53E-01 | 7 | 7.95E-01 | 8.37E-01 | 1.98E-01 | * |
|  |  |  | 3 | 9 | 1.01E+00 | 1.02E+00 | 2.92E-01 | 7 | 7.05E-01 | 6.79E-01 | 1.33E-01 | * |
|  | Amplitude | Not Flat | 1 | 46 | 1.06E+00 | 9.78E-01 | 3.54E-01 | 22 | 1.10E+00 | 9.63E-01 | 4.57E-01 |  |
|  |  |  | 2 | 46 | 9.31E-01 | 8.95E-01 | 2.25E-01 | 22 | 1.21E+00 | 1.19E+00 | 3.44E-01 | * |
|  |  |  | 3 | 46 | 9.36E-01 | 8.70E-01 | 2.52E-01 | 22 | 1.05E+00 | 1.04E+00 | 2.66E-01 | # |
|  | Goodness | Not Flat | 1 | 46 | 1.02E+00 | 1.00E+00 | 3.24E-01 | 22 | 1.21E+00 | 1.01E+00 | 7.31E-01 |  |
|  |  |  | 2 | 46 | 9.86E-01 | 9.66E-01 | 2.28E-01 | 22 | 9.03E-01 | 9.15E-01 | 2.21E-01 |  |
|  |  |  | 3 | 46 | 9.77E-01 | 9.59E-01 | 2.00E-01 | 22 | 8.54E-01 | 8.14E-01 | 2.33E-01 | * |
| **Within** | Freq Mod | Not Flat | 1 | 46 | 9.96E-01 | 1.00E+00 | 6.39E-02 | 22 | 1.01E+00 | 1.00E+00 | 1.08E-01 |  |
|  |  |  | 2 | 46 | 9.91E-01 | 9.92E-01 | 6.99E-02 | 22 | 1.03E+00 | 1.01E+00 | 7.66E-02 | * |
|  |  |  | 3 | 46 | 9.93E-01 | 9.84E-01 | 9.99E-02 | 22 | 1.04E+00 | 1.02E+00 | 8.25E-02 | # |

**S2 Table. Summary statistics of mean, across- and within-rendition variability scores grouped by Flat Harmonic type and experimental condition.** Reference Table 5’s legend for additional variable names. * indicates p < 0.05. # indicates 0.05 < p < 0.1.
