# Supplementary material for: Vocal changes in a zebra finch model of Parkinson’s disease characterized by alpha-synuclein overexpression in the song-dedicated anterior forebrain pathway
Source: PLoS One. 2022 May 4;17(5):e0265604. doi: 10.1371/journal.pone.0265604 (PMC9067653; doi:10.1371/journal.pone.0265604)
Supplement: S3 Table — * indicates p < 0.05. # indicates 0.05 < p < 0.1. (DOCX) [file pone.0265604.s018.docx]

| Summary Table of Accuracy Scores for Mixed Syllables from ASYN and GFP Control Birds | | | | | | |  |  |  |
| --- | --- | --- | --- | --- | --- | --- | --- | --- | --- |
|  | **ASYN** | | | | **GFP Control** | | | |  |
| Month | **N** | **mean** | **median** | **sd** | **N** | **mean** | **median** | **sd** | **p** |
| 1 | 18 | 1.00E+00 | 1.00E+00 | 6.65E-03 | 9 | 1.00E+00 | 1.00E+00 | 1.42E-02 |  |
| 2 | 18 | 1.00E+00 | 1.00E+00 | 6.33E-03 | 9 | 1.01E+00 | 1.01E+00 | 7.06E-03 | * |
| 3 | 18 | 9.95E-01 | 9.98E-01 | 1.23E-02 | 9 | 1.01E+00 | 1.01E+00 | 1.02E-02 | * |

**S3 Table. Summary statistics of Mixed syllable accuracy scores grouped by experimental condition.** * indicates p < 0.05. # indicates 0.05 < p < 0.1.
