# Supplementary material for: Vocal changes in a zebra finch model of Parkinson’s disease characterized by alpha-synuclein overexpression in the song-dedicated anterior forebrain pathway
Source: PLoS One. 2022 May 4;17(5):e0265604. doi: 10.1371/journal.pone.0265604 (PMC9067653; doi:10.1371/journal.pone.0265604)

Data for S2 Fig

Cropped

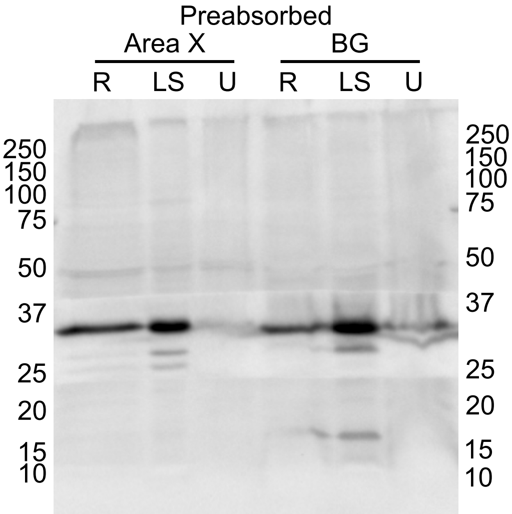

Raw

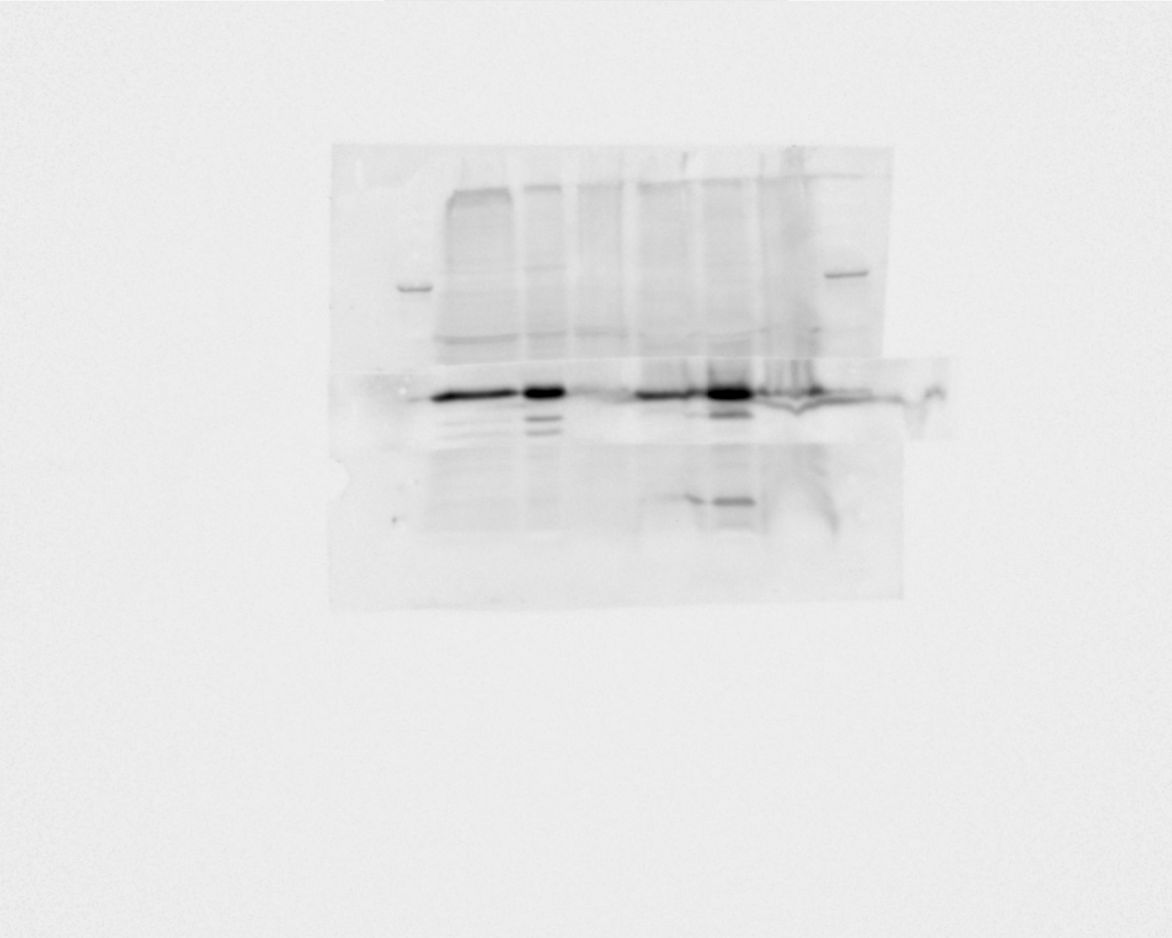

Data for S2 Fig

Cropped

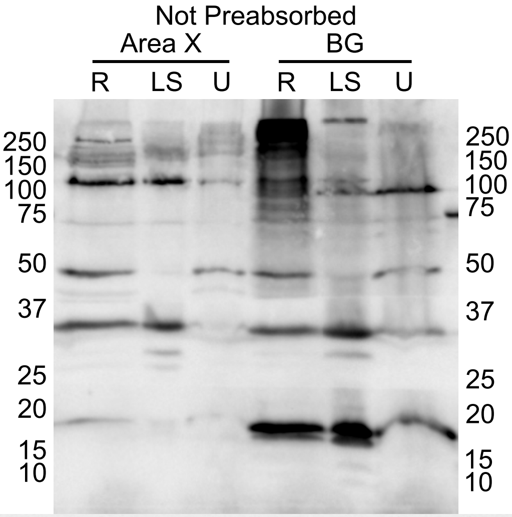

Raw

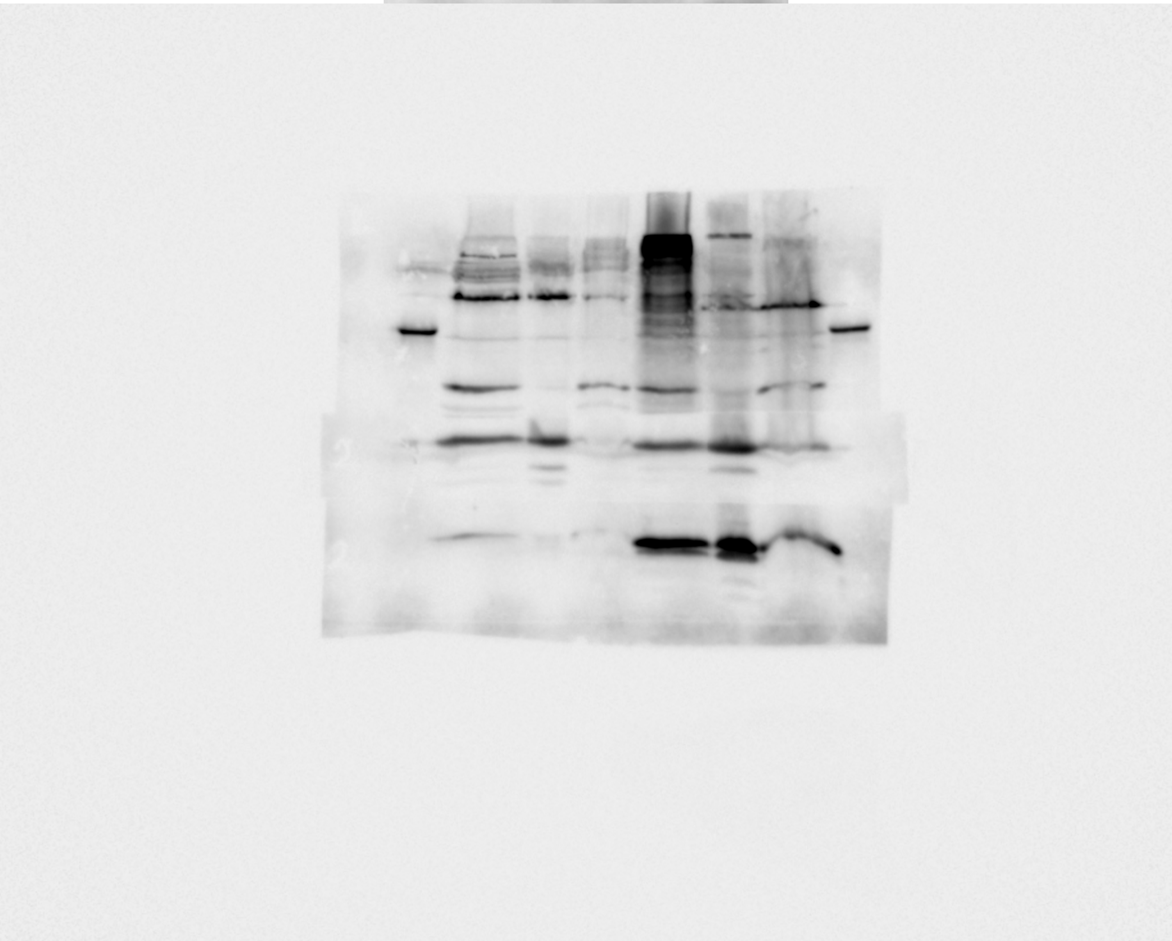

Data for S3 Fig

Cropped

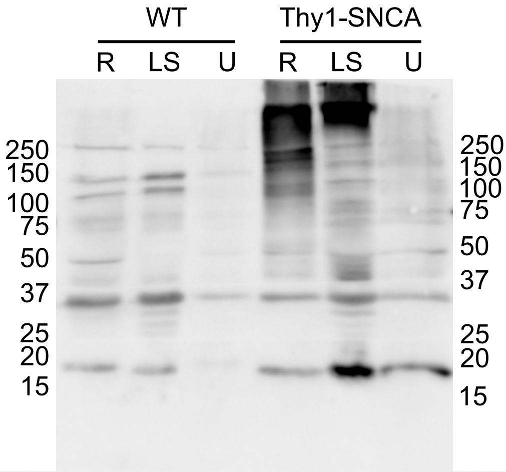

Raw

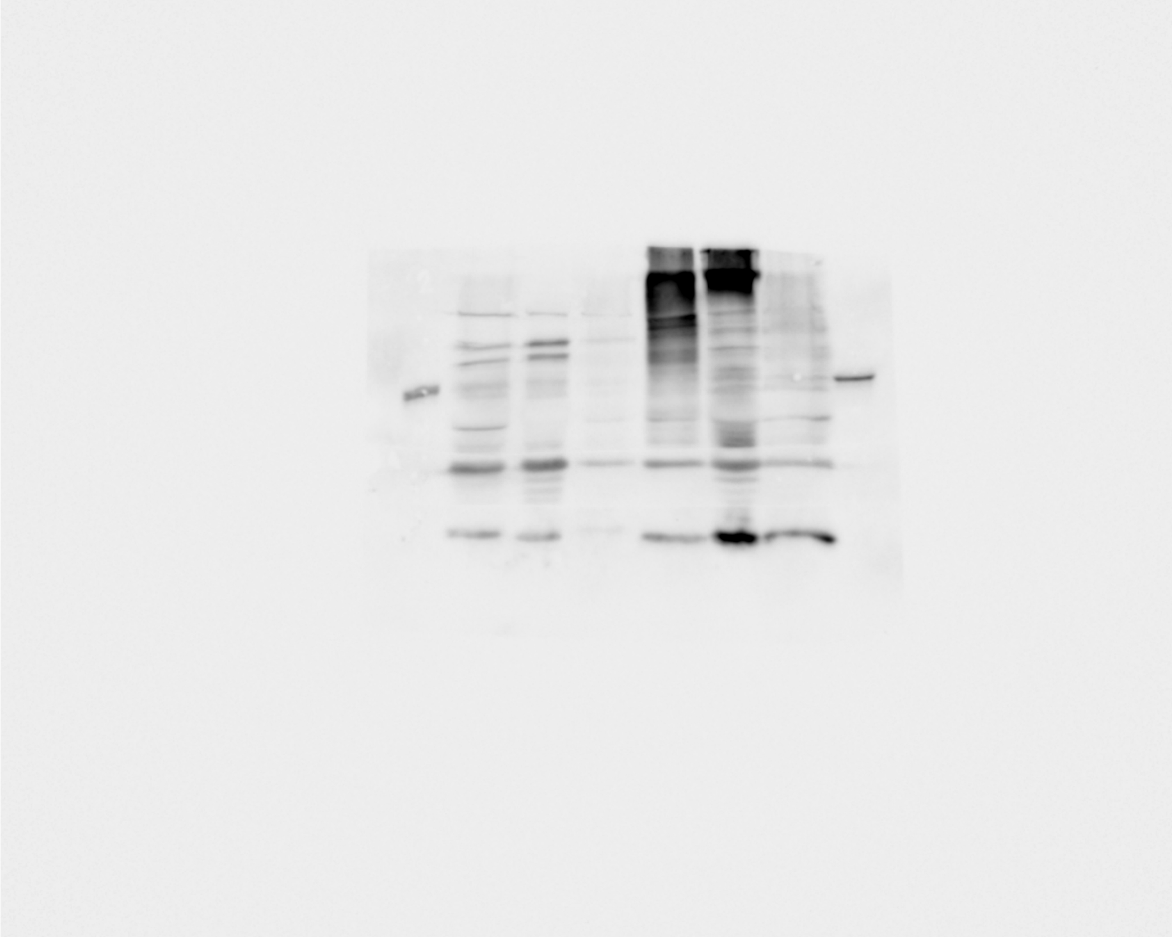

Data for Fig 5

Cropped

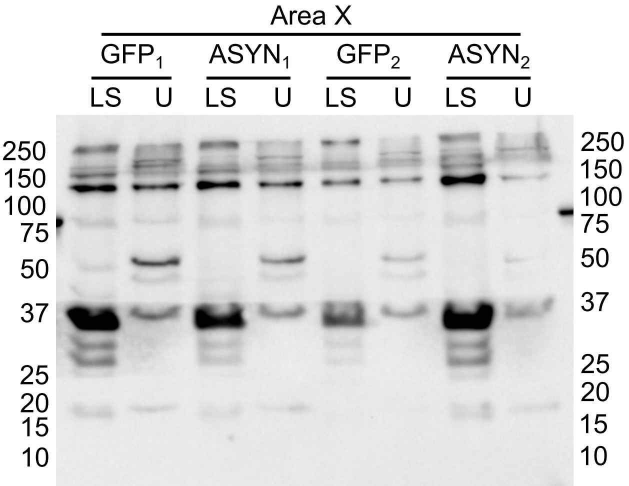

Raw

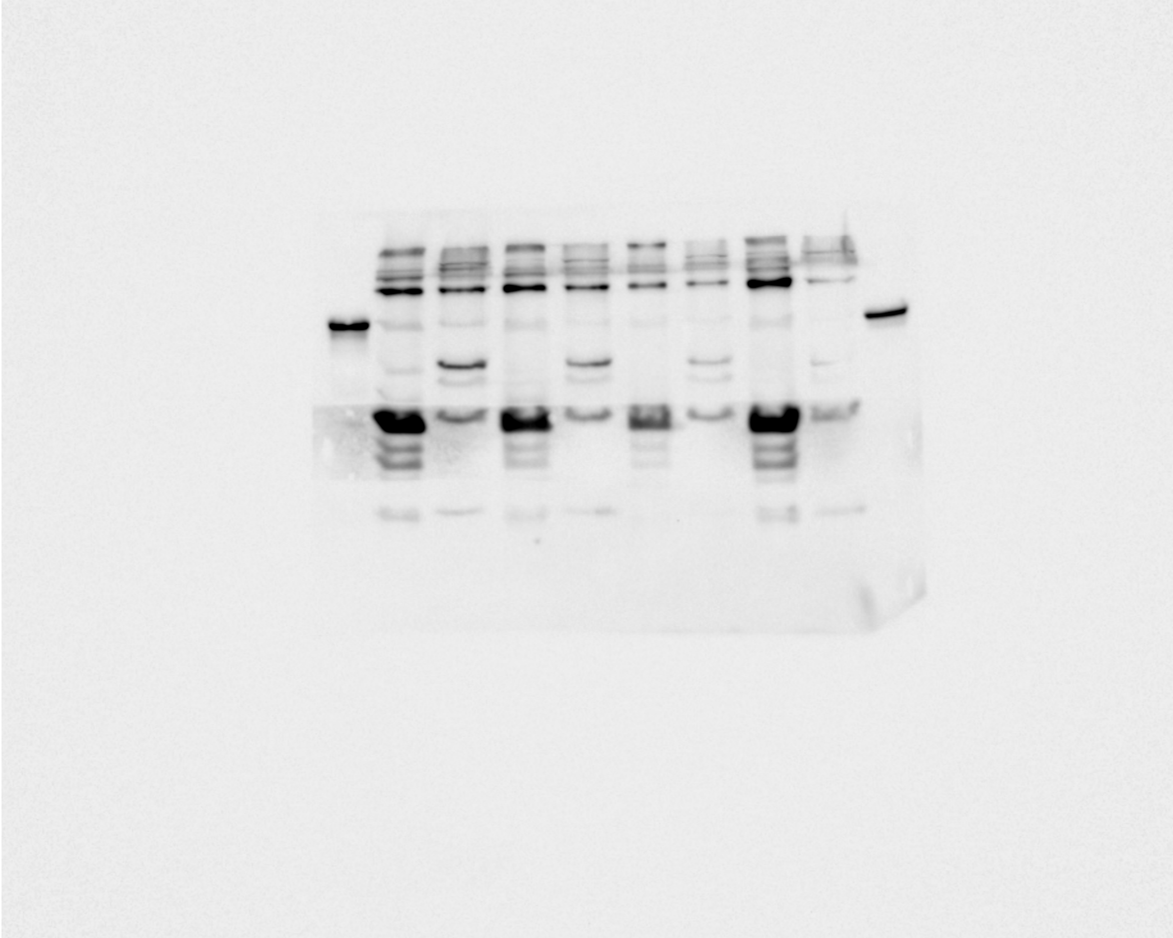

Data for Fig 5

Cropped

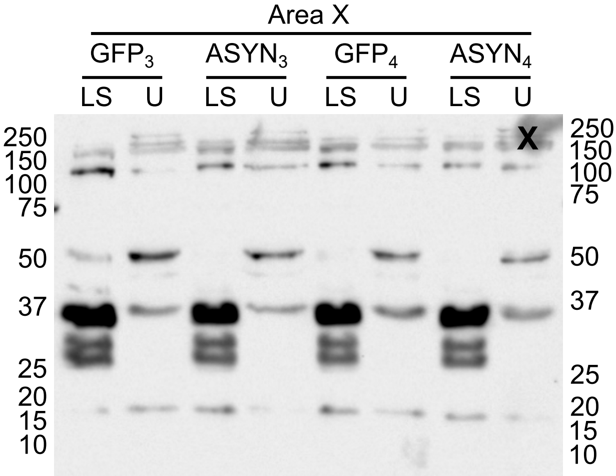

Raw

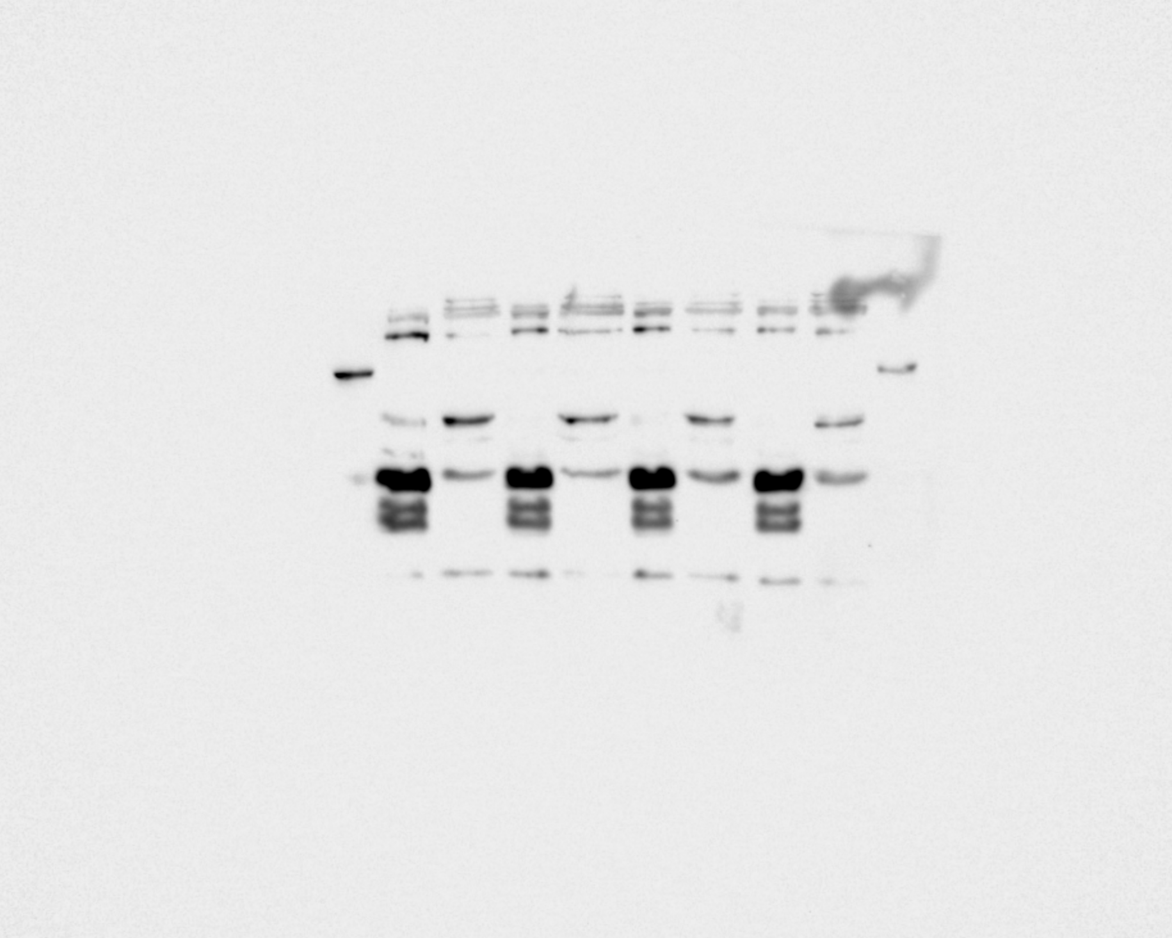

Data for Fig 5

Cropped

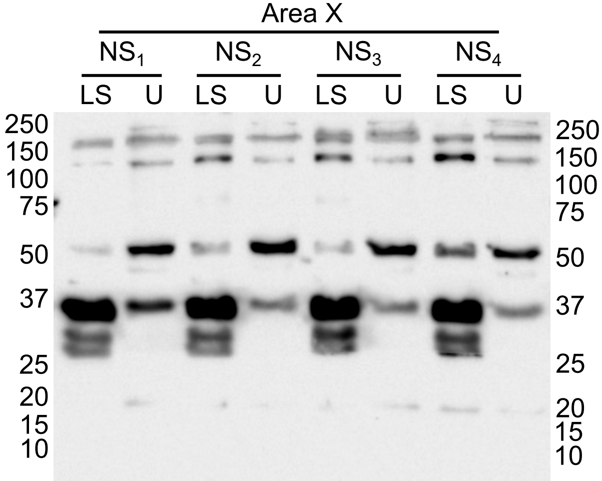

Raw

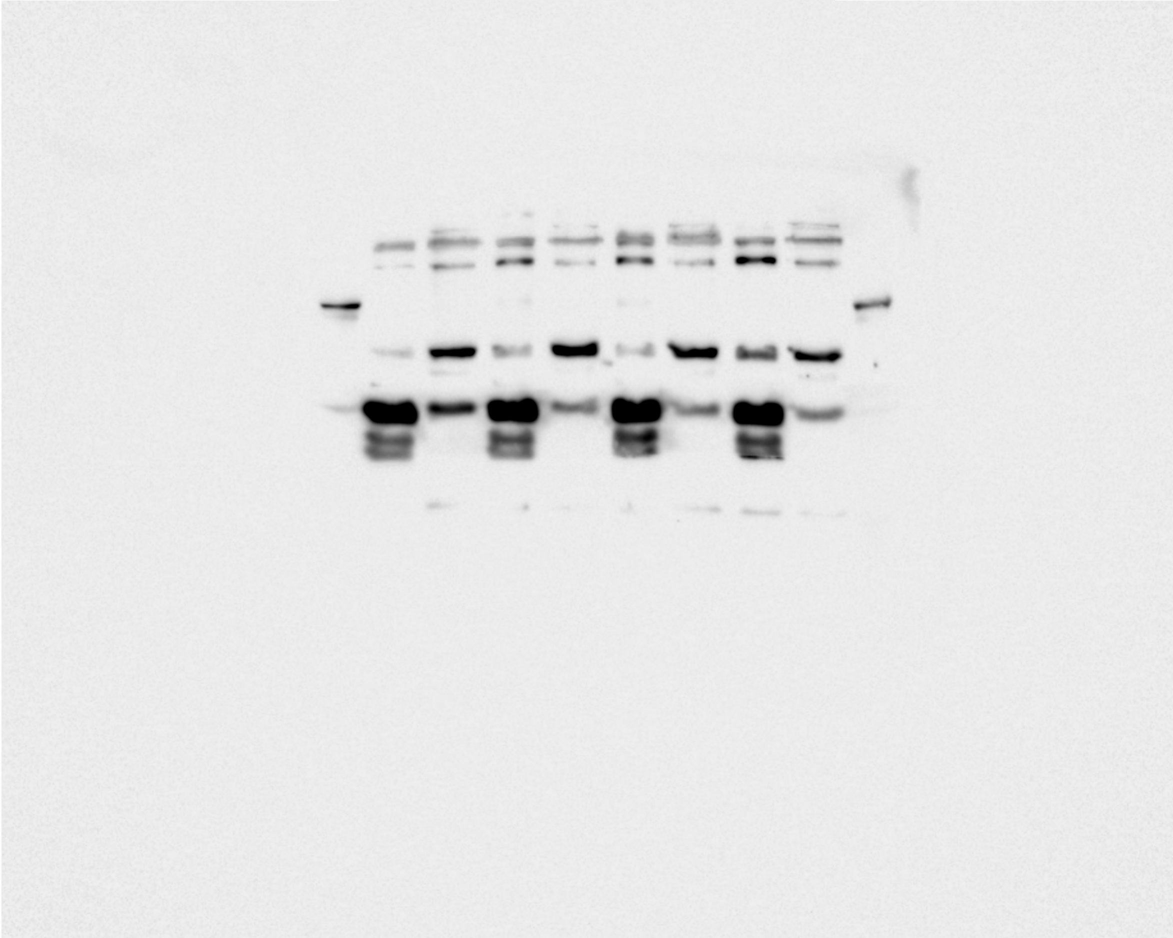

Data for S5 Fig

Cropped

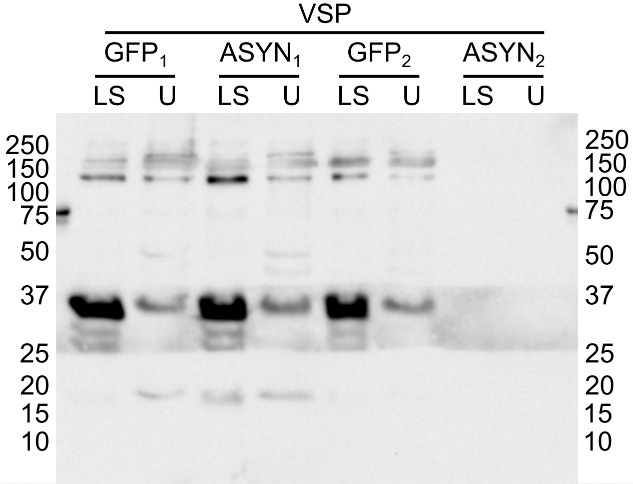

Raw

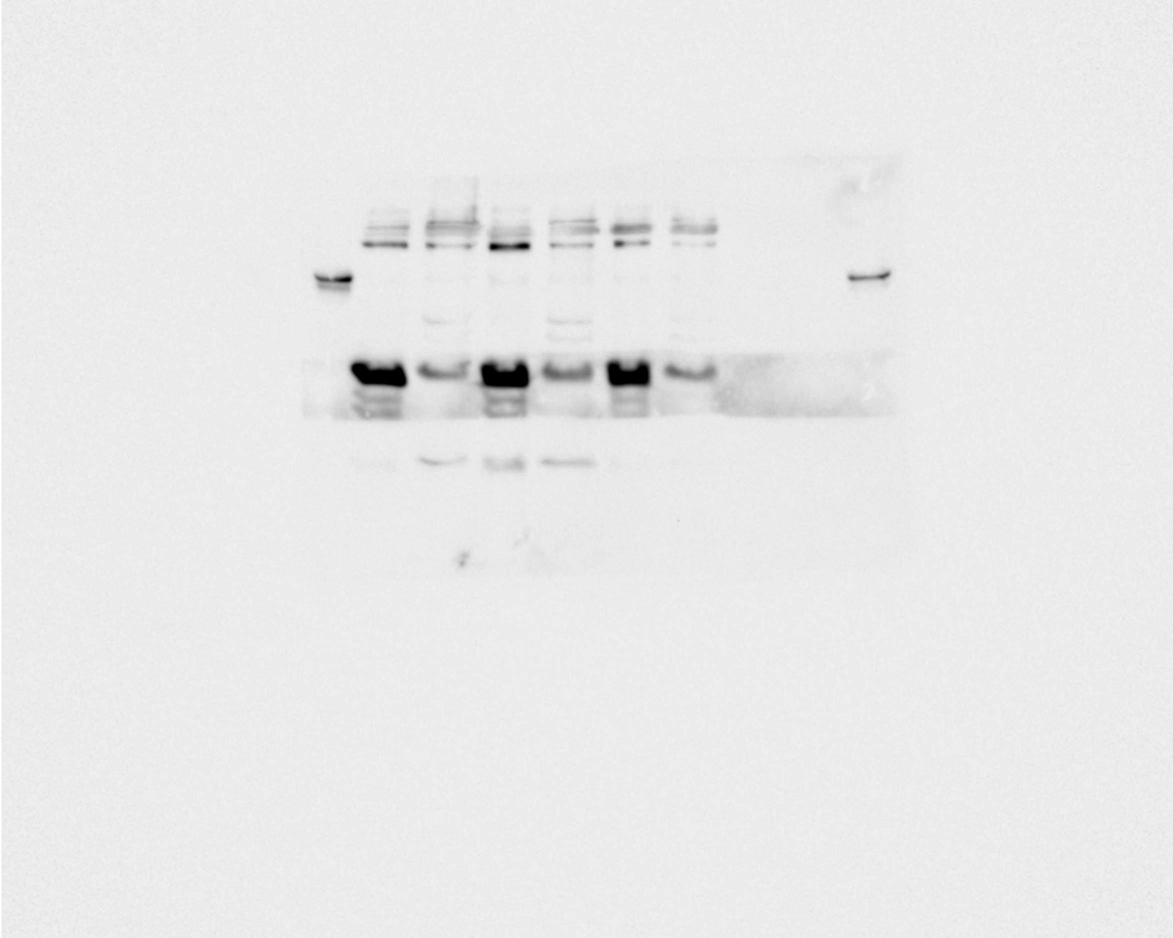

Data for S5 Fig

Cropped

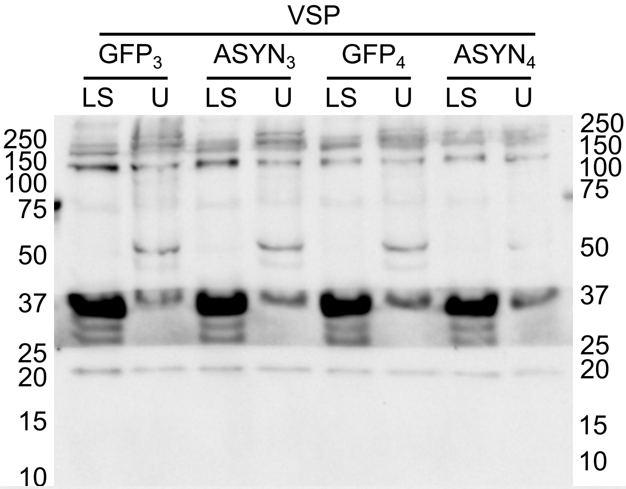

Raw

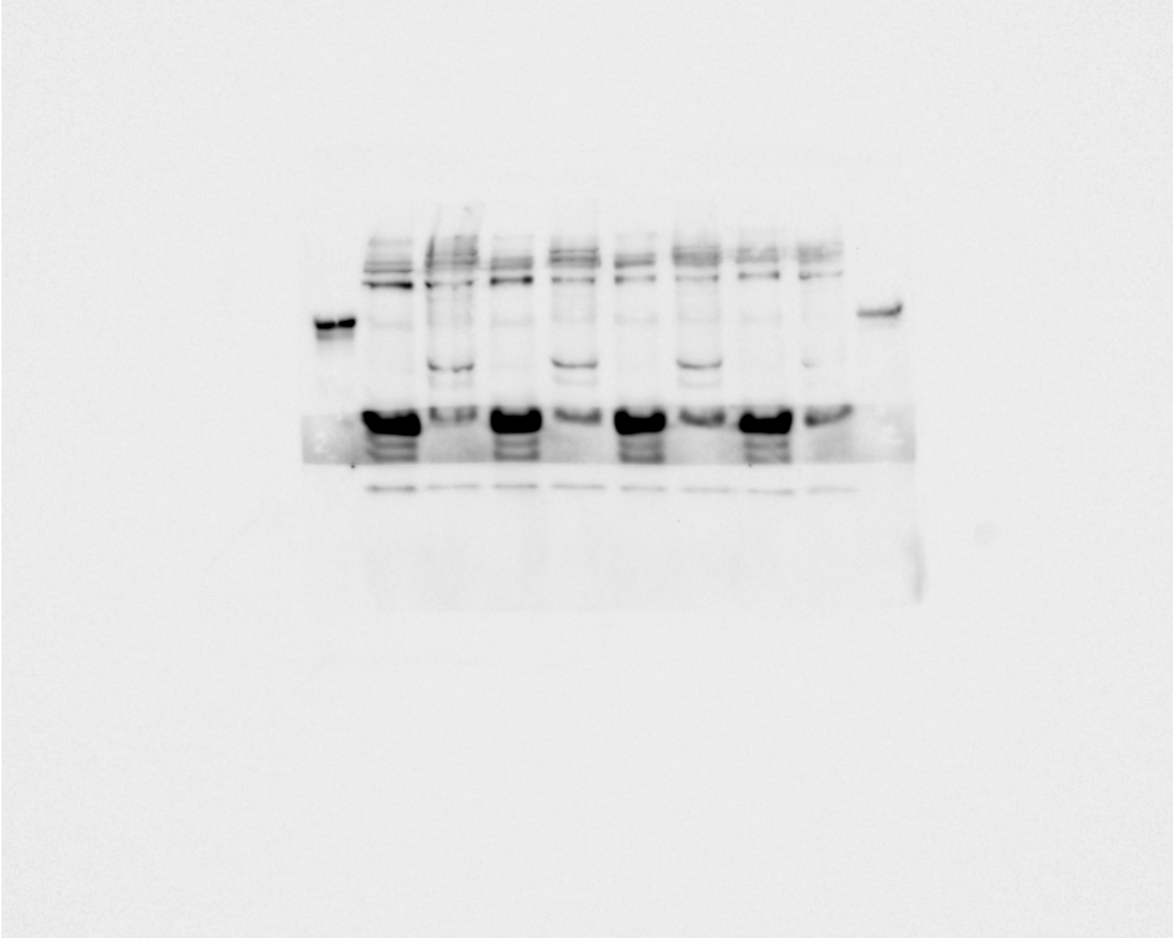

Data for S5 Fig

Cropped

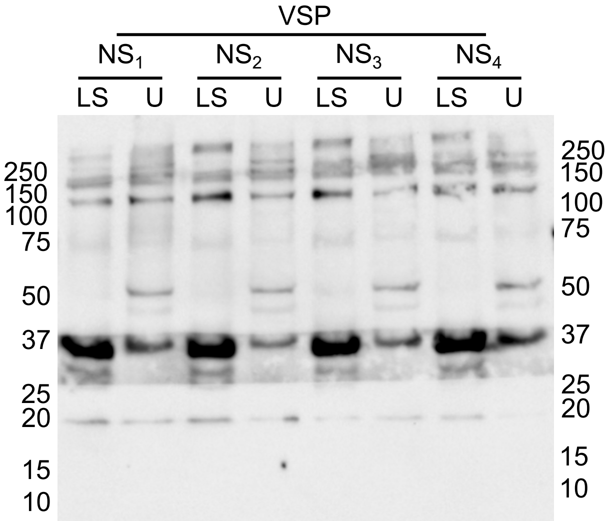

Raw

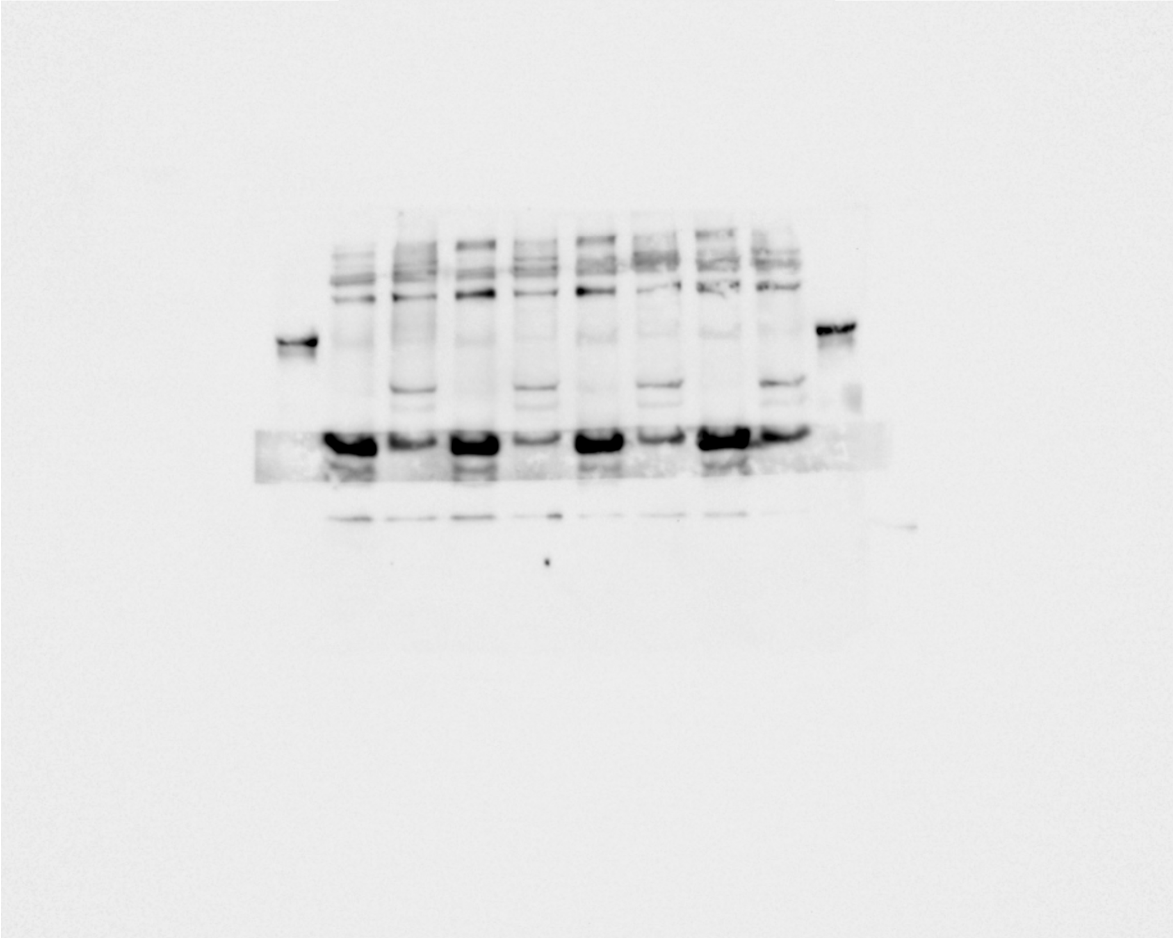

Supplement: S1 Raw images — (PDF) [file pone.0265604.s019.pdf]
